# Combining EEG and eye-tracking for cognitive and physiological states monitoring: a systematic review

**DOI:** 10.3389/fnrgo.2025.1736672

**Published:** 2026-01-29

**Authors:** Maria Rivas-Vidal, Alberto Calvo Cordoba, Cecilia E. García Cena, Fernando Daniel Farfán

**Affiliations:** 1 Escuela Técnica Superior de Ingenieros Industriales, Universidad Politécnica de Madrid, Madrid, Spain; 2 Centro de Automática y Robótica UPM-CSIC, Escuela Técnica Superior de Ingeniería y Diseño Industrial, Universidad Politécnica de Madrid, Madrid, Spain; 3 Indra Sistemas, S.A., Madrid, Spain; 4 Instituto de Bioingeniería, Universidad Miguel Hernández, Alicante, Spain; 5 Laboratorio de Investigación en Neurociencias y Tecnologías Aplicadas (LINTEC), Departamento de Bioingeniería, Facultad de Ciencias Exactas y Tecnología, Universidad Nacional de Tucumán, Tucumán, Argentina; 6 Instituto Superior de Investigaciones Biológicas (INSIBIO), Consejo Nacional de Investigaciones Científicas y Técnicas (CONICET), Tucumán, Argentina

**Keywords:** drowsiness, fatigue, mental workload, mind wandering, perception, situational awareness, stress, vigilance

## Abstract

Monitoring situational awareness is critical in highly demanding environments where sustained attention and vigilance are essential for safety and performance. Electroencephalography (EEG) and eye-tracking (ET) provide complementary insights into the perceptual layer of situational awareness, capturing neural and ocular signatures of information processing, attention, and fatigue. However, studies have typically examined perception-related conditions such as workload, fatigue, stress, and drowsiness in isolation, limiting understanding of their shared and distinct physiological patterns. This systematic review synthesizes findings from studies that recorded EEG and ET concurrently to investigate perception-related conditions. Following the PRISMA 2020 statement, five databases were searched, and 47 studies met the inclusion criteria. The most frequently reported EEG features included theta, alpha, and beta activity, while ET metrics commonly involved fixation patterns, pupil diameter, blink dynamics, and percentage of eyes closed (PERCLOS). Across studies, fatigue, mental workload, and stress exhibited overlapping physiological signatures, although multimodal data helped differentiate these closely related states. Drowsiness and vigilance decrement appeared along a shared continuum, with microsleeps showing distinct physiological profiles. Classification models generally achieved higher accuracy when integrating EEG and ET features than when using either modality alone. This review highlights the potential of concurrent EEG and ET monitoring for improving the detection of perception-related conditions and for disambiguating closely related states. These findings also support the need for standardized multimodal protocols and real-time multimodal classification models to strengthen cognitive-state monitoring, operational performance, and error prevention in high-risk domains.

## 1 Introduction

Situational awareness is fundamental for optimal human performance in complex and highly demanding fields such as military, aerospace, and industrial operations. According to Endsley's model (Endsley, 1995), its first layer, perception, depends on attention and vigilance processes to filter, prioritize, and maintain focus on critical information over time. Failures at this perceptual stage can result in significant adverse outcomes, especially in environments that require continuous monitoring, rapid decision-making, and sustained alertness (Endsley, 1995; Mohammadfam et al., 2021; Naderpour et al., 2015).

Cognitive and physiological states, such as mental workload, fatigue, and drowsiness, affect arousal and resource allocation, which in turn alter perceptual accuracy. These conditions are also highly interrelated. For instance, prolonged exposure to stress or mental workload can lead to mental fatigue (Holm et al., 2009; Kunasegaran et al., 2023). Similarly, alterations in sleep patterns or experiences of boredom can result in drowsiness (Chowdhury et al., 2018; Guede-Fernandez et al., 2019; Rafid et al., 2020) or lead to periods of inattention due to mind wandering (Smallwood and Schooler, 2015). Together, these factors contribute to impaired sustained attention or vigilance. Thus, identifying these conditions and developing objective, real-time measurement and prediction systems is critical for enhancing safety, training, and performance outcomes (Endsley, 2001; Endsley and Robertson, 2000; Graafland et al., 2015; Rodriguez et al., 2017).

Two non-invasive and wearable sensing modalities, electroencephalography (EEG) and eye tracking (ET), have emerged as promising tools for assessing cognitive states in operational environments. EEG provides direct measurements of brain dynamics, sensitive to fluctuations in attention, working memory load, fatigue, and arousal (Cohen, 2017; Ismail and Karwowski, 2020; Jackson and Bolger, 2014; Kirschstein and Köhling, 2009; Soufineyestani et al., 2020). In parallel, ET offers insights into visual attention and processing, with metrics related to eye gaze, eyelid opening, and pupil dilation serving as established behavioral indicators (King et al., 2019; Martinez-Marquez et al., 2021; Popa et al., 2015; Skaramagkas et al., 2023; Ziv, 2016).

Based on their complementary capabilities, integrating EEG and ET could facilitate the development of more robust and discriminative monitoring systems that capture the multimodal dynamics underlying perception and situational awareness. Although hybrid EEG-ET approaches have been reviewed in other disciplines such as neuromarketing (Kalaganis et al., 2021) or brain-computer interface (Hong and Khan, 2017), previous reviews addressing perception-related conditions have primarily surveyed a broad range of monitoring techniques and organized findings by individual signal modalities and within condition-specific scopes. For example, those focused on drowsiness, mental fatigue, vigilance, stress, mental workload, and mind wandering largely summarized physiological measures in a unimodal manner, with limited synthesis of multimodal integration (Doudou et al., 2020; Kunasegaran et al., 2023; Kuvar et al., 2023; Mehrabi and Kim, 2022; Tao et al., 2019; Torkamani-Azar et al., 2022). On the other hand, reviews focusing on situational awareness report few or no multimodal studies, despite acknowledging multimodal approaches for mental workload and their potential for detection improvement (Elshafei and Romano, 2023; Zhang et al., 2020). Consequently, existing literature provides valuable condition-specific overviews but offer limited insight into how neural and ocular signals jointly reflect and differentiate closely related perception-related cognitive states.

To address this gap, the present systematic review aims to: (1) identify and synthesize studies that combine EEG and ET to characterize perception-related conditions, including stress, vigilance, drowsiness, fatigue, mental workload, and mind wandering; (2) delineate the primary EEG and ET metrics utilized in these studies; and (3) evaluate the effectiveness of multimodal vs. single-sensor approaches for monitoring these states by comparing the performance of machine learning classifiers. Ultimately, this review seeks to inform the development of a unified framework for cognitive-state monitoring, intended to enhance perceptual performance and safety in complex, real-world environments.

The theoretical background in which this study was based has been recently treated by the main authors in a previous work (Córdoba et al., 2024). From the proposed cognitive model of perception, only those conditions that have been formerly assessed by both EEG and ET sensing modalities have been selected for this review.

## 2 Methods

This systematic review followed the PRISMA 2020 statement (Page et al., 2021).

### 2.1 Eligibility criteria

Studies were eligible if they co-registered cerebral and ocular activities using EEG and ET technologies in any of the following conditions: stress, vigilance, sleep, drowsiness, physical fatigue, mental fatigue, mental workload, or mind wandering. To be included, studies had to quantify these states with specific metrics derived from both eye and brain data. Only studies published in English were selected.

Studies were excluded if they were reviews, meta-analyses, theses, dissertations, or posters. Additional exclusions were applied to studies involving pediatric or clinical populations, non-condition-specific tasks (e.g., reading, learning, robotic control), pharmacological interventions (e.g., caffeine, drugs, anesthesia), animal studies, and research on artifact correction.

Clinical and pediatric samples were excluded to avoid confounding factors related to pathological or developmental alterations in brain or ocular functions that would not be representative of the target population. The rationale for restricting brain signal recording to electroencephalography was that other techniques, such as functional near-infrared spectroscopy (fNIRS) or magnetic resonance imaging (MRI), are less compatible with eye-tracking setups or unsuitable in real-world workplace environments due to hardware constraints.

### 2.2 Information sources and search strategy

Electronic searches were conducted across five databases (PubMed, Scopus, Cochrane Library (CENTRAL), IEEE Xplore, and Web of Science) using the following query: [(eeg) AND (“eye tracking”)] AND [(stress) OR (vigilance) OR (sleep) OR (drowsiness) OR (“physical fatigue”) OR (“mental fatigue”) OR (“mental workload”) OR (“mind wandering”)]. This query was designed to capture studies that co-registered brain activity (EEG) and eye movements across the defined relevant conditions to our research, such as stress, fatigue, and attention states. Searches covered all records from database inception to October 2024.

#### 2.2.1 Selection process

After completing the electronic searches, all records were imported into the Systematic Review Accelerator (SRA) Deduplicator tool (Forbes et al., 2024) to identify and remove duplicate studies. The deduplication results were then manually reviewed by the first author to confirm accuracy.

Following deduplication, first and second authors screened the titles and abstracts of the remaining studies for relevance. During the title screening phase, studies were excluded if they focused on ineligible populations (e.g., children or clinical populations with specific diseases), targeted condition-unrelated tasks, effects of pharmacological agents, or if they were reviews, meta-analyses, or other secondary sources. The following abstract screening applied these same criteria to any additional exclusions not evident in the title and excluding studies that mentioned only one of the two required technologies (EEG or ET) or those lacking references to any of the targeted conditions. Studies primarily investigating interactions with computers or interfaces through EEG and ET rather than assessing the cognitive or physiological conditions of interest were also excluded. Additionally, six studies were excluded due to unavailable abstracts.

For those that passed the initial screening, full texts were obtained and reviewed by the same two reviewers to confirm they met all predefined eligibility criteria, including the use of both EEG and ET technologies to co-register cerebral and ocular activities and the presence of specific metrics used to quantify the targeted conditions.

Disagreements between reviewers were resolved through discussion or, if necessary, by consulting the other two reviewers. Studies meeting all criteria were included in the final analysis.

### 2.3 Data collection process and data items

Three data extraction tables were generated in Microsoft Excel (available as PDFs in the Supplementary material) to collect information from the studies that met the inclusion criteria. Each table captured specific dimensions of the experiments, with the third one focusing exclusively on collecting information on those studies that explored the classification of the conditions.

The first table captured experimentation details such as the number and characteristics of the participants involved in the studies, the condition elicited, and the type of data collected (e.g., physiological, cognitive/performance-based, or subjective measures). Additionally, the number of EEG channels and their configuration or the type of signal ET (gaze, eyelid opening, or pupil dilation) were collected.

For the second table, extracted features from EEG and ET and available information from other sensors or qualitative or subjective tests were collected. Additionally, if available, the table synthesized information on how the target condition influenced the extracted features across the different data modalities.

Finally, the third table collected information on conditions' classification based on information from physiological signals. Available data such as labels, types of classifiers, performance metrics and validation methods used were included. If available, information on the comparison between classification using single physiological signal modality or its combination was also included.

Data extraction was performed by the first author using the predefined data extraction tables. The data fields included in these tables were established through consensus with other reviewers to ensure completeness and consistency. No automation tools were used in the process. If any data field was not reported in a study, it was marked as “Not Available” (NA) in the extraction tables. While every effort was made to minimize errors, the possibility of bias or omissions cannot be entirely ruled out, and this is acknowledged as a limitation of the study.

### 2.4 Study risk of bias assessment

The ROBINS-I (Risk of Bias in Non-Randomized Studies of Interventions) tool (Sterne et al., 2016) was employed to evaluate the risk of bias (RoB) in the included studies. This tool was selected because most studies in this review employed experimental or observational designs without strict randomization. Given the variability in the conditions assessed, each study was evaluated only for the condition(s) it investigated. For example, studies focusing on mental workload were assessed for bias related to that condition but not for others, such as stress or vigilance.

Studies were rated across the seven ROBINS-I domains, including bias due to confounding, participant selection, and outcome measurement. Ratings included “Low Risk,” “Moderate Risk,” “Serious Risk,” “Critical Risk,” or “No Information,” with the overall RoB determined by the least favorable rating across domains.

RoB assessments were conducted independently by the first author, with discrepancies resolved through discussion or consultation with the other reviewers. Studies lacking sufficient information to assess a domain were rated as “No Information.” While every effort was made to ensure accuracy, the possibility of subjective bias cannot be entirely ruled out. The RoB assessment was considered when interpreting the findings and was used to contextualize the strength of evidence in the narrative synthesis. Particularly, studies rated as having serious or critical risk of bias were not excluded but were interpreted more cautiously, especially regarding the reliability of the reported condition effects impact and classification results.

### 2.5 Effect measures

Data extracted included EEG and ET features, as well as other available measures. Studies varied in the type of information reported: some studies analyzed the variation of collected features with changes in task conditions (e.g., increasing task load for mental workload condition), others provided statistical analyses (such as correlations between features), and others focused on feature selection for classification models (most significant features).

Among the 47 included studies, 29 reported the impact of conditions on EEG and ET features, while the remaining did not explicitly analyze these effects. Additionally, 25 studies reported classification results for identifying conditions based on physiological data. Performance metrics varied across studies, including accuracy (ACC), precision, recall, root mean squared error (RMSE), F1-score, area under the curve (AUC), sensitivity, and specificity.

### 2.6 Synthesis methods

A descriptive synthesis approach was used to summarize and integrate the extracted data. The results were first organized into a comprehensive overview of EEG and ET features across conditions, detailing how frequently each metric was used and its relative prevalence within the literature. A second synthesis qualitatively described how each condition influenced EEG and ET features, emphasizing consistent condition-feature associations and the direction of reported effects. Given the diversity of analytical methods and reporting standards across studies, only qualitative summaries (e.g., reported correlations or directional changes) were included.

Lastly, a classification synthesis table was created for studies that developed classification models, categorizing them by condition, classification type (e.g., binary, multi-class) and reported classification performance. Available performance metrics such as ACC, RMSE, and mean squared error (MSE) were included for EEG-based, ET-based, and multimodal (EEG + ET) approaches. The analyzed data were limited to EEG and ET sensors; therefore, the combination with other types of signals was not considered. Additionally, studies that used the physiological features as classification labels were distinguished. Where applicable, the table also highlighted performance variations across different evaluation settings (e.g., cross-subject, cross-session, gender-group performance). Given the variability in reporting formats, a direct statistical comparison across studies was not conducted; instead, classification performance was qualitatively compared across modalities and evaluation settings.

Since no quantitative analysis was performed, missing data did not require specific handling, and only reported data were included in the synthesis. The choice of descriptive synthesis over meta-analysis was due to the heterogeneity of study designs, extracted features, and reporting formats. Studies were subdivided per condition to facilitate comparisons within each category. No formal sensitivity analyses were conducted.

### 2.7 Reporting bias assessment

Selective reporting bias was evaluated by examining whether studies provided sufficient detail on key results. A summary table was created to systematically document this, indicating whether each study reported the condition's impact on EEG and ET features and/or developed condition classification models.

Concerning publication bias, studies were identified through a comprehensive database search. However, no additional efforts were made to locate unpublished studies or papers outside those databases. Additionally, it is acknowledged that if a study did not explicitly mention both EEG and ET in its abstract, title, or keywords, it may have been mistakenly excluded during initial screening, despite including both modalities in the full text, introducing a potential source of identification bias.

### 2.8 Certainty assessment

Due to the qualitative nature of the synthesis and the variability in targeted conditions and study designs, a formal certainty assessment was not conducted. However, evidence confidence was informally considered based on the overall RoB, the consistency of findings across studies within each condition, and the directness in addressing the research questions. These informal considerations supported identifying general associations while acknowledging limitations in precision and reporting.

## 3 Results

### 3.1 Study selection

**Figure 1:**
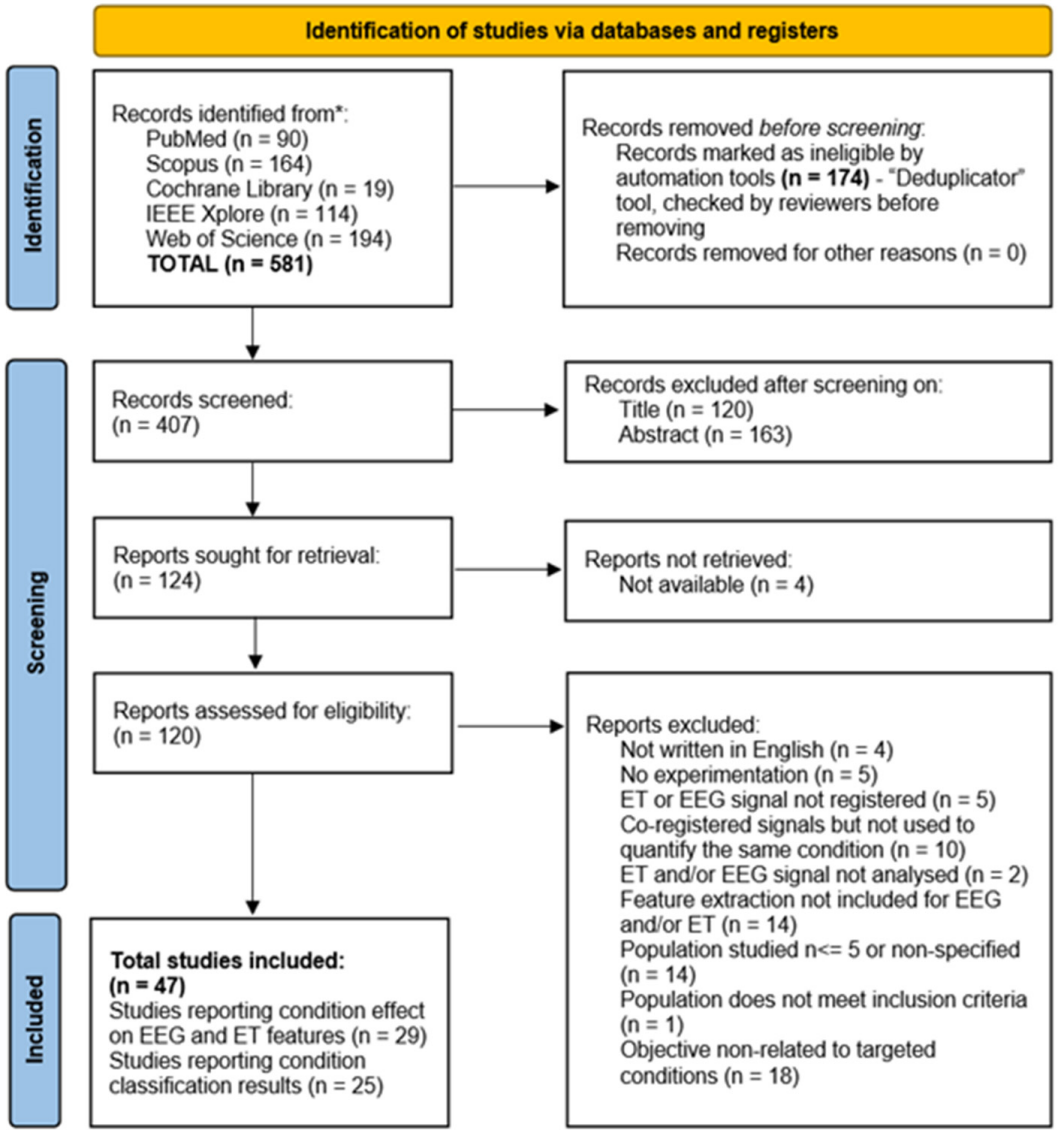
PRISMA 2020 flow diagram.

Initially, 581 studies were identified through database searches. After removing 174 duplicates, title screening excluded 120 studies. Abstract screening was then conducted for the remaining studies, excluding an additional 163 studies. Subsequently, full texts were sought for 124 studies, of which 47 met all eligibility criteria and were included in the final analysis. The study selection process is illustrated in the PRISMA flow diagram (Figure 1).

Among the full-text studies assessed, 77 were excluded based on specific criteria listed in the flow diagram. A notable portion of exclusions corresponded to studies that, while recording both EEG and ET, did not use them to assess the same condition (*n* = 10). For example, some researchers recorded both EEG and ET but only used EEG to assess mental workload, leading to its exclusion (Sengupta K. et al., 2017). Along the same lines, another study measured mental workload via EEG and reaction time via ET without examining their joint relationship under the same conditions (Acerra et al., 2019).

Another group of studies (*n* = 14) was excluded due to a lack of feature extraction for either EEG or ET signals. For instance, in one study, EEG and ET data were recorded during alertness and concentration tasks but did not conduct feature extraction or analysis, resulting in exclusion (Pei et al., 2022). Similarly, (Chatterjee et al., 2021) recorded ET signals alongside EEG signals during attention-related tasks but only analyzed the EEG signals, limiting its applicability for this review.

A further set of studies (*n* = 18) was excluded because their primary objective was not related to attention and vigilance conditions. For example, Kula et al. (2017) evaluated the usability and user experience of different car instrumentation by analyzing visual attention and engagement markers derived from EEG and ET signals.

Finally, additional exclusions comprised studies with fewer than five participants or unspecified sample sizes (*n* = 14); studies with no experimentation (*n* = 5); studies that did not to register either EEG or ET signals (*n* = 5); non-English publications (*n* = 4); studies that did not analyzed either EEG or ET signals (*n* = 2); and populations failing to meet inclusion criteria (*n* = 1).

### 3.2 Study characteristics

#### 3.2.1 Study design

Among the 47 included studies, most followed an experimental approach (*n* = 25), while 20 studies were simulation-based. Additionally, two studies conducted a dataset analysis, where the dataset originated from an experiment using a simulator (Zaky et al., 2021; Zhu, 2021). Regarding control studies, only one reported use of control participants (Bodala et al., 2016).

#### 3.2.2 Population

Across the 47 included studies, the number of participants ranged from 8 to 150, with a mean of 25.48 (SD = 22.95). One study (Matthews et al., 2015) stands out as a superior outlier with 150 participants (based on the mean ± 2^*^SD criterion). Statistically dismissing this study results in a revised range of 8 to 61 participants, with a mean of 22.83 (SD = 14.17). Four outliers remain on the upper end, with seven studies reporting 52, 53, 60, and 61 participant counts. Notably, one study (Planke et al., 2021) conducted two independent studies with sample sizes of 12 and 17, which were treated as separate entries.

As for the population characteristics, six studies focused on specialized populations, including professional drivers (Pan et al., 2024), air traffic controllers (ATCs) (Di Flumeri et al., 2019b), special vehicle crews (Guo et al., 2024), military pilots (Diaz-Piedra et al., 2019; Previc et al., 2009), and marine pilots (Orlandi and Brooks, 2018). All these studies included only male participants, as did five additional studies (Borys et al., 2017a,b; Chua et al., 2012; Di Flumeri et al., 2019a, 2018).

#### 3.2.3 Experimental equipment

ET modalities varied across studies, with 27 using remote systems and 19 using head-mounted systems (one study did not specify ET modality). Sampling rates, when reported (*n* = 38), ranged from 25 to 500 Hz.

EEG systems ranged from single-channel and low-density headbands (*n* = 28) to high-density 124-channel caps (*n* = 18) (one study did not specify EEG system used). Sampling rates, when reported (*n* = 41), ranged from 80 Hz to 10,000 Hz, with most studies (*n* = 30) using recording frequencies between 128 and 512 Hz. The number of channels and targeted regions also varied considerably, with low-density setups (< 32 channels) often targeting prefrontal, frontal, central, or parietal regions, and high-density configurations (32–124 channels) typically covering frontal, central, parietal, temporal, and occipital regions.

Additional details regarding experimental equipment can be found in Supplementary material.

#### 3.2.4 Study classification

There was occasional overlap in the classification of studies based on conditions, particularly in studies investigating fatigue and vigilance with sleep deprivation. However, for clarity, these studies were classified under their primary focus.

The largest category was mental workload, which was the primary focus of 22 studies. Mental workload is a cognitive state reflecting the interaction between task demands and available cognitive resources (Gaillard, 1993). Various methodologies were used to study this condition, such as driving (*n* = 5), various cognitive tasks (*n* = 5), military tasks (*n* = 5), arithmetic tasks (*n* = 3), robotic-assisted surgical tasks (*n* = 2), and other specialized tasks (*n* = 2).

Nine studies examined fatigue, defined as reduced mental or physical performance resulting from sleep disturbances, prolonged cognitive or physical effort or monotonous tasks (ICAO, 2013). Of these, seven induced fatigue through cognitive demands, while two studies focused on fatigue caused by sleep deprivation (Li et al., 2023; Previc et al., 2009). In addition, many studies (*n* = 4) focused on studying this condition during a driving task.

Seven studies focused on vigilance, sustained attention over time (Oken et al., 2006). One of them specifically examined vigilance during sleep deprivation (Chua et al., 2012). At a lower level of arousal, six studies assessed drowsiness, the transitional state from wakefulness to sleep (Chowdhury et al., 2018), and sleep-related conditions. For example, four studies included the investigation of micro-sleeps (Arsen'ev et al., 2015; Poudel et al., 2010; Zaky et al., 2021, 2023), which are involuntary lapses of sleep over a few seconds (Gradwell and Wilkinson, 2025).

Three studies explored mind-wandering, an involuntary shift of attention away from the primary task toward unrelated thoughts during periods of monotony or boredom (Smallwood and Schooler, 2015). Finally, two studies examined stress, a state that emerges when intentions cannot be realized or when task and environmental demands are perceived as uncontrollable (Gaillard, 1993), together with fatigue (Gündoǧdu et al., 2019, 2021).

Table 1 provides a detailed breakdown of the studies, including study design, sample size, conditions, and tasks. Additionally, it includes a summary of whether the study assessed the condition's impact on the measured features and/or classification of the studied conditions.

**Table 1:** Characteristics of the included studies.

**Study**	**Study design**	**Sample size**	**Condition(s) & tasks**	**Impact on feature**	**Classification**
(Gündoǧdu et al. 2021)	Experimental	10	Stress, mental fatigue, and attention measures during e-sport activity (test battery: VAS, d2 test, N-back test, electronic sport game task)	Yes	No
(Gündoǧdu et al. 2019)	Experimental	8	Stress and mental fatigue during 3 N-back test tasks (Position single task, Position-Color 2-Back task and Position Image 2-Back task)	Yes	No
(Hopstaken et al. 2016)	Experimental	47	Mental fatigue during a cognitive task: time-on-task paradigm (N-back task, visual letter 2-back task) with alternative task-unrelated stimuli (reward stimuli) to examine attentional disengagement	Yes	No
(Previc et al. 2009)	Simulator	10	Fatigue during sleep deprivation	Yes	No
(Li et al. 2023)	Experimental	35	Fatigue based on KSS drowsiness level (test battery: PVT, KDT, AAT, V-P300, A-P300, VAS, KSS-CN)	Yes	Yes
(Huo et al. 2016)	Simulator	21	Fatigue during driving	No	Yes
(Zhu 2021)	Dataset analysis	15	Fatigue during driving	No	Yes
(He et al. 2016)	Simulator	50	Fatigue during driving	No	Yes
(Zhang et al. 2023)	Simulator	8	Fatigue during driving	No	Yes
(Esposito et al. 2022)	Experimental	15	Attention, boredom and mind wandering during vigilance and sustained attention test: MCT (adapted for boredom and mind wandering inclusion)	Yes	No
(Asish et al. 2024)	Experimental	27	Internal and external distractions in educational VR environments	No	Yes
(Reßing et al. 2022)	Experimental	45	Mind wandering while using digital technologies during divergent thinking tasks: UTT	No	No
(Pan et al. 2024)	Simulator	60	Vigilance during monotonous driving task (dual-task paradigm: simulated driving as primary task, random stimulus detection as secondary task)	No	Yes
(Larue et al. 2015)	Simulator	25	Alertness during simulated highway-driving task	No	Yes
Sengupta A. et al. (2017)	Experimental	30	Alertness during mental workload (test battery: ST, VRT, LC, ART)	Yes	No
(Farha et al. 2022)	Experimental	9	Vigilance during modified version of the Stroop color word task (SCWT)	Yes	Yes
(Di Flumeri et al. 2019b)	Simulator	14	Vigilance of ATCos using a highly automated HMI	Yes	No
(Bodala et al. 2016)	Experimental	12	Vigilance during challenge integration: monitoring task as primary task and noisy visual stimulus as challenging stimulus	Yes	No
(Chua et al. 2012)	Experimental	24	Sleep decrements in psychomotor vigilance during sleep deprivation: PVT task	Yes	Yes
(Zaky et al. 2023)	Experimental	20	Microsleeps during 2D CVT task	Yes	No
(Zaky et al. 2021)	Dataset study	14	Microsleeps during 2D CVT task	Yes	No
(Poudel et al. 2012)	Experimental	20	Drowsiness during CVT task	No	No
(Poudel et al. 2010)	Experimental	20	Tonic drowsiness and microsleeps during 2D pursuit-tracking task	No	No
(Arsen'ev et al. 2015)	Experimental	19	Drowsiness (decrease in level of arousal) and microsleeps during visuomotor coordination task	Yes	No
(Zandi et al. 2019)	Simulator	53	Drowsiness during driving	No	Yes
(Borys et al. 2017b)	Experimental	20	Mental workload during arithmetic tasks with different levels of difficulty	No	No
(Borys et al. 2017a)	Experimental	20	Mental workload during arithmetic tasks	No	Yes
(Kujur et al. 2022)	Experimental	13	Mental workload during numerical estimation tasks, with induced distraction	Yes	Yes
(Guo et al. 2024)	Simulator	20	Mental workload during a multi-phase operational task (search, strike, observe, report)	Yes	Yes
(Singh et al. 2021)	Simulator	14	Mental workload during MUM-T for Pilot-UAV Teaming Applications	Yes	Yes
(Diaz-Piedra et al. 2019)	Simulator	15	Mental workload during in-flight emergencies (task load variations as a function of flight complexity)	Yes	No
(Matthews et al. 2015)	Simulator	150	Mental workload during 4 military monitoring tasks (simulation of unmanned ground vehicle operation)	No	No
(Di Flumeri et al. 2019a)	Experimental	8	Mental workload during driving	Yes	No
(Di Flumeri et al. 2018)	Experimental	20	Mental workload during driving (different traffic conditions and road types)	Yes	No
(Yang et al. 2020)	Simulator	32	Mental workload and behavior during driving	Yes	No
(Angkan et al. 2024)	Simulator	23	Mental workload during driving	No	Yes
(Eniyandunmo et al. 2024)	Simulator	52	Mental workload during driving (five-task driving scenario)	Yes	Yes
(Shafiei et al. 2024)	Simulator	26	Mental workload during surgical tasks with different difficulty levels	No	Yes
(Barragan et al. 2022)	Simulator	8	Mental workload during robotic-assisted surgical tasks	Yes	Yes
(Orlandi and Brooks 2018)	Simulator	10	Mental workload during different ship-handling conditions while berthing ships in a simulator	Yes	No
(Iqbal et al. 2024)	Experimental	8	Mental workload of control room operators	No	Yes
(Jimenez-Molina et al. 2018)	Experimental	61	Mental workload during web browsing task	Yes	Yes
(Lobo et al. 2016)	Experimental	21	Mental workload during dual task paradigm: primary task (visual search task), secondary interfering task (syntactic transformation task).	No	Yes
(Mark et al. 2024)	Simulator	23	Mental workload during six cognitive tasks (working memory, vigilance, risk assessment, shifting attention, situation awareness, and inhibitory control)	Yes	No
(Aksu et al. 2024)	Simulator	15	Mental workload during n-back tasks	Yes	Yes
(Planke et al. 2021)	Experimental	17	Mental workload during MATB scenario	Yes	Yes
(John et al. 2022)	Experimental	24	Mental workload during tacking and collision prediction tasks with 3 levels of difficulty	Yes	Yes

### 3.3 Risk of bias in studies

Regarding RoB, most of the included studies (*n* = 34) were categorized as moderate overall RoB, while 10 studies were rated as low and three as serious.

Confounding (D1), participant selection (D2), and missing data (D5) were notable sources of potential bias, primarily due to the high proportion of studies with insufficient reporting in these domains. For D1, while 20 studies had low risk, 25 did not provide sufficient information, and two were moderate. Similarly, in D2, 17 studies had low risk, nine moderate risk, and 21 lacked adequate information. In D5 20 studies were at low risk but 27 lacked information. Missing or unclear reporting in D1 and D2 could suggest a potential problem when comparing different studies, while in D5 could introduce uncertainty if data loss was non-random. In contrast, for selection of reported results (D7), 22 studies had low risk, 21 had moderate risk, three were categorized as serious, and one did not report information. Partial reporting could limit the discovery of associations between studies. Lastly, classification of interventions (D3), deviations from intended interventions (D4), and outcome measurement (D6) generally showed low risk (42, 36, and 41 studies respectively), indicating that interventions were mostly well-defined, appropriately implemented, and reliably measured to allow comparability across studies (Jüni et al., 2016; Sterne et al., 2016).

A visualization plot of the complete RoB of the included studies broken down for each criterion can be found in the Supplementary material [obtained using robvis tool (McGuinness and Higgins, 2020)].

### 3.4 Results of synthesis

Across all conditions, EEG power bands were the most consistently analyzed feature. From a neuroscientific perspective, the commonly studied bands include delta (1–4 Hz), theta (4–8 Hz), alpha (8–12 Hz), beta (12–30 Hz), and gamma (30–150 Hz). These frequency bands are broadly associated with distinct cognitive signatures: delta with target detection and inhibitory control, theta with memory and executive processes, alpha with attentional modulation and cortical inhibition, beta with motor and sensorimotor functions, and gamma with cortical activation and perceptual processing. However, these are general functional associations rather than direct links to specific cognitive functions; thus, increases or decreases in power can reflect different cognitive demands depending on the context (Herrmann et al., 2016).

In contrast, ET metrics showed greater variability, although certain features appeared with higher frequency. ET metrics can be broadly grouped into gaze behavior, eyelid opening, and pupil-based measures. Within gaze behavior, fixations are events in which the eyes remain on a location to process visual information, while saccades are the rapid transitions between fixation points that allow for visual exploration. Eyelid opening-related metrics include blinks, semi-voluntary brief closures of the eyelids, and eyelid openness or percentage of eyes closed (PERCLOS), which quantify the time and percentage the eyes are closed, are commonly used as indicators of cognitive processes such as visual attention or arousal. Lastly, pupil-based measures include pupil diameter (absolute size of the pupil) and pupil dilation (change in pupil size over time) and reflect changes in cognitive load and arousal, although are highly sensitive to changes in brightness (Klein and Ettinger, 2019; Skaramagkas et al., 2023).

Across the selected studies, pupil diameter and fixation metrics were the most common (both *n* = 20), followed by blinks and PERCLOS/eye-closure metrics (both *n* = 17). At the condition level, the most frequently used ET features were gaze heatmaps for stress (*n* = 2), while PERCLOS was predominant in fatigue studies (*n* = 5). Mind-wandering studies most often employed gaze metrics (*n* = 2) and pupil diameter (*n* = 2). For vigilance, blinks (*n* = 5), saccades (*n* = 4), fixations (*n* = 4), and eye closure/PERCLOS (*n* = 4) were the most commonly analyzed. Drowsiness and sleep studies mainly used gaze metrics (*n* = 6), eyelid closure/PERCLOS (*n* = 5), and blinks (*n* = 3). Lastly, for mental workload, pupil diameter and fixation-related metrics were the most frequently used (both *n* = 13).

A summary of the extracted metrics per condition is provided in the Supplementary material.

#### 3.4.1 Impact of condition on EEG and ET features

Grouping studies by condition enabled the characterization of EEG and ET features associated with each condition (Table 2).

**Table 2:** Summary of EEG and ET metrics changes across conditions.

**Metrics**	**Stress**	**Fatigue**	**Mind wandering**	**Vigilance (decrement)**	**Drowsiness and sleep**	**Mental workload**
**Eye gaze**						
Gaze metrics	-	↑ missing gaze	-	-	↓ focus, ↑ variability from target center (drowsiness), flat or incoherent gaze for 0.05–0.15 s (microsleeps)	-
Fixation metrics	-	-	↑	↑ TTFF, ↑ fixations/s	-	↑ rate, ↓ or ↑ duration, ↓% external environment
Saccade metrics	-	-	-	↓ velocity, ↓ amplitude	↑ latent periods (drowsiness), absent (microsleeps)	↑ number, ↑ amplitude, ↑ duration
Scan & Pattern-based metrics	↑ heatmap dispersion	↑ heatmap dispersion (stable^*^)	-	-	-	↑ gaze entropy, (↓ gaze entropy ^*^)
**Eyelid opening**						
Eyelid Openness – Closure/PERCLOS	-	↑	-	↑	>80% (microsleeps)	-
Blinks	-	-	-	↑ rate (↓ rate ^**^)	-	↑ duration, ↑ or ↓ rate
**Pupil dilation**						
Pupil diameter/size	**-**	**↓**	**-**	**-**	**-**	**↑↑**
Pupil dilation	**-**	**-**	**-**	**-**	**-**	**↑**
**EEG frequency bands**						
δ band (1–4 Hz)	-	↑^**^	-	↑, ↓ after 22–26 hrs^**^	↑↑ (microsleeps), ↓ (after microsleeps)	↑or ↓ (region changes)
θ band (4–8 Hz)	↑	↑	-	↑^**^	↑ (drowsiness), ↑↑ (microsleeps)	↑
α band (8–13 Hz)	↑	↑ (↓^**^)	↑	↑ (↓^**^)	↑ (drowsiness), ↑ (microsleeps), ↓ (after microsleeps)	↓ or ↑ (region changes)
β band (13–30 Hz)	↑	↑	-	↑ post-sleep hrs^**^	↑ (microsleeps)	↑or ↓ (region changes)
γ band (30–100 Hz)	-	-	-	-	↑ (microsleeps)	↑
Power ratios	↓α/β	↓α/β	-	↓θ/α, ↑(α+θ)/β	↓β/α (drowsiness), ↓α/(δ+θ) (drowsiness)	↑θ/α, ↑β/(θ+α)
**Time-based**						
P3 amplitude	**-**	**↓**	**-**	**-**	**-**	**-**

Reported effects of conditions on EEG and ET features. For each condition, feature variations (e.g., increased/decreased values) are reported alongside the direction of observed associations when available.

Standardized symbols (↑, ↓, ↑↑) indicate increases, decreases, or strong variations, facilitating comparisons across conditions.

hrs, hours; PERCLOS, percentage of eyes closed, TTFF: time-to-first-fixation.

^*^Association observed in professional participants, reflecting practiced scanning behavior.

^**^Sleep deprivation effects on feature associations.

Under stress, gaze heatmaps showed an increased pattern dispersion, suggesting less focused attention (Gündoǧdu et al., 2019, 2021). Similarly, EEG power analysis revealed higher alpha, beta, and theta power, while the alpha/beta ratio decreased, indicating a relatively more significant increase in beta activity than alpha activity (Gündoǧdu et al., 2021).

Fatigue similarly led to increased gaze heatmap dispersion (Gündoǧdu et al., 2019, 2021), in addition to more offscreen/missing gaze, reduced pupil diameter (Hopstaken et al., 2016), and increased PERCLOS (Li et al., 2023). However, scanning behavior remained largely unaffected among professionals (USAF pilots), suggesting that well-rehearsed scanning strategies may be resistant to fatigue, particularly in expert populations (Previc et al., 2009). As for EEG, with fatigue, alpha, beta, and theta power increased and the alpha/beta ratio (Gündoǧdu et al., 2019, 2021) and P3b amplitude decreased (Hopstaken et al., 2016). However, with sleep deprivation, there was an increase in delta and theta power but a decrease in alpha with wakefulness, showing an opposite pattern of associations between EEG features and the condition (Li et al., 2023; Previc et al., 2009).

Mind wandering was linked to increased alpha activity (Esposito et al., 2022). Furthermore, the onset of alpha-band event-related desynchronization (ERD) occurred immediately upon gaze fixation, suggesting active visual processing during mind wandering episodes (Reßing et al., 2022).

Vigilance decrement was characterized by increased PERCLOS, blink frequency (Bodala et al., 2016), fixations per second, and TTFF (Di Flumeri et al., 2019b), alongside a reduction in saccadic velocity and amplitude (Bodala et al., 2016; Farha et al., 2022; Sengupta A. et al., 2017). EEG changes included increased alpha and delta activity (Bodala et al., 2016; Sengupta A. et al., 2017), higher (alpha + theta)/beta ratio (Larue et al., 2015), and lower theta/alpha ratio (Bodala et al., 2016). The impact of sleep deprivation on vigilance led to a decreased number of blinks, increased PERCLOS and theta and beta activity, and decreased alpha (during usual sleep hours) and delta (after 22–24 h) (Chua et al., 2012).

Drowsiness was characterized by a less focused gaze with increasing variability and increased latent periods of saccades, while saccadic movement was absent for microsleeps (Arsen'ev et al., 2015; Zandi et al., 2019). Moreover, overall, microsleeps were defined based on flat or incoherent gaze tracking (0.5–0.15 s) accompanied by complete or partial eye closure (over 80% PERCLOS). EEG patterns showed increased theta and alpha during drowsiness, with microsleeps exhibiting heightened beta, gamma, alpha, delta, and theta activity, followed by a decrease in alpha and delta upon microsleep termination (Arsen'ev et al., 2015; Poudel et al., 2010, 2012; Zaky et al., 2021, 2023). Additionally, reductions in beta/alpha and alpha/(delta+theta) ratios signaled drowsiness (Zandi et al., 2019).

Mental workload generally was indicated by increased beta, theta, gamma, and delta activity (Eniyandunmo et al., 2024; Guo et al., 2024; Jimenez-Molina et al., 2018; Kujur et al., 2022; Orlandi and Brooks, 2018; Singh et al., 2021), though regional differences in beta and delta were noted (Barragan et al., 2022; Diaz-Piedra et al., 2019; John et al., 2022). Similarly, alpha power typically decreased (Jimenez-Molina et al., 2018; John et al., 2022; Singh et al., 2021; Yang et al., 2020) but was sometimes reported to increase depending on brain regions (Aksu et al., 2024). Additionally, there was also an increase in the theta/alpha ratio (Di Flumeri et al., 2018) as well as in the engagement index [beta/(theta+alpha)] (Singh et al., 2021). ET changes included more significant pupil dilation and size, saccade number, amplitude and duration, fixation rate and blink duration (Aksu et al., 2024; Borys et al., 2017b; Eniyandunmo et al., 2024; Guo et al., 2024; Jimenez-Molina et al., 2018; John et al., 2022; Kujur et al., 2022; Mark et al., 2024; Orlandi and Brooks, 2018; Planke et al., 2021; Singh et al., 2021). Dwell time and scan pattern entropy also increased (Planke et al., 2021), although professionals (military fighter pilots) displayed more systematic scanning patterns with lower entropy (Orlandi and Brooks, 2018). This finding aligns with standardized aviation procedures, where pilots rely on deterministic scanning behavior during specific scenarios. On the other hand, fixations on task-unrelated regions, fixation duration, and blink rate decreased (Borys et al., 2017b; Di Flumeri et al., 2019a, 2018; John et al., 2022; Planke et al., 2021; Singh et al., 2021; Yang et al., 2020). However, some variations were reported regarding fixation duration and blink rate, such as increased fixation duration (Yang et al., 2020) and blink number (Aksu et al., 2024), which could be due to differences in the type of task used to induce mental workload. A summary of metric changes across conditions is provided in the Supplementary material.

Taken together, several EEG and ET features showed recurring patterns across conditions, while others diverged. In EEG, increased theta activity was reported in stress, fatigue, vigilance decrement, drowsiness, and mental workload studies. Beta power also tended to increase, although some mental-workload studies reported regional decreases, indicating task and region-specific variability. Alpha activity generally increased across conditions but decreased under some instances of high mental workload, sleep deprivation or immediately after microsleeps, reflecting opposite associations depending on the underlying cognitive demand. Delta activity showed no reliable cross-condition pattern, and gamma activity was rarely examined, with increases documented only for microsleeps and mental workload.

As for ET metrics, increased scanpath dispersion was observed in stress, fatigue, and mental workload studies, as well as this effect diminishing when participants were highly trained professionals, suggesting expertise-related stability. Other eye gaze metrics such as increased fixations were seen in mind wandering, vigilance decrement and mental workload studies, whereas saccade amplitude diverged, decreasing during vigilance decrement but increasing under mental workload. For eyelid opening metrics, PERCLOS was shown to increase for fatigue, vigilance decrement and drowsiness studies, while blink-related metrics varied across studies, particularly for vigilance decrement and mental workload. Lastly, for pupil dilation metrics, pupil diameter decreased with fatigue but increased with mental workload.

Despite these patterns, comparisons across conditions were often limited by heterogeneous feature extraction, as many studies used only a subset of EEG or ET metrics.

#### 3.4.2 Classification of conditions based on EEG and ET features

Of the studies included in this review, 25 investigated the classification of the respective elicited conditions. Various classification strategies were employed, including binary classification (*n* = 12), multi-class classification (*n* = 8 for three classes and *n* = 6 for four classes), and continuous classification (*n* = 3). Among conditions with more than one classification study (fatigue, vigilance, and mental workload), binary and multi-class approaches were relatively balanced, while continuous classification was less common. Mind wandering and drowsiness/sleep were exclusively studied using binary classification, and no classification studies were found for stress.

Seven studies adopted the approach of using one signal to label the condition while another was used for classification (four using EEG and three using ET as labels). Fatigue studies predominantly employed this method (*n* = 4), utilizing both EEG and ET as condition labels. Similarly, one vigilance and one drowsiness/sleep study used EEG as a label, while one mental workload study used ET. Beyond studies that used one modality to label the other, a wide range of additional labeling strategies was employed: six studies used task-level labels, where predefined task difficulty levels where treated as ground truth, six studies relied on subjective ratings, five studies used performance-based metrics, and one studied compared the use of subjective and performance-based labeling methods (Guo et al., 2024).

Regarding validation approaches, the most common strategy was k-fold cross-validation, typically 5-fold or 10-fold, sometimes stratified to balance class distributions. Additionally, other studies implemented train-test splits, rolling-origin time series validation, or leave-one-subject-out (LOSO). A minority combined multiple strategies depending on the design, such as intra-subject tuning with inter-subject validation or mixed approaches for ecological vs. traditional experimental setups (Angkan et al., 2024; Asish et al., 2024; Singh et al., 2021). Overall, this heterogeneity in validation approaches reflects the diverse goals of studies, from optimizing model performance to evaluating generalizability, with the latter remaining largely untested.

Concerning the impact of signal fusion on classification, only nine of the studies reported results of models using individual sensors along with their combination. Five studies presented only fusion results, two compared EEG and fusion, and two compared EEG and ET. Additionally, one study reported only results for ET without using EEG as a label. Across studies, multimodal fusion generally improved classification performance for mind wandering, vigilance, and mental workload, typically outperforming EEG-only or ET-only models. ET features tended to outperform EEG in mind wandering and mental workload, whereas EEG was superior for vigilance. Comparative data for fatigue and drowsiness were not available.

Classification performance varied by condition (see Table 3). For fatigue, using either EEG or ET as a label showed comparable classification performance (He et al., 2016; Huo et al., 2016; Zhang et al., 2023; Zhu, 2021). When comparing the results of those studies to a fatigue classification model built with both sensors (Li et al., 2023), performance was lower. This comparison is limited, however, as the fusion study addressed a four-class problem and did not report on individual sensor classification. In mind wandering classification, sensor fusion consistently improved classification performance across different scenarios (cross-subject and gender-grouping), achieving 83% and 87% accuracy, respectively, and with single-ET outperforming single-EEG classification (Asish et al., 2024). For vigilance classification, one study employed EEG as the label for three-level classification, achieving over 90% accuracy (Larue et al., 2015). Another study classified four vigilance classes, with ET-only reaching 75% accuracy, EEG-only 83%, and EEG+ET fusion 88% (Pan et al., 2024). Likewise, for a binary classification, researchers found that fusion enhanced performance (76% for ET, 92% for EEG, and 97% for fusion) (Farha et al., 2022). One more study also reported strong binary classification results for EEG and ET but did not include a fusion comparison using both sensors (Chua et al., 2012). For drowsiness/sleep classification, fusion's impact could not be assessed as only EEG was used as a label, though ET alone achieved strong results (88% accuracy) (Zandi et al., 2019).

**Table 3:** Summary of performance of EEG-only, ET-only, and EEG+ET fusion methods across conditions.

**Condition**	**Study**	**Classes**	**Best classifier**	**EEG-only**	**ET-only**	**Fusion EEG+ET**
Fatigue	(Li et al. 2023)	4	LR	*Not reported*	*Not reported*	ACC: 76.8%, P: 62.9%, R: 75.4%
	(Huo et al. 2016)	-	DG-RELM	RMSE: 0.1037	**Label**	
	(Zhu 2021)	2	NN	**Label**	MAE: 1.361e-6	
	(He et al. 2016)	2	ANN	**Label**	ACC: 89.2%−88.9%	
	(Zhang et al. 2023)	2	LSTM	ACC: 93.1%	**Label**	
Mind wandering	(Asish et al. 2024)	2	RF	ACC: 69.9%; 69%−74% (by gender)	ACC: 77.3%; 79%−82% (gender)	ACC: 83.60%; 87%−91% (gender)
Vigilance	(Pan et al. 2024)	4	WDCGAN + CNN + LSTM	ACC: 83.0%, F1: 83%	ACC: 75.75%, F1: 76%	ACC: 88.75%, F1: 89%
	(Larue et al. 2015)	4	NN	**Label**	ACC: 91%; AUC: > 0.88	
	(Farha et al. 2022)	2	SVM	ACC: 92%, S: 91.7%, Sp: 92.2%	ACC: 76.8%, S: 76.4%, Sp: 77.1%	ACC: 96.8%, S: 97.2%, Sp: 96.4%
	(Chua et al. 2012)	2	THR	AUC: 0.82, S: 70.8%−77.8%, Sp: 76.3%−83.8%	AUC: 0.89–0.91, S: 76.9%−81.3%, Sp: 87.3%−87.6%	*Not reported*
Drowsiness and sleep	(Zandi et al. 2019)	2	RF	**Label**	ACC: 88.37%−91.18%	
Mental workload	(Borys et al. 2017a)	2, 3	BT, kNN, SVMs ^*^	ACC: 73.9%, AUC: 0.66 (binary); ACC: 50.4% (multi)	ACC: 90.4%, AUC: 0.9 (binary); ACC: 73% (multi)	ACC: 85.2%, AUC: 0.93 (binary); ACC: 63.5% (multi)
	(Kujur et al. 2022)	2	XGB	*Not reported*	*Not reported*	ACC: 66%
	(Guo et al. 2024)	3	LDA	*Not reported*	*Not reported*	ACC: 84.3%
	(Singh et al. 2021)	3	LDA	ACC: 67% (intra-subject); ACC: 55.6% (inter-subject)	ACC: 56% (intra-subject); ACC: 55% (inter-subject)	ACC: 66% (intra-subject); ACC: 55.6% (inter-subject)
	(Angkan et al. 2024)	2, 3	RF, XGB ^*^	ACC: 77.4%, F1: 73.4% (binary); ACC: 64.5%, F1: 64.1% (3-class)	*Not reported*	ACC: 80.8%, F1: 78.1% (binary); ACC: 71.2%, F1: 71.2% (3-class)
	(Eniyandunmo et al. 2024)	4	LinR	ACC: 88 ± 5%, F1: 89.5 ± 14.5%	ACC: 88 ± 5%, F1: 89.5 ± 14.5%	ACC: 88 ± 5%, F1: 89.5 ± 14.5%
	(Shafiei et al. 2024)	-	XGB	R^2^: 0.75–0.9, MAE: 6.6–14.1, RMSE: 9.2–18	R^2^: 0.64–0.75, MAE: 6.5–13.5, RMSE: 8.7–18.05	R^2^: 0.81–0.83, MAE: 4.5–11, RMSE: 6.8–13.8
	(Barragan et al. 2022)	2	LSTM, NN, RF ^*^	ACC: 68.3%−77.9%	*Not reported*	ACC: 78.6%−80.9%
	(Iqbal et al. 2024)	3	DT	ACC: 50.2%	ACC: 63.07%	ACC: 66.8%
	(Jimenez-Molina et al. 2018)	4	MLP	ACC: 70.9%, R: 82%, P: 65.1%, K: 58.4%	**Label**	
	(Lobo et al. 2016)	3	kNN	*Not reported*	*Not reported*	P: 15.7%, R: 16.5%, F1: 15.5% (Low); P: 69.6%, R: 68.8%, F1: 68.9% (M); P: 16%, R: 15.7%, F1: 15.6% (High);
	(Aksu et al. 2024)	2, 3, 4	Light GBM	ACC: 56.15% (4-class)	ACC: 65.67% (4-class)	ACC: 71.9%, K: 63%, AUC: 0.92 (4-class); ACC: 80.5%, K > 70% (3-class); ACC: 89.6%, K > 70% (2-class)
	(Planke et al. 2021)	4	ANFIS	*Not reported*	MAE: 0.51	*Not reported*
	(John et al. 2022)	-	Multiple LinR	*Not reported*	*Not reported*	R^2^: 54.3%−61.7%

The classification approaches include binary, multi-class, and continuous models. If data was not reported for a specific modality, it is marked as Not reported. If a signal was used as a classification label, the respective signal column is marked as Label and the Fusion EEG+ET column is shaded in dark gray.

^*^best classifier depended on signal and number of classes.

The classifiers include LR, Logistic Regression; DG-RELM, Discriminative Graph Regularized Extreme Learning Machine; NN, Neural Network; ANN, Artificial Neural Network; LSTM, Long Short-Term Memory Network; RF, Random Forest; WDCGAN, Wasserstein Deep Convolutional Generative Adversarial Network; CNN, Convolutional Neural Network; SVM, Support Vector Machine; THR, Threshold-based supervised learning; BT, Bagged trees; kNN, k-Nearest Neighbors; XGB, Extreme Gradient Boosting; LDA, Linear Discriminant Analysis; LinR, Linear Regression; DT, Decision Tree; MLP, Multi-Layer Perceptron; GBM, Gradient Boosting Machine; ANFIS, Adaptive Neuro Fuzzy Inference System.

The reported performance metrics include ACC, accuracy; AUC, area under the curve; P, precision; R, recall; F1, F1-score; K, Kappa; S, sensitivity; Sp, specificity; R^2^, coefficient of determination; MAE, mean absolute error; MSE, mean squared error; RMSE, root mean squared error.

Lastly, in mental workload classification, four studies focused only on fusion models (Guo et al., 2024; John et al., 2022; Kujur et al., 2022; Lobo et al., 2016), while one reported only ET results (Planke et al., 2021). Additionally, one study employed ET as a label to classify four workload levels, achieving 70% accuracy (Jimenez-Molina et al., 2018). In binary mental workload classification, one study found that fusion improved accuracy (78%) compared to an EEG-only model (68%) (Barragan et al., 2022). Similarly, another study reported superior fusion performance to EEG in both binary (80% vs. 77%) and three-class classification (71% vs. 64%) (Angkan et al., 2024). For a three-level mental workload classification, researchers found that fusion achieved the highest accuracy (66%), followed by ET (63%) and EEG (50%) (Iqbal et al., 2024). Another study also showed that fusion outperformed ET and EEG in four-level classification (71% vs. 65% vs. 56%) (Aksu et al., 2024). In a continuous classification, fusion also resulted in higher coefficient of determination (R^2^) and lower mean absolute error (MAE) and RMSE than individual signals (Shafiei et al., 2024). However, one study reported no significant difference between fusion and individual sensors (Eniyandunmo et al., 2024) and two studies found individual sensors outperforming fusion: one of them reported higher EEG accuracy (67%) than fusion (66%) in three-level classification (Singh et al., 2021), and the other found ET (90%) superior to fusion (85%) (Borys et al., 2017a).

### 3.5 Reporting biases

There was notable variability in the type of results reported across studies. As summarized in Table 1, out of the 47 included studies, 12 studies (25.5%) reported the condition's impact on EEG and ET features along with classification outcomes. Another 12 studies (25.5%) examined only condition's impact on EEG and ET features without including classification results, while eight studies (17.0%) focused exclusively on classification outcomes. Additionally, five studies (10.6%) did not report either type of analysis. Overall, this variability reflects differences in study objectives rather than a selective omission of results. However, the lack of specific analyses in some studies limited the possibility of a comprehensive comparison of findings.

### 3.6 Certainty of evidence

Findings were generally consistent across studies of the same condition, particularly regarding commonly reported EEG and ET features. However, some variability was noted in specific metrics, which may reflect methodological differences or population characteristics.

The included studies predominantly provided direct evidence, with EEG and ET features assessed concerning the impact of the target conditions. Nevertheless, the moderate risk of bias observed in many studies and the limited statistical certainty reporting suggest a cautious interpretation of the observed condition-feature associations.

## 4 Discussion

This systematic review was grounded in the cognitive model of perception developed in a previous study, which characterizes how different cognitive states, such as mental workload, stress, fatigue, and drowsiness, can influence and interact to affect human perception in operational environments (Córdoba et al., 2024). The model emphasizes that these conditions often co-occur or unfold sequentially in real-world contexts, highlighting the importance of distinguishing between them for accurate monitoring. In this context, our review aimed to examine how multimodal recordings of EEG and ET have been employed to assess these perception-related conditions and evaluate the potential of these modalities for simultaneous monitoring.

Across the 47 studies reviewed, several recurring condition-feature associations were observed. The most frequently employed EEG measures were frequency bands metrics, while common ET indicators included fixation-related metrics, pupil diameter, blink-related metrics, and PERCLOS. However, several potentially informative metrics have not been utilized in co-registration studies, despite their established relevance in single-modality studies. For example, pupil size has been linked to stress, and saccadic movement features are frequently used in fatigue detection (Bafna and Hansen, 2021; Skaramagkas et al., 2023).

The combined impact of EEG and ET features varied across the conditions reviewed, with some common indicators observed in multiple conditions, whereas other metrics showed condition-specific relationships (Table 2). Mental workload studies commonly reported elevated theta activity and enlarged pupil diameter, indicators known to rise with increasing task difficulty (Lean and Shan, 2012; Tolvanen et al., 2022). However, findings across other EEG bands (e.g., delta, alpha, and beta) and ET features (e.g., fixation duration, blink rate, and saccade amplitude) demonstrated inconsistent relationships across studies.

Stress studies showed elevated theta and beta power, aligning with prior evidence that links increases in the theta band to emotional stress and cognitive effort and in the beta band to anxiety (Nirabi et al., 2022). However, an unexpected increase in alpha activity was reported, contradicting the typical alpha decrease seen during stress. This discrepancy may be explained by the included studies investigating stress and fatigue together, making it difficult to distinguish their unique neural signatures.

Fatigue-related studies revealed increasing EEG activity across the delta, theta, alpha, and beta bands. While these associations reliably reflected fatigue onset, particularly the increases in theta and alpha power, delta and beta bands changes during fatigue have shown inconsistent relationships, hypothesized to be due to differences in fatigue-inducing tasks (Tran et al., 2020). Additionally, decreased pupil diameter and increased PERCLOS have been widely used in fatigue detection (Bafna and Hansen, 2021).

Both vigilance decrement and drowsiness conditions can be considered part of a continuum. Early signs of vigilance decrement are reflected in increased delta and theta power in EEG, alongside subtle changes in ET metrics such as decreased saccadic velocity and amplitude and rising PERCLOS values. These markers signal an initial cognitive slowing and lapses in sustained attention. As drowsiness deepens, these changes intensify: theta and alpha power rise, fixations become less coherent, and the blink rate increases. Once PERCLOS exceeds approximately 80%, microsleep episodes can occur, marked by pronounced increases in delta activity, absent saccades, and flat or incoherent gaze patterns. Some other findings, such as high-frequency EEG components during microsleeps, have unclear functional significance and lack consistent replication.

Lastly, although fewer studies targeted mind wandering, both increased alpha power and increased fixations without processing were reported, reflecting a scanning behavior and EEG patterns consistent with previous research (Kam et al., 2022; Reichle et al., 2010; Schad et al., 2012).

When synthesizing results across conditions, increased theta and beta power were the most consistently reported EEG features, whereas alpha and delta activity showed more divergent patterns. Regional variations, particularly in mental-workload studies, further suggest sensitivity to task- and region-specific dynamics. ET indicators exhibited similar shared and condition-specific relationships: increased scanpath dispersion, fixations, and PERCLOS appeared across multiple conditions, while metrics such as pupil diameter, saccade amplitude, and blink behavior varied depending on condition and task structure.

Together, these multimodal patterns highlight that EEG and ET often provide complementary information, as features that are uninformative in one modality for a given condition may be discriminative in the other. This is particularly relevant for those conditions that often co-occur, such as mental workload and stress leading to fatigue, as conceptualized in our perception framework. Consistent with this complementarity, combining both sensing modalities for classification tasks has generally shown improved performance compared to using either signal alone (Table 3). As a result, these findings emphasize the value of multimodal measurement for differentiating between closely related cognitive states and, ultimately, for building monitoring systems capable of detecting perception deterioration in real-time.

### 4.1 Strengths and contributions

To our knowledge, this review represents the first systematic effort to examine EEG and ET together across a broad range of perception-related conditions. By focusing on concurrent recordings, it provides a comprehensive overview of how neural and ocular markers jointly reflect fluctuations in attention, vigilance, and arousal.

Beyond mapping existing evidence, this review also synthesizes how machine learning approaches have been used in cognitive state classification across multimodal studies. The resulting comparative framework can support researchers and practitioners in selecting appropriate EEG and ET metrics and understanding general modeling patterns suitable for developing cognitive-states monitoring systems. Furthermore, by highlighting underexplored modality combinations and inconsistent reporting practices, the review identifies key opportunities for advancing multimodal modeling and for standardizing data collection and analysis protocols in future research.

### 4.2 Limitations

Several limitations should be noted. First, the substantial variability across the included studies, such as differences in task paradigms, sensor configurations (e.g., EEG channel layouts or sampling rates), and extracted features, limited comparability and precluded a quantitative meta-analysis, even for conditions represented by multiple studies. In particular, as only a subset of EEG or ET metrics was reported in most studies, potentially informative features were not consistently analyzed across the literature. As a result, additional cross-study relationships may remain undetected, reinforcing the need for broader and more consistent extraction of EEG and ET metrics in future work.

Second, the underrepresentation of some conditions (such as stress or mind wandering) in the literature limited the strength of conclusions regarding these states and highlights the need for more research in these areas. Third, there was considerable variation in classification methodologies, including differences in labeling strategies or algorithm choice, which complicated direct comparison of model performance. Moreover, few studies assessed cross-subject validation, and those that did typically reported lower accuracy, suggesting limited generalization across individuals.

### 4.3 Implications

The findings support the integration of EEG and ET in applied settings where continuous vigilance is critical, such as aviation, defense, and nuclear power operations. Multimodal monitoring systems utilizing physiological signals may facilitate early detection of situational awareness and perception deterioration, thereby potentially preventing critical failures (Gumus and Saylam, 2023; Shaw and Harrell, 2023; Thomas and Russo, 2007). At the policy level, organizations dependent on sustained human attention should consider developing guidelines or allocating funding for research on real-time physiological monitoring technologies.

Future research should prioritize the development of coordinated, transparent, and multimodal standards. Establishing shared frameworks for signal synchronization, data preprocessing, and reporting, model after existing EEG and ET standards, would enhance reproducibility and comparability across studies. Moreover, protocols that assess multiple cognitive conditions within a single experimental context would clarify the overlaps and distinctions between perception-related states.

Although the studies reviewed rarely addressed real-time applicability directly, several principles can be outlined for the development of real-time EEG–ET monitoring systems. Preprocessing and artifact rejection must operate on short, sliding windows and be fully automated, and feature extraction pipelines should remain computationally lightweight to prevent delays and ensure timely classification outputs. Real-time systems also cannot rely on dataset-wide normalization. Instead, they might require adaptive calibration strategies such as subject-specific calibration phases or models trained on groups of participants with similar signal characteristics. Further investigation into real-time classification models, especially those employing machine learning or deep learning, may enable the creation of adaptive training environments and perception-aware interfaces. Follow-up studies and real-world validations are also essential for translating laboratory findings into operational applications.

### 4.4 Future directions: toward an integrative experimental protocol

A key challenge identified in the literature is the tendency to study perception-related conditions in isolation, even though they are conceptually and operationally interlinked. While many studies use similar EEG and ET metrics to investigate mental workload, stress, fatigue, or drowsiness, few evaluate these conditions concurrently or within the same subjects. This fragmentation hinders the development of reliable multimodal models for operational contexts, where such conditions frequently co-occur or evolve sequentially.

To address this issue, an integrative experimental framework is recommended. This framework should include: (1) concurrent induction of multiple cognitive states within the same participants using similar task designs, enabling the modeling of transitions and interactions between conditions; (2) harmonized multimodal pipelines, where EEG and ET are set up and processed following standardized procedures; and (3) adoption of benchmark labeling schemes and cross-condition validation protocols to systematically evaluate model generalizability across individuals, contexts, and hardware configurations. This framework would enable direct within-subject comparisons, assisting researchers in distinguishing between shared and condition-specific features in EEG and ET data. Over time, such an approach could support the creation of generalizable classification systems for perception deterioration markers.

### 4.5 Conclusions

This systematic review demonstrates that combining EEG and ET improves the detection and differentiation of closely related perception-related conditions. Future research should explore integrative experimental frameworks that evaluate multiple conditions within the same participants, capturing transitions between states as they co-occur or evolve. Finally, integrating machine learning with multimodal data for real-time classification offers significant potential for developing perception-aware monitoring systems to enhance safety and performance in operational settings.

## Data Availability

The original contributions presented in the study are included in the article/Supplementary material, further inquiries can be directed to the corresponding author.

## References

[B1] Acerra E. Pazzini M. Ghasemi N. Vignali V. Lantieri C. Simone A. . (2019). “EEG-based mental workload and perception-reaction time of the drivers while using adaptive cruise control,” in Human Mental Workload: Models and Applications, eds. L. Longo and M. C. Leva (Cham: Springer International Publishing), 226–239.

[B2] Aksu S. H. Çakit E. Dagdeviren M. (2024). Mental workload assessment using machine learning techniques based on EEG and eye tracking data. Appl. Sci. 14:6. doi: 10.3390/app14062282

[B3] Angkan P. Behinaein B. Mahmud Z. Bhatti A. Rodenburg D. Hungler P. . (2024). Multimodal brain–computer interface for in-vehicle driver cognitive load measurement: dataset and baselines. IEEE Trans. Intell. Transp. Syst. 25, 5949–5964. doi: 10.1109/TITS.2023.3345846

[B4] Arsen'ev G. N. Tkachenko O. N. Ukraintseva Y. V. Dorokhov V. B. (2015). Prediction of the moments at which critical decreases in levels of arousal occur using visuomotor coordination parameters. Neurosci. Behav. Physiol. 45, 715–723. doi: 10.1007/s11055-015-0134-4

[B5] Asish S. M. Kulshreshth A. K. Borst C. W. Sutradhar S. (2024). “Classification of internal and external distractions in an educational VR environment using multimodal features,” in IEEE Transactions on Visualization and Computer Graphics, Vol. 30 (Piscataway, NJ: IEEE), 7332–7342. 39255100 10.1109/TVCG.2024.3456207

[B6] Bafna T. Hansen J. P. (2021). Mental fatigue measurement using eye metrics: a systematic literature review. Psychophysiology 58:e13828. doi: 10.1111/psyp.13828 33825234

[B7] Barragan J. A. Yang J. Yu D. Wachs J. P. (2022). A neurotechnological aid for semi-autonomous suction in robotic-assisted surgery. Sci. Rep. 12:4504. doi: 10.1038/s41598-022-08063-w 35296714 PMC8927583

[B8] Bodala I. P. Li J. Thakor N. V. Al-Nashash H. (2016). EEG and eye tracking demonstrate vigilance enhancement with challenge integration. Front. Hum. Neurosci. 10:273. doi: 10.3389/fnhum.2016.00273 27375464 PMC4894919

[B9] Borys M. Plechawska-Wójcik M. Wawrzyk M. Wesołowska K. (2017a). “Classifying cognitive workload using eye activity and eeg features in arithmetic tasks,” in Information and Software Technologies, eds. R. Damaševičius and V. Mikašyte (Cham: Springer International Publishing), 90–105.

[B10] Borys M. Tokovarov M. Wawrzyk M. Wesołowska K. Plechawska-Wójcik M. Dmytruk R. . (2017b). “An analysis of eye-tracking and electroencephalography data for cognitive load measurement during arithmetic tasks,” in 2017 10th International Symposium on Advanced Topics in Electrical Engineering (ATEE) (Bucharest: IEEE), 287–292.

[B11] Chatterjee S. Scheck K. Küster D. Putze F. Moturu H. Schering J. . (2021). “SmartHelm: towards multimodal detection of attention in an outdoor augmented reality biking scenario,” in Companion Publication of the 2020 International Conference on Multimodal Interaction (New York, NY: Association for Computing Machinery) 426–432.

[B12] Chowdhury A. Shankaran R. Kavakli M. Md Haque M. (2018). Sensor applications and physiological features in drivers' drowsiness detection: a review. IEEE Sensors J. 18, 3055–3067. doi: 10.1109/JSEN.2018.2807245

[B13] Chua E. C.-P. Tan W.-Q. Yeo S.-C. Lau P. Lee I. Mien I. H. . (2012). Heart rate variability can be used to estimate sleepiness-related decrements in psychomotor vigilance during total sleep deprivation. Sleep 35:325. doi: 10.5665/sleep.1688 22379238 PMC3274333

[B14] Cohen M. X. (2017). Where does EEG come from and what does it mean? Trends Neurosci. 40, 208–218. doi: 10.1016/j.tins.2017.02.004 28314445

[B15] Córdoba A. C. Vidal M. R. Castellano A. M. S. Siale B. O. S. (2024). “Comprehensive study on fighter pilot attention and vigilance monitoring,” in Proceedings of the 2nd International Conference on Cognitive Aircraft Systems – ICCAS (Setúbal: SciTePress), 118–125.

[B16] Di Flumeri G. Borghini G. Aricò P. Sciaraffa N. Lanzi P. Pozzi S. . (2018). EEG-based mental workload neurometric to evaluate the impact of different traffic and road conditions in real driving settings. Front. Hum. Neurosci. 12:509. doi: 10.3389/fnhum.2018.00509 30618686 PMC6305466

[B17] Di Flumeri G. Borghini G. Aricò P. Sciaraffa N. Lanzi P. Pozzi S. . (2019a). “EEG-based mental workload assessment during real driving: a taxonomic tool for neuroergonomics in highly automated environments,” in The Brain at Work and in Everyday Life, eds. H. Ayaz and F. Dehai (New York: Academic Press), 121–126.

[B18] Di Flumeri G. De Crescenzio F. Berberian B. Ohneiser O. Kramer J. Aricò P. . (2019b). Brain–computer interface-based adaptive automation to prevent out-of-the-loop phenomenon in air traffic controllers dealing with highly automated systems. Front. Hum. Neurosci. 13:296. doi: 10.3389/fnhum.2019.00296 31555113 PMC6743225

[B19] Diaz-Piedra C. Rieiro H. Cherino A. Fuentes L. J. Catena A. Di Stasi L. L. (2019). The effects of flight complexity on gaze entropy: An experimental study with fighter pilots. Appl. Ergon. 77, 92–99. doi: 10.1016/j.apergo.2019.01.012 30832783

[B20] Doudou M. Bouabdallah A. Berge-Cherfaoui V. (2020). Driver drowsiness measurement technologies: current research, market solutions, and challenges. Int. J. Intell. Transp. Syst. Res. 18, 297–319. doi: 10.1007/s13177-019-00199-w

[B21] Elshafei A. Romano D. (2023). “A panoramic review of situational awareness monitoring systems,” in Proceedings of the 2023 6th International Conference on Robot Systems and Applications (New York, NY: Association for Computing Machinery), 56–61.

[B22] Endsley M. (2001). “Designing for situation awareness in complex system,” in Proceedings of the Second International Workshop on Symbiosis of Humans, Artifacts and Environment (Kyoto) 1–14.

[B23] Endsley M. R. (1995). Toward a theory of situation awareness in dynamic systems. Hum. Fact. 37, 32–64. doi: 10.1518/001872095779049543

[B24] Endsley M. R. Robertson M. M. (2000). “Training for situation awareness,” in in Situation Awareness Analysis and Measurement, eds., M. R. Endsley, and D. J. Garland (Boca Raton, CA: CRC Press), 349–365. Availble online at: https://www.pacdeff.com/pdfs/Training%20for%20SA%20Endsley%202000.pdf

[B25] Eniyandunmo D. Shin M. Lee C. Anwar A. Kim E. Kim K. . (2024). Utilising raw psycho-physiological data and functional data analysis for estimating mental workload in human drivers. Ergonomics 68, 602–618. doi: 10.1080/00140139.2024.2379949 39037945

[B26] Esposito A. Braccili E. Sgrò F. Chiarantano E. D'Ippolito M. Pisotta I. . (2022). “Attention, boredom and mind wandering during a vigilance task: EEG and ocular markers,” in 2022 IEEE International Conference on Metrology for Extended Reality, Artificial Intelligence and Neural Engineering (MetroXRAINE) (Rome: IEEE), 477–482.

[B27] Farha N. A. Al-Shargie F. Tariq U. Al-Nashash H. (2022). Brain region-based vigilance assessment using electroencephalography and eye tracking data fusion. IEEE Access 10, 112199–112210. doi: 10.1109/ACCESS.2022.3216407

[B28] Forbes C. Greenwood H. Carter M. Clark J. (2024). Automation of duplicate record detection for systematic reviews: deduplicator. Syst. Rev. 13:206. doi: 10.1186/s13643-024-02619-9 39095913 PMC11295717

[B29] Gaillard A. W. K. (1993). Comparing the concepts of mental load and stress. Ergonomics 36, 991–1005. doi: 10.1080/00140139308967972 8404841

[B30] Graafland M. Schraagen J. M. C. Boermeester M. A. Bemelman W. A. Schijven M. P. (2015). Training situational awareness to reduce surgical errors in the operating room. Br. J. Surg. 102, 16–23. doi: 10.1002/bjs.9643 25298183

[B31] Gradwell D. P. Wilkinson E. S. (2025). Ernsting's Aviation and Space Medicine. **Boca Raton, FL:** CRC Press.

[B32] Guede-Fernandez F. Fernandez-Chimeno M. Ramos-Castro J. Garcia-Gonzalez M. A. (2019). Driver drowsiness detection based on respiratory signal analysis. IEEE Access 7, 81826–81838. doi: 10.1109/ACCESS.2019.2924481

[B33] Gumus F. Saylam R. (2023). “Prevention of aviation accidents with prediction of cognitive states,” in 2023 10th International Conference on Recent Advances in Air and Space Technologies (RAST) (IEEE: Istanbul), 1–4.

[B34] Gündoǧdu S. Çolak Ö. H. Dogan E. A. Gülbetekin E. Polat Ö. (2021). Assessment of mental fatigue and stress on electronic sport players with data fusion. Med. Biol. Eng. Comput. 59, 1691–1707. doi: 10.1007/s11517-021-02389-9 34216320

[B35] Gündoǧdu S. Dogan E. A. Gülbetekin E. Halil Ç. Ö. Polat Ö. (2019). Evaluation of the EEG signals and eye tracker data for working different N-back modes. Trait. Sig. 36, 493–500. doi: 10.18280/ts.360603

[B36] Guo M. Duan P. Jin X. Huang Q. Wei Y. (2024). A performance-based mental workload identification method for special vehicle crews. Physiol. Behav. 288:114706. doi: 10.2139/ssrn.4857522 39349090

[B37] He Q. Li W. Fan X. Fei Z. (2016). Evaluation of driver fatigue with multi-indicators based on artificial neural network. IET Intell. Transp. Syst. 10, 555–561. doi: 10.1049/iet-its.2015.0021

[B38] Herrmann C. S. Strüber D. Helfrich R. F. Engel A. K. (2016). EEG oscillations: from correlation to causality. Int. J. Psychophysiol. 103, 12–21. doi: 10.1016/j.ijpsycho.2015.02.003 25659527

[B39] Holm A. Lukander K. Korpela J. Sallinen M. Müller K. M. I. (2009). Estimating brain load from the EEG. ScientificWorldJ. 9:973791. doi: 10.1100/tsw.2009.83 19618092 PMC5823228

[B40] Hong K.-S. Khan M. J. (2017). Hybrid brain–computer interface techniques for improved classification accuracy and increased number of commands: a review. Front. Neurorobot. 11:35. doi: 10.3389/fnbot.2017.00035 28790910 PMC5522881

[B41] Hopstaken J. F. van der Linden D. Bakker A. B. Kompier M. A. J. Leung Y. K. (2016). Shifts in attention during mental fatigue: evidence from subjective, behavioral, physiological, and eye-tracking data. J. Exp. Psychol. Hum. Percept. Perform. 42, 878–889. doi: 10.1037/xhp0000189 26752733

[B42] Huo X.-Q. Zheng W.-L. Lu B.-L. (2016). “Driving fatigue detection with fusion of EEG and forehead EOG,” in 2016 International Joint Conference on Neural Networks (IJCNN) (Vancouver, BC: IEEE), 897–904.

[B43] ICAO (2013). Fatigue Management. Availble online at: https://www.icao.int/NACC/Documents/eDOCS/FS/FS-Flyer_US-Letter_ANB-Fatigue-Management_2013-08-23.pdf

[B44] Iqbal M. U. Srinivasan B. Srinivasan R. (2024). Multi-class classification of control room operators' cognitive workload using the fusion of eye-tracking and electroencephalography. Comput. Chem. Eng. 181:108526. doi: 10.1016/j.compchemeng.2023.108526

[B45] Ismail L. E. Karwowski W. (2020). Applications of EEG indices for the quantification of human cognitive performance: a systematic review and bibliometric analysis. PLoS ONE 15:e0242857. doi: 10.1371/journal.pone.0242857 33275632 PMC7717519

[B46] Jackson A. F. Bolger D. J. (2014). The neurophysiological bases of EEG and EEG measurement: a review for the rest of us. Psychophysiology 51, 1061–1071. doi: 10.1111/psyp.12283 25039563

[B47] Jimenez-Molina A. Retamal C. Lira H. (2018). Using psychophysiological sensors to assess mental workload during web browsing. Sensors 18:2. doi: 10.3390/s18020458 29401688 PMC5855035

[B48] John A. R. Singh A. K. Do T.-T. N. Eidels A. Nalivaiko E. Gavgani A. M. . (2022). Unraveling the physiological correlates of mental workload variations in tracking and collision prediction tasks. IEEE Trans. Neural Syst. Rehab. Eng. 30, 770–781. doi: 10.1109/TNSRE.2022.3157446 35259108

[B49] Jüni P. Loke Y. Pigott T. Ramsay C. Regidor D. Rothstein H. . (2016). Risk of bias in non-randomized studies of interventions (ROBINS-I): detailed guidance. Br. Med. J. 355:i4919.27733354

[B50] Kalaganis F. P. Georgiadis K. Oikonomou V. P. Laskaris N. A. Nikolopoulos S. Kompatsiaris I. (2021). Unlocking the subconscious consumer bias: a survey on the past, present, and future of hybrid EEG schemes in neuromarketing. Front. Neuroergon. 2:672982. doi: 10.3389/fnrgo.2021.672982 38235255 PMC10790945

[B51] Kam J. W. Y. Rahnuma T. Park Y. E. Hart C. M. (2022). Electrophysiological markers of mind wandering: a systematic review. NeuroImage 258:119372. doi: 10.1016/j.neuroimage.2022.119372 35700946

[B52] King A. J. Bol,N Cummins R. G. John K. K. (2019). Improving visual behavior research in communication science: an overview, review, and reporting recommendations for using eye-tracking methods. Commun. Methods Meas. 13, 149–177. doi: 10.1080/19312458.2018.1558194

[B53] Kirschstein T. Köhling R. (2009). What is the source of the EEG? Clin. EEG Neurosci. 40, 146–149. doi: 10.1177/155005940904000305 19715175

[B54] Klein C. Ettinger U. (Eds.). (2019). Eye Movement Research: An Introduction to its Scientific Foundations and Applications. Cham: Springer International Publishing.

[B55] Kujur A. Bhattacharya A. Sharma G. Kumar J. (2022). “Prediction of workload under distraction using supervised learning algorithms,” in 2022 3rd International Conference on Issues and Challenges in Intelligent Computing Techniques (ICICT) (Ghaziabad: IEEE), 1–5.

[B56] Kula I. Atkinson R. K. Roscoe R. D. Branaghan R. J. (2017). “A biometric usability evaluation of instrument cluster and infotainment systems in two hybrid cars,” in 2017 IEEE SmartWorld, Ubiquitous Intelligence and Computing, Advanced and Trusted Computed, Scalable Computing and Communications, Cloud and Big Data Computing, Internet of People and Smart City Innovation (SmartWorld/SCALCOM/UIC/ATC/CBDCom/IOP/SCI) (San Francisco, CA: IEEE), 1–6.

[B57] Kunasegaran K. Ismail A. M. H. Ramasamy S. Gnanou J. V. Caszo B. A. Chen P. L. (2023). Understanding mental fatigue and its detection: a comparative analysis of assessments and tools. PeerJ, 11:e15744. doi: 10.7717/peerj.15744 37637168 PMC10460155

[B58] Kuvar V. Kam J. W. Y. Hutt S. Mills C. (2023). “Detecting when the mind wanders off task in real-time: an overview and systematic review,” in Proceedings of the 25th International Conference on Multimodal Interaction (New York, NY: Association for Computing Machinery), 163–173.

[B59] Larue G. S. Rakotonirainy A. Pettitt A. N. (2015). Predicting reduced driver alertness on monotonous highways. IEEE Pervasive Comput. 14, 78–85. doi: 10.1109/MPRV.2015.38

[B60] Lean Y. Shan F. (2012). Brief review on physiological and biochemical evaluations of human mental workload. Hum. Fact. Ergon. Manuf. Serv. Indus. 22, 177–187. doi: 10.1002/hfm.20269

[B61] Li B. Wang X. Wu Y. Zhu X. (2023). “Research on driver KSS rating prediction model based on EU 2021/1341 DDAW,” in 2023 International Conference on Artificial Intelligence and Automation Control (AIAC) (Xiamen: IEEE), 195–201.

[B62] Lobo J. L. Ser J. D. De Simone F. Presta R. Collina S. Moravek Z. (2016). “Cognitive workload classification using eye-tracking and EEG data,” in Proceedings of the International Conference on Human-Computer Interaction in Aerospace (Association for Computing Machinery: Paris), 1–8.

[B63] Mark J. A. Curtin A. Kraft A. E. Ziegler M. D. Ayaz H. (2024). Mental workload assessment by monitoring brain, heart, and eye with six biomedical modalities during six cognitive tasks. Front. Neuroergon. 5:1345507. doi: 10.3389/fnrgo.2024.1345507 38533517 PMC10963413

[B64] Martinez-Marquez D. Pingali S. Panuwatwanich K. Stewart R. A. Mohamed S. (2021). Application of eye tracking technology in aviation, maritime, and construction industries: a systematic review. Sensors 21:13. doi: 10.3390/s21134289 34201734 PMC8271947

[B65] Matthews G. Reinerman-Jones L. E. Barber D. J. Abich J. (2015). The psychometrics of mental workload: multiple measures are sensitive but divergent. Hum. Fact. 57, 125–143. doi: 10.1177/0018720814539505 25790574

[B66] McGuinness L. A. Higgins J. P. T. (2020). Risk-of-bias VISualization (robvis): an R package and Shiny web app for visualizing risk-of-bias assessments. Res. Synth. Methods 12, 55–61. doi: 10.1002/jrsm.1411 32336025

[B67] Mehrabi E. Kim J.-E. (2022). “Physiological measurements of vigilance: a systematic review,” in Proceedings of the Human Factors and Ergonomics Society Annual Meeting, Vol. 66 (Thousand Oaks, CA: Sage Publishing), 823–827.

[B68] Mohammadfam I. Mahdinia M. Soltanzadeh A. Mirzaei Aliabadi M. Soltanian A. R. (2021). A path analysis model of individual variables predicting safety behavior and human error: the mediating effect of situation awareness. Int. J. Indus. Ergon. 84:103144. doi: 10.1016/j.ergon.2021.103144

[B69] Naderpour M. Nazir S. Lu J. (2015). The role of situation awareness in accidents of large-scale technological systems. Process Saf. Environ. Prot. 97, 13–24. doi: 10.1016/j.psep.2015.06.002

[B70] Nirabi A. Rahman F. A. Habaebi M. H. Azami K. Yusoff S. H. (2022). EEG signal analysis for mental stress classification: a review. J. Theor. Appl. Inf. Technol. 100, 6199–6214.

[B71] Oken B. S. Salinsky M. C. Elsas S. M. (2006). Vigilance, alertness, or sustained attention: physiological basis and measurement. Clin. Neurophysiol. 117, 1885–1901. doi: 10.1016/j.clinph.2006.01.017 16581292 PMC2865224

[B72] Orlandi L. Brooks B. (2018). Measuring mental workload and physiological reactions in marine pilots: building bridges towards redlines of performance. Appl. Ergon. 69, 74–92. doi: 10.1016/j.apergo.2018.01.005 29477333

[B73] Page M. J. McKenzie J. E. Bossuyt P. M. Boutron I. Hoffmann T. C. Mulrow C. D. . (2021). The PRISMA 2020 statement: an updated guideline for reporting systematic reviews. BMJ 372:n71. doi: 10.1136/bmj.n71 33782057 PMC8005924

[B74] Pan Y. Guo Z. Zhao Y. Zhou M. Yang L. Zhang J. . (2024). A 2-D vigilance estimation method for high-speed rail drivers with multimodal sensors. IEEE Sens. J. 24, 28982–28994. doi: 10.1109/JSEN.2024.3433566

[B75] Pei X. Xu G. Zhou Y. Tao L. Cui X. Wang Z. . (2022). A simultaneous electroencephalography and eye-tracking dataset in elite athletes during alertness and concentration tasks. Sci. Data 9:465. doi: 10.1038/s41597-022-01575-0 35918334 PMC9345900

[B76] Planke L. J. Gardi A. Sabatini R. Kistan T. Ezer N. (2021). Online multimodal inference of mental workload for cognitive human machine systems. Computers 10:6. doi: 10.3390/computers10060081

[B77] Popa L. Selejan O. Scott A. Mureşanu D. F. Balea M. Rafila A. (2015). Reading beyond the glance: eye tracking in neurosciences. Neurol. Sci. 36, 683–688. doi: 10.1007/s10072-015-2076-6 25604577

[B78] Poudel G. R. Innes C. R. Bones P. J. Watts R. Jones R. D. (2012). Losing the struggle to stay awake: divergent thalamic and cortical activity during microsleeps. Hum. Brain Mapp. 35:257. doi: 10.1002/hbm.22178 23008180 PMC6869765

[B79] Poudel G. R. Innes C. R. H. Bones P. J. Jones R. D. (2010). The relationship between behavioural microsleeps, visuomotor performance and EEG theta. Annu. Int. Conf. IEEE Eng. Med. Biol. 2010, 4452–4455. doi: 10.1109/IEMBS.2010.5625956 21095769

[B80] Previc F. H. Lopez N. Ercoline W. R. Daluz C. M. Workman A. J. Evans R. H. . (2009). The effects of sleep deprivation on flight performance, instrument scanning, and physiological arousal in pilots. Int. J. Aviat. Psychol. 19, 326–346. doi: 10.1080/10508410903187562

[B81] Rafid A.-U.-I. Raha Niloy A. Chowdhury A. I. Sharmin N. (2020). A brief review on different driver's drowsiness detection techniques. Int. J. Image Graph. Sig. Process. 12, 41–50. doi: 10.5815/ijigsp.2020.03.05

[B82] Reichle E. D. Reineberg A. E. Schooler J. W. (2010). Eye movements during mindless reading. Psychol. Sci. 21, 1300–1310. doi: 10.1177/0956797610378686 20679524

[B83] Reßing C. Oschinsky F. M. Klesel M. Niehaves B. Riedl R. Suwandjieff P. . (2022). “Investigating mind-wandering episodes while using digital technologies: an experimental approach based on mixed-methods,” in Information Systems and Neuroscience, eds. F. D. Davis, R. Riedl, J. vom Brocke, P.-M. Léger, A. B. Randolph, and G. R. Müller-Putz (Cham: Springer International Publishing), 301–309.

[B84] Rodriguez A. C. Lee D. A. Makic M. B. F. (2017). Situational awareness in critical care: an aviation approach to reduce error. J. PeriAnesth. Nurs. 32, 650–652. doi: 10.1016/j.jopan.2017.08.001 29157771

[B85] Schad D. J. Nuthmann A. Engbert R. (2012). Your mind wanders weakly, your mind wanders deeply: objective measures reveal mindless reading at different levels. Cognition 125, 179–194. doi: 10.1016/j.cognition.2012.07.004 22857818

[B86] Sengupta A. Dasgupta A. Chaudhuri A. George A. Routray A. Guha R. (2017). A multimodal system for assessing alertness levels due to cognitive loading. IEEE Trans. Neural Syst. Rehab. Eng. 25, 1037–1046. doi: 10.1109/TNSRE.2017.2672080 28237931

[B87] Sengupta K. Sun J. Menges R. Kumar C. Staab S. (2017). “Analyzing the impact of cognitive load in evaluating gaze-based typing,” in 2017 IEEE 30th International Symposium on Computer-Based Medical Systems (CBMS) (Piscataway, NJ: IEEE), 787–792.

[B88] Shafiei S. B. Shadpour S. Mohler J. L. (2024). An integrated electroencephalography and eye-tracking analysis using eXtreme gradient boosting for mental workload evaluation in surgery. Hum. Fact. 67, 464–484. doi: 10.1177/00187208241285513 39325959 PMC11936844

[B89] Shaw D. M. Harrell J. W. (2023). Integrating physiological monitoring systems in military aviation: a brief narrative review of its importance, opportunities, and risks. Ergonomics 66, 2242–2254. doi: 10.1080/00140139.2023.2194592 36946542

[B90] Singh G. Chanel C. P. C. Roy R. N. (2021). Mental workload estimation based on physiological features for Pilot-UAV teaming applications. Front. Hum. Neurosci. 15:692878. doi: 10.3389/fnhum.2021.692878 34489660 PMC8417701

[B91] Skaramagkas V. Giannakakis G. Ktistakis E. Manousos D. Karatzanis I. Tachos N. S. . (2023). Review of eye tracking metrics involved in emotional and cognitive processes. IEEE Rev. Biomed. Eng. 16, 260–277. doi: 10.1109/RBME.2021.3066072 33729950

[B92] Smallwood J. Schooler J. W. (2015). The science of mind wandering: empirically navigating the stream of consciousness. Annu. Rev. Psychol. 66, 487–518. doi: 10.1146/annurev-psych-010814-015331 25293689

[B93] Soufineyestani M. Dowling D. Khan A. (2020). Electroencephalography (EEG) technology applications and available devices. Appl. Sci. 10:21. doi: 10.3390/app10217453

[B94] Sterne J. A. Hernán M. A. Reeves B. C. Savović J. Berkman N. D. Viswanathan M. . (2016). ROBINS-I: a tool for assessing risk of bias in non-randomised studies of interventions. BMJ 355:i4919. doi: 10.1136/bmj.i4919 27733354 PMC5062054

[B95] Tao D. Tan H. Wang H. Zhang X. Qu X. Zhang T. (2019). A systematic review of physiological measures of mental workload. Int. J. Environ. Res. Public Health 16:15. doi: 10.3390/ijerph16152716 PMC6696017 31366058

[B96] Thomas M. L. Russo M. B. (2007). Neurocognitive monitors: toward the prevention of cognitive performance decrements and catastrophic failures in the operational environment. Aviat. Space Environ. Med. 78, B144–152. 17547315

[B97] Tolvanen O. Elomaa A.-P. Itkonen M. Vrzakova H. Bednarik R. Huotarinen A. (2022). Eye-tracking indicators of workload in surgery: a systematic review. J. Investig. Surg. 35, 1340–1349. doi: 10.1080/08941939.2021.2025282 35038963

[B98] Torkamani-Azar M. Lee A. Bednarik R. (2022). Methods and measures for mental stress assessment in surgery: a systematic review of 20 years of literature. IEEE J. Biomed. Health Inform. 26, 4436–4449. doi: 10.1109/JBHI.2022.3182869 35696473

[B99] Tran Y. Craig A. Craig R. Chai R. Nguyen H. (2020). The influence of mental fatigue on brain activity: evidence from a systematic review with meta-analyses. Psychophysiology 57:e13554. doi: 10.1111/psyp.13554 32108954

[B100] Yang Y. Chen Y. Wu C. Easa S. M. Lin W. Zheng X. (2020). Effect of highway directional signs on driver mental workload and behavior using eye movement and brain wave. Accid. Anal. Prev. 146:105705. doi: 10.1016/j.aap.2020.105705 32818759

[B101] Zaky M. H. Shoorangiz R. Poudel G. R. Yang L. Innes C. R. H. Jones R. D. (2023). Increased cerebral activity during microsleeps reflects an unconscious drive to re-establish consciousness. Int. J. Psychophysiol. 189, 57–65. doi: 10.1016/j.ijpsycho.2023.05.349 37192708

[B102] Zaky M. H. Shoorangiz R. Poudel G. R. Yang L. Jones R. D. (2021). Investigating the neural signature of microsleeps using EEG. Annu. Int. Conf. IEEE Eng. Med. Biol. Soc. 2021, 6293–6296. doi: 10.1109/EMBC46164.2021.9630401 34892552

[B103] Zandi A. S. Quddus A. Prest L. Comeau F. J. E. (2019). Non-intrusive detection of drowsy driving based on eye tracking data. Transp. Res. Rec. 2673, 247–257. doi: 10.1177/0361198119847985

[B104] Zhang H. Dong E. Tong J. Yang S. Du S. (2023). “Fatigue driving detection of EEG signals by LSTM deep neural network with LPSD and DE,” in 2023 IEEE International Conference on Mechatronics and Automation (ICMA) (Harbin: IEEE), 1108–1112.

[B105] Zhang T. Yang J. Liang N. Pitts B. J. Prakah-Asante K. Curry R. . (2020). Physiological measurements of situation awareness: a systematic review. Hum. Fact. 65, 737–758. doi: 10.1177/0018720820969071 33241945

[B106] Zhu H.-X. (2021). “EEG functional connectivity predicts continuous fatigue levels during underload task,” in 2021 International Conference on Artificial Intelligence and Electromechanical Automation (AIEA) (Piscataway, NJ: IEEE), 322–327.

[B107] Ziv G. (2016). Gaze behavior and visual attention: a review of eye tracking studies in aviation. Int. J. Aviat. Psychol. 26, 75–104. doi: 10.1080/10508414.2017.1313096

