# Supplementary material for: Combining EEG and eye-tracking for cognitive and physiological states monitoring: a systematic review
Source: Front Neuroergon. 2026 Jan 29;6:1736672. doi: 10.3389/fnrgo.2025.1736672 (PMC12895110; doi:10.3389/fnrgo.2025.1736672)
Supplement: Supplementary file 1 [file Table_1.pdf]

DATA COLLECTION

1. STRESS AND FATIGUE

TABLE 1.1. EXPERIMENTATION DETAILS

Study	Participants	Condition(s)	Collected Data	EEG Channels	Type of ET signal
(Gündoğdu et al., 2021)	**N:** 10 (8 male, 2 female)<br>**Age:** 24-42, avg 32.3 ± 7.5<br>**Data problems:** EEG of 1 participant	Stress, mental fatigue, and attention measures during e-sport activity (test battery: VAS, d2 test, N-back test, electronic sport game task)	**Physiological measures:** EEG, ET, EDA, HRV<br>**Cognitive/performance-based measures:** D2 test, N-back test<br>**Subjective measures:** VAS	4-channel EEG headband (10-20 system): AF7 and AF8, used (**prefrontal region**); 1 reference point (FPz)<br>Sampling rate: 256 Hz	Remote ET<br>Sampling rate: 30 Hz<br>**Eye Gaze:** heatmaps
(Gündoğdu et al., 2019)	**N:** 8 (6 male, 2 female)<br>**Age:** 24-41, avg 31 ± 7.3<br>**Data problems:** EEG of 1 participant	Stress and mental fatigue during 3 N-back test tasks (Position single task, Position-Color 2-Back task and Position Image 2-Back task)	**Physiological measures:** EEG, ET<br>**Subjective measures:** VAS	4-channel EEG headband (10-20 system): AF7, AF8, TP9, TP10 (**prefrontal and temporal regions**); 1 reference point (FPz)<br>Sampling rate: 256 Hz	Head-mounted ET<br>Sampling rate: 30 Hz<br>**Eye Gaze:** heatmaps
(Hopstaken et al., 2016)	**N:** 47 (32 female, 15 male)<br>**Age:** 18-25, avg 20.5 ± 1.8<br>**Data problems:** EEG of 10 participants, ET of 12	Mental fatigue during a cognitive task: time-on-task paradigm (N-back task, visual letter 2-back task) with alternative task-unrelated stimuli (reward stimuli) to examine attentional disengagement	**Physiological measures:** EEG, ET, hEOG, vEOG<br>**Cognitive/performance-based measures:** N-back test<br>**Subjective measures:** RSME (subjective fatigue and engagement)	32-channel EEG (10-20 system): 2 reference points (mastoids); 2 hOEG electrodes, 2 vEOG electrodes (ocular movement artifact correction)<br>Sampling rate: 512 Hz	Remote ET<br>Sampling rate: 60 Hz<br>**Eye Gaze:** gaze position (AOIs)<br>**Pupil Dilation:** pupil diameter
(Previc et al., 2009)	**N:** 10 (all male)<br>**Age:** 23-46, avg 34.2<br>**Other:** All USAF pilots	Fatigue during sleep deprivation	**Physiological measures:** EEG, ET<br>**Cognitive/performance-based measures:** RMSE for each instrument<br>**Subjective measures:** POMS, VAS	2-channels: Cz, Pz (**central and parietal regions**); 2 reference points (mastoids), ground lead (scalp)<br>Sampling rate: 200 Hz	Head-mounted ET<br>Sampling rate: 60 Hz<br>**Eye Gaze:** fixations, saccades, scanning pattern, transitioning measures<br>**Eyelid Opening:**

Study	Participants	Condition(s)	Collected Data	EEG Channels	Type of ET signal
					blinks<br>**Pupil Dilation:** pupil diameter
(Li et al., 2023)	**N:** 35 (24 male, 11 female)<br>**Age:** 20-50+	Fatigue based on KSS drowsiness level	**Physiological measures:** EEG, ET<br>**Cognitive/performance-based measures:** PVT, AAT, V-P300, A-P300<br>**Subjective measures:** VAS, KDT, KSS-CN	64-channel EEG	Remote ET<br>**Eyelid Opening:** PERCLOS
(Huo et al., 2016)	**N:** 21 (12 male, 9 female)<br>**Age:** 20-25	Fatigue during driving	**Physiological measures:** EEG, ET, EOG	12-channel EEG (10-20 system): CP1, CPz, CP2, P1, Pz, P2, PO3, POz, PO4, O1, Oz, O2 (**central parietal, parietal, occipital and parieto-occipital regions**); 1 reference point (top of scalp)<br>Sampling rate: 1000 Hz	Head-mounted ET<br>**Eyelid Opening:** PERCLOS
(Zhu, 2021)	**N:** 15 (7 male, 8 female)<br>**Age:** avg 23.27 ± 2.37<br>**Data problems**: ET of 3 participants	Fatigue during driving	**Physiological measures:** EEG, ET	64-channel EEG (10-20 system): 17 selected, 11 from **posterior site** (C31, Cz, C4, P3, Pz, P4, PO3, POz, PO4, O1, O2) and 6 from **temporal** site (F3, F4, FC5, FC6, CP5, CP6)<br>Sampling rate: 200 Hz	Head-mounted ET<br>**Eyelid Opening:** PERCLOS
(He et al., 2016)	**N:** 50<br>**Age:** 25-30, avg 28.48 ± 2.63	Fatigue during driving	**Physiological measures:** EEG, ET, head nodding angle<br>**Cognitive/performance-based measures:** driving time	16-channel EEG<br>Sampling rate: 1000 Hz	Remote ET<br>**Eyelid Opening:** PERCLOS
(Zhang et al., 2023)	**N:** 8 (6 male, 2 female)<br>**Age:** 22-25	Fatigue during driving	**Physiological measures:** EEG, ET	64-channel EEG (10-20 system)<br>Sampling rate: 1000 Hz	Remote ET<br>Sampling rate: 250 Hz<br>**Eyelid Opening:** PERCLOS

TABLE 1.2. IMPACT OF CONDITION ON FEATURES

Study	Condition(s)	EEG Features	ET Features	Other Features
(Gündoğdu et al., 2021)	Stress and Mental fatigue	**Power values of sub-bands (AF7 and AF8 channels), power ratios:**<br>- Alpha, beta, theta, alpha + theta, and alpha + beta + theta powers increased during gaming (stress and mental fatigue status) compared to resting<br>- Decreased Alpha/Beta Ratio during gaming (greater increase in beta activity relative to alpha activity)<br>- EEG from the frontal region indicates that the game is partly cause by stress and mental fatigue	**Gaze Heatmaps:** Focused Gaze Heatmap with High Performance; Spread with Low Performance	**ECG features:**<br>- Mean RR (IBI), SDNN, RMSSD, LF, and HF decreased during rest (decrease of RR intervals during game indicative of mental fatigue)<br>- Average HR and LF/HF values increased during rest<br>- HRV analysis: different sympathetic and vagal activities created by the game<br>**EDA features:** Skin conductivity statistical values (except for skewness) increased during game<br>**EDA + ECG (HRV):** Emotional processes of participants: some stress and some excited<br>**VAS:** scores for stress and mental fatigue increased after the game<br>**D2 test:** Concentration performance increased after the game (the game has positive effect on attention and concentration)<br>**N-back task:** ‘Position color 2-back’ score decreased after the game (indicative of mental fatigue)
(Gündoğdu et al., 2019)	Stress and Mental fatigue	**Power values of sub-bands (AF7 and AF8 channels):**<br>- Beta, alpha, and theta powers increased with test score and difficulty level<br>- AF7 region more dominant than AF8<br>- AF8 power values higher for participants with high test scores	**Gaze Heatmaps:** Gaze Heatmap spreads as test difficulty increases (focus differences with changes in difficulty levels and test modes)	**VAS:** VAS score for mental fatigue and stress increased after N-back test with low and high test scores (N-back tests increased stress and mental fatigue)
(Hopstaken et al., 2016)	Mental fatigue	**P3 peak activity (Pz electrode):**<br>- P3b amplitude: decline with increasing time-on-task for blocks (decreased task engagement)	**Gaze position:**<br>**-** No significant decrease in gaze toward task-related stimuli with increasing time-on-task:	**RSME (Subjective):**<br>- Higher levels of fatigue after the experiment<br>- Increased fatigue with increasing time-on-task for blocks (before reward manipulation)

Study	Condition(s)	EEG Features	ET Features	Other Features
			increased percentage of off-screen and missing gaze position on task-related areas, but not in the others (increased fatigue not associated with decreased time focused on task-related areas)<br>**Pupil data:**<br>- Baseline pupil diameter significantly decreased with time-on-task	- Decreased task engagement with increasing time-on-task (before reward manipulation)<br>**N-back task: accuracy, RT**<br>**-** Decreased performance with increasing time-on-task: d-prime (fatigue compromised cognitive performance) and RT decrease (partial speed/accuracy trade-off)
(Previc et al., 2009)	Fatigue	**Power values of sub-bands delta, theta and alpha (Cz and Pz channels):**<br>- Alpha power decreased as wakefulness increased<br>- Delta and theta power increased as wakefulness increased<br>- Alpha power decline in early-morning<br>- Significant effect of flight session for theta and alpha at Pz<br>**-** Correlation between Pz and theta	**Ocular scanning patterns (instrument scanning and transitioning measures):**<br>**-** Dwell-time percentage consistent with maneuver (sensitive to expected demands).<br>**-** Consistent scanning behavior across pilots.<br>**-** Instrument scanning patterns similar for early and late flights (mostly unaffected by sleep deprivation: instrument scanning resistant to fatigue, maybe due to practiced behavior)<br>**Basic eye movement parameters (blink rate, pupil diameter, avg saccade length, avg fixation time, and percentage of long dwells):** No significant changes (maybe due to large variability across pilots in early versus later oculomotor behavior)	**Subjective Fatigue Ratings (POMS fatigue dimension and VAS sleepiness scales):**<br>- Increased fatigue/sleepiness over time<br>- Subjective fatigue peak in early-morning<br><br>**Flight Performance (composite RMSE):**<br>- Decrease over time: flight performance showed initial improvement early on, followed by a steady. decline across flights (deterioration in flight performance during fatigue)<br>- Flying errors' peak in early-morning: temporary flight performance decrease<br>- Subsequent rebound in flight performance (possible effect of circadian cycle, not only sleep deprivation)

Study	Condition(s)	EEG Features	ET Features	Other Features
(Li et al., 2023)	Fatigue	**Differential entropy (DE):** Energy of alpha band with the increase of drowsiness: increases (open eyes state); decreases (closed eyes state)	**PERCLOS:** Increases with the increase of drowsiness	**KSS:**<br>**-** Drowsiness increases with the improvement of KSS rating<br>**-** No significant difference between KSS 1-3 level and KSS 4-5 level; significant differences between KSS 6 level and above<br>**VAS:** score increases with the increase of drowsiness (KSS rating)<br>**Neural RT:** RT slows down and number of mistakes increases with the increase of drowsiness
(Huo et al., 2016)	Fatigue	**PSD and DE:** DE feature more accurate and stable than PSD feature for EEG-based driving fatigue detection	**PERCLOS:** not reported effect	**EOG:** features about blink, fixation, and saccade (mean, maximum, variance, and derivative of blink, fixation, and saccade)
(Zhu, 2021)	Fatigue	**Power values of sub-bands delta, theta, alpha, and beta; connectivity (based on PLI)**<br>Significant change in alpha band	**PERCLOS**	NA
(He et al., 2016)	Fatigue	**Power values of sub-bands; power ratio (theta + alpha)/beta**	**PERCLOS**	**Head nodding angle: nodding angle, nodding frequency**<br><br>**Driving time-related indicators: time-of-day, time-on-task**
(Zhang et al., 2023)	Fatigue	**LPSD, DE (of sub-bands)**	**PERCLOS**<br>PERCLOS index (0.1 s interval): >0.7 (fatigue), <0.7 (normal)	NA

TABLE 1.3. CLASSIFIERS AND PERFORMANCE

Study	Labels	Classifier(s)	Performance metrics	Cross-validation	Results	Modalities comparison
(Li et al., 2023)	4 fatigue levels according to	SVM, linear SVM, kNN, LR	Accuracy, precision, recall	50% (training set split)	- Linear SVM: accuracy 0.7273, precision 0.39, recall 0.557<br>- SVM: 0.6909, 0.1727, 0.25	NA

Study	Labels	Classifier(s)	Performance metrics	Cross-validation	Results	Modalities comparison
	KSS score: 1-5, 6, 7, 8-9				- kNN: 0.6909, 0.1727, 0.25<br>- LR: 0.768, 0.629, 0.754 (best)	
(Huo et al., 2016)	Fatigue level (based on PERCLOS)	GELM, SVM	Prediction CC, RMSE	5-k	GELM performance on each modality better than SVM	Modality fusion (EEG+EOG) achieve higher prediction CCs and lower RMSE values than that of the single modalities
(Zhu, 2021)	Alert vs Fatigue (based on connectivity EEG, PLI value)	NN	MAE	NA	EEG functional connection method had dominant performance over traditional one; Beta band best performance:<br>**All frequency bands:**<br>- Clustering coefficient: 3.525e-6 MAE<br>- Global integration, characteristic path length: 1.064e-5<br>- Global interaction, degree: 2.192e-4<br>**Alpha:**<br>- Clustering coefficient: 3.223e-6<br>- Global integration, characteristic path length: 3.619e-5<br>- Global interaction, degree: 1.361e-6<br>**Beta:**<br>- Clustering coefficient: 1.659e-5<br>- Global integration, characteristic path length: 3.787e-6<br>- Global interaction, degree: 3.897e-6	NA
(He et al., 2016)	Alert vs Drowsy (based on EEG)	ANN	Accuracy	NA	**head-based model:**<br>- 84.1% accuracy (alert state) and 83.5% accuracy (drowsy state)<br>**PERCLOS-based model:**<br>- 89.2% accuracy (alert state) and 88.9% accuracy (drowsy state)<br>**All factors model (including driving time indicators):**<br>- 93.4% accuracy (alert state) and 93.7% accuracy (drowsy state)	PERCLOS have significant higher accuracy than that with head-based indicator; all-factors significantly more efficient than single-modality

Study	Labels	Classifier(s)	Performance metrics	Cross-validation	Results	Modalities comparison
(Zhang et al., 2023)	Fatigue vs normal state (based on PERCLOS)	LSTM (3 layers), PSD-SVM	Accuracy	5-k (50-50 test training slip)	LSTM: 93.12%<br>PSD-SVM: 74.35%	NA

2. MIND WANDERING

TABLE 2.1. EXPERIMENTATION DETAILS

Study	Participants	Condition(s)	Collected Data	EEG Channels	Type of ET signal
(Esposito et al., 2022)	**N:** 15 (8 male, 7 female)<br>**Age:** avg 27 ± 0.9	Attention, boredom and mind wandering during vigilance and sustained attention test: MCT (adapted for boredom and mind wandering inclusion)	**Physiological measures:** EEG, ET<br>**Cognitive/performance-based measures:** RT and detection accuracy (hit rate)<br>**Subjective measures:** self-reports (attention, boredom, and mind wandering scores)	32-channel EEG (10-20 system); 1 reference point (right ear lobe)<br>Sampling rate: 500 Hz	Remote ET<br>Sampling rate: 30 Hz<br>**Eyelid Opening:** blinks<br>**Pupil Dilation:** pupil size
(Asish et al., 2024)	**N:** 27 (17 male, 10 female)<br>**Age:** 18-30, avg 21.4 ± 2.91<br>**Other:** 18 participants with prior VR experience	Internal and external distractions in educational VR environments	**Physiological measures:** EEG, ET<br>**Subjective measures:** questionnaires	16-channel EEG (10-20 system): used 7 channels in **occipital, parietal, and prefrontal regions** (FP1, FP2, P3, P4, Pz, O1 and O2)<br>Sampling rate: 125 Hz	Head-mounted ET<br>Sampling rate: 120 Hz<br>**Eye Gaze:** direction, position, origin, angles<br>**Eyelid Opening:** Eye Openness, Eye Wideness<br>**Pupil Dilation:** Pupil Diameter
(Reßing et al., 2022)	**N:** 45 (24 female, 21 male)<br>**Age:** 21-47, avg 29.2	Mind wandering while using digital technologies during divergent thinking tasks: UTT	**Physiological measures:** EEG, ET<br>**Cognitive/performance-based measures:** performance data<br>**Subjective measures:** questionnaires	NA	Head-mounted ET<br>**Eye Gaze:** Gaze fixations

TABLE 2.2. IMPACT OF CONDITION ON FEATURES

Study	Condition(s)	EEG Features	ET Features	Other Features
(Esposito et al., 2022)	Attention, Boredom, and Mind wandering	**Power values of sub-bands theta, alpha and beta (Fz, Cz, Pz, Oz channels – midline channels)**<br>**Theta power:**<br>- Negative correlation with hit rate (Fz, Cz, Pz)<br>- Positive correlation with RT (Fz, Pz)<br>- Positive correlation with boredom (all channels)	**Blinks:**<br>- Negative correlation between number and duration of blinks and hit rate score<br>- Negative correlation between blink count and attention score	**Self-reports:**<br>- Negative correlation between subjective boredom and attention (higher boredom alongside lower attention)<br>- Lower attention not related to mind wandering episodes

Study	Condition(s)	EEG Features	ET Features	Other Features
		- Theta power on Fz good predictor of boredom<br>- Correlation with attention (Oz)<br>- No significant correlation with mind wandering<br>- Theta power on midline channels good predictor of boredom and performance (Cz of RT; Pz of hit rate; Fz of boredom)<br>**Alpha power:**<br>- Negative correlation with hit rate (Fz, Cz, Pz)<br>- Positive correlation with mind-wandering (Fz, Pz)<br>- Positive correlation with boredom (Pz)<br>- Alpha power on midline channels good discriminant of low and high levels of boredom and mind wandering (increase in Pz and Oz during high boredom; increase in Fz and Pz in high mind wandering)<br>**Beta power:**<br>- Negative correlation with hit rate (Fz, Cz, Pz)	- Positive correlation between blink count and boredom score<br>- No significant correlation between ocular features and mind wandering<br>- Blink count good predictor of attention and boredom<br>- Blink duration good predictor of hit rate<br>**Pupil size:** no significant results (possibly affected by mean pupil diameter not considering temporal dynamics)	(probably proposed paradigm failed in detecting these episodes)<br>- Positive correlation between boredom and mind wandering (higher incidence of mind wandering episodes when boredom)<br>**Performance:**<br>- Negative correlation between RT and hit rate (higher test accuracy alongside faster response)<br>- Improved performance with (subjective) attention<br>- Decreased performance with (subjective) boredom
(Asish et al., 2024)	External and Internal distractions (Mind wandering)	Greater contributions from:<br>- Occipital and prefrontal regions of the brain (O2, FP1, Pz)	Greater contributions from:<br>- Gaze angle towards the avatar (visual attention: focus on specific objects, characters or events)<br>- Gaze origin and head rotation features (visual exploration, engagement, immersion)	
(Reßing et al., 2022)	Mind wandering	**Band power ERD analysis:** Alpha band ERD significant changes (5%): on almost all channels, but more pronounced in occipital region (O1, O2, Oz)	**Gaze fixations:** Alpha band ERD starts immediately as eye gaze is fixated (indication of visual processing during mind wandering)	

TABLE 2.3. CLASSIFIERS AND PERFORMANCE

Study	Labels	Classifier(s)	Performance metrics	Cross-validation	Results	Modalities comparison
(Asish et al., 2024)	2 classes: internal (ID) and external distraction (ED), based on self-assessment	kNN, R, 1D-CNN-LSTM, 2D-CNN	Accuracy, precision, recall, F1-score	9-k (cross-subject), leave-one-out (cross-session, 3 out of 4), 5-k (gender-based)	**Cross-subject:**<br>**-** RF: best accuracy (>83%), precision (ED 0.87%, ID 0.78%), recall (ED 0.77%, ID 0.91%), and F1-score (ED 0.82%, ID 0.84%)<br>**-** Overall kNN > 2D CNN > 1D CNN-LSTM<br>**Cross-session:**<br>- RF: best accuracy (>79% session 1, >77% session 4)<br>- Drop in accuracy for all the models (63-79%)<br>**Gender-based (RF only):**<br>- Gender-based grouping increased accuracy	**Cross-subject accuracy:**<br>- EEG+ET outperformed in all models (1D CNN 71.53%, 2D CNN 73.15%, kNN 81.74%, RF 83.60%)<br>- ET outperformed EEG in all models<br>**Gender-based (RF only):**<br>- EEG+ET (male group: 90% accuracy, 89% F1-score; female group: 91%, 91%; no grouping: 88%, 87%)<br>- EEG+ET outperforms single modality (all cases)<br>- ET outperforms EEG (all cases)

3. VIGILANCE

TABLE 3.1. EXPERIMENTATION DETAILS

Study	Participants	Condition(s)	Collected Data	EEG Channels	Type of ET signal
(Pan et al., 2024)	**N:** 60 (all male)<br>**Age:** avg 29.6 ± 2.7<br>**Other:** All professional drivers	Vigilance during monotonous driving task (dual-task paradigm: simulated driving as primary task, random stimulus detection as secondary task)	**Physiological measures:** EEG, ET, ECG<br>**Cognitive/performance-based measures:** RT and detection accuracy (hit rate)<br>**Subjective measures:** KSS, MWS	64-channel EEG (10-20 system): selected 9 channels (**occipital-parietal regions**) Pz, P1, P2, POz, PO3, PO4, Oz, O1, and O2; 1 reference point (M1)<br>Sampling rate: 1000 Hz	Head-mounted ET<br>Sampling rate: 60 Hz<br>**Eyelid Opening:** PERCLOS, blinks<br>**Eye Gaze:** fixations, saccades<br>**Pupil Dilation:** pupil diameter
(Larue et al., 2015)	**N:** 25 (18 female, 7 male)<br>**Age:** 18-49, avg 29.1 ± 8.3	Alertness during simulated highway-driving task	**Physiological measures:** EEG, ET, ECG, EDA<br>**Cognitive/performance-based measures:** driving performance	7 channels (10-20 system): O1, O2, T5, T6, P3, P4, F3 (**occipital, temporal, parietal and frontal regions**)<br>Sampling rate: 80 Hz	Remote ET<br>Sampling rate: 60 Hz<br>**Eyelid Opening:** eye closure and blinks
(Sengupta et al., 2017)	**N:** 30 (24 male, 6 female)<br>**Age:** avg 25.16 ± 5.78	Alertness during cognitive loading (test battery: SRT, VRT, LC, ART)	**Physiological measures:** EEG, ET, speech signal<br>**Cognitive/performance-based measures:** cognitive tasks to induce mental fatigue and detect alertness (VRT, ART, LC, SRT)	64-channel EEG (10-20 system); 2 reference points (earlobes); ground (forehead)<br>Sampling rate: 256 Hz	Remote ET<br>**Eye Gaze:** saccades
(Farha et al., 2022)	**N:** 9<br>**Age:** avg 24.5 ± 5.5	Vigilance during modified version of the SCWT	**Physiological measures:** EEG, ET	64-channel EEG: 2 reference points (M1 and M2 mastoids); ground (AFz)<br>Sampling rate: 500 Hz	Remote ET<br>Sampling rate: 500 Hz<br>**Eye Gaze:** fixations, saccades<br>**Eyelid Opening:** blinks<br>**Pupil Dilation:** pupil size
(Di Flumeri,	**N:** 14 (all male)<br>**Age:** avg 45 ± 7.5	Vigilance of ATCos using a highly automated HMI	**Physiological measures:** EEG, ET	15 channels (10-20 system): AF3, AF4, AF7, AF8, Fz,	Remote ET

Study	Participants	Condition(s)	Collected Data	EEG Channels	Type of ET signal
De Crescenzio, et al., 2019)	**Other:** Professional ATCos		**Cognitive/performance-based measures** **Subjective measures:** DSSQ, NASA-TLX	F3, F4, F7, F8, CP3, CP4, Pz, P3, P4, FPz (**frontal, prefrontal, centro-parietal regions**); 2 reference points (earlobes); ground (left mastoid) Sampling rate: 256 Hz	Sampling rate: 60 Hz **Eye Gaze:** fixations
(Bodala et al., 2016)	**N:** 12 (9 female, 3 male) **Age:** 20-31, avg 21.5 ± 1.68 **Other:** 4 additional subjects for control study	Vigilance during challenge integration: monitoring task as primary task and noisy visual stimulus as challenging stimulus (increases mental workload)	**Physiological measures:** EEG, ET, ECG, hEOG, vEOG **Cognitive/performance-based measures:** RT	64-channel EEG (10-20 system)	Remote ET Sampling rate: 500 Hz **Eye Gaze:** saccades, fixations **Eyelid Opening:** blinks
(Chua et al., 2012)	**N:** 24 (all male) **Age:** avg 25.9 ± 2.8 **Data problems:** ET only acquired for 15 participants	Sleep decrements in psychomotor vigilance during sleep deprivation: PVT task	**Physiological measures:** EEG, ET, ECG, EOG, body temperature **Cognitive/performance-based measures:** PVT (RT) **Subjective measures:** KSS, VAS	4-channels (10-20 system): Fz, Cz, Pz, Oz (**frontal, central, parietal, occipital regions**); 2 reference points (mastoids, A1 and A2) Sampling rate: 200 Hz	Remote ET Sampling rate: 120 Hz **Eyelid Opening**: PERCLOS, blinks

TABLE 3.2. IMPACT OF CONDITION ON FEATURES

Study	Condition(s)	EEG Features	ET Features	Other Features
(Pan et al., 2024)	Vigilance (caused by mental fatigue and mind-wandering)	Significant changes as vigilance decreases in 28/36 features: - Alpha power across all electrodes - Alpha/Beta across all electrodes - Beta power in P2, PO3, POz, O1, O2. - Non-linear feature sample entropy significant in P1, POz, PO4, Oz, O1, O2.	- Significant changes as vigilance decreases in PERCLOS, blink frequency and avg pupil diameter (e.g., higher PERCLOS and blink frequency)	**ECG:** significant changes in SDNN, LF, HF, and LF/HF ratios, indicating variations in heart rate variability **Behavioral performance:** worse when experiencing mind-wandering

Study	Condition(s)	EEG Features	ET Features	Other Features
(Larue et al., 2015)	Alertness	**Power values of sub-bands theta, delta, alpha and beta**<br>**-** (alpha + theta) / beta ratio above avg indicates reduced alertness	**Blink frequency and eye closure:** blink frequency more relevant	**ECG:** heart rate and inter-beat-interval<br>**EDA:** skin conductance level and nonspecific fluctuation rates (the rise-time and half-recovery)<br>**Performance:** car and environment variables (SD of the lane position, speed, steering wheel movement, and the time to line crossing on straight sections of the road only)
(Sengupta et al., 2017)	Alertness	**Power values of sub-band alpha:**<br>- Increase of alpha power with decrease in alertness<br>- Increase of alpha power with progression in number of stages	**PSV:**<br>- Decrease of PSV with decrease in alertness<br>- Decrease of PSV with progression in number of stages<br>- High positive correlation of PVT with SRT and VRT cognitive tasks (involvement of visual attention in these tasks)	**Speech signals: VUR**<br>- Decrease of VUR with decrease in alertness<br>- Decrease of VUR with progression in number of stages<br>**Cognitive tasks (VRT, ART, LC, SRT test):**<br>- VRT most significant (response to visual stimulus good marker of alertness)<br>- SRT less significant (repeated performance of task may improve due to practice, regardless of vigilance reduction)<br>- Strong correlation between the proposed metric (alpha + PSV + VUR) and subjective scores. Not good correlation with time to complete SRT and LC tasks.
(Farha et al., 2022)	Vigilance	**Power values of sub-bands delta, theta, alpha and beta** (n features = 62 channel x band)<br>- Deta and Alpha bands showed significant difference in frontal region (alertness vs vigilance decrement: change in vigilance level between begging and end)<br>- Theta and Beta bands showed significant difference in right temporal and occipital regions (alertness vs vigilance decrement: change in vigilance level between begging and end)	**Pupil size, fixation duration, saccade duration, saccade amplitude, blink duration, and saccade velocity**:<br>- Pupil size: highest importance in vigilance assessment<br>- Changes in eye tracking features: vigilance decrement had less spread and lower mean change than alertness	NA

Study	Condition(s)	EEG Features	ET Features	Other Features
		- Beta and Delta bands sensitive to changes in vigilance level (strong association with alertness)<br>- Delta the most sensitive (associated with deepest level of relaxation)<br>- Occipital and frontal regions sensitive to vigilance decrement (occipital region associated with visual activity processing, memory formation, distance, and depth perception; frontal region associated with high-level cognitive functions)<br>- Right central region in beta band the most sensitive to vigilance		
(Di Flumeri, De Crescenzio, et al., 2019)	Vigilance	**Power values of sub-bands, estimated by the IAF for bands directly correlated to vigilance: Theta and beta bands over frontal channels, Alpha band over centro-parietal channels, Theta over parietal channels**<br>EEG-based Vigilance index (VSCORE) computed with as-SWLDA algorithm:<br>- Significant increase of vigilance scores during "solution" scenario (controller more vigilant)<br>**-** Significant decrease of time spent by controller in Low vigilance condition during	**Eye gaze behavior: eye fixations per second and TTFF**<br>- Indicators of vigilance and attention<br>**-** Significant lower TTFT in "solution" scenario with respect to baseline scenario (incoming aircraft were recognized earlier and controllers more carefully processed information during fixations)<br>**-** TTFT also significantly lower during High vigilance with respect to Low vigilance (during "solution" scenario)<br>**-** Significantly less Fixations/second during "solution" scenario	Differences had similar trends in DSSQ and NASA-TLX results:<br>**Subjective measures of mind wandering (DSSQ):** During "solution" scenario, less task-related interference experienced and less task-unrelated thoughts (controllers less likely to be distracted by other matters than the task)<br>- Decrease in non-relevant thought in "solution" scenario<br>- Task-related thought equivalent between the two conditions (controllers equally thought about task-related things independently of their active involvement)<br>**Subjective measures of mental workload (NASA-TLX):** "Solution" scenario perceived as more demanding, less frustrating, and easier to achieve good performance in

Study	Condition(s)	EEG Features	ET Features	Other Features
		"solution" scenario (controller more vigilant)<br>**-** Decreasing tend of vigilance scores for baseline scenario	**-** Eye-tracking data show that neurophysiological reactions to lack of involvement also result in observable changes in controller behaviors	- Higher absolute mean values of Demands, Effort, and Overall Workload in the "solution" scenario<br>- Lower mean values of Frustration and Performance (= less dissatisfaction) in" solution" scenario
(Bodala et al., 2016)	Vigilance	**Power values of sub-bands related to vigilance decrement:**<br>- **Delta power** increases with vigilance decrement (negative correlation with vigilance levels)<br>- **Frontal midline theta and frontal theta to parietal alpha power ratio** decreases with vigilance decrement (positive correlation with vigilance levels)<br>- Reverse trends when challenge stimulus appears (suppression of delta power (fatigue inhibition); and frontal midline theta and frontal theta to parietal alpha power ratio increase (cortical arousal)) suggests vigilance enhancement due to challenge integration	**ET measures related to fixations, saccades and blinks**<br>**- Saccade amplitude and velocity** decrease with vigilance decrement (positive correlation with vigilance levels)<br>- **Blink rate** increases with vigilance decrement<br>- Reverse trends when challenge stimulus appears (increasing peaks in saccade amplitude and velocity, suppression of blink rate) suggests vigilance enhancement due to challenge integration	**RT mean:** lower for challenge integrated phase compared to initial monotonous phase without challenge<br>**Control group:**<br>- Saccade velocity is known to decrease with vigilance decrement<br>- Vigilance levels were higher for sessions with challenge stimulation: peaks in saccade velocity (challenge stimulation leads to increase in vigilance level)
(Chua et al., 2012)	Vigilance decrement during sleep deprivation	**Power values of sub-bands (Fz, Cz, Pz, Oz):**<br>**Delta band** had the highest correlation with PVT lapses:<br>- Stable activity during first 16 hours, sharp increase during the usual hours of sleep<br>- After peak at 22-26 hours, partial decrease of activity	**PERCLOS:**<br>- Positive correlation with PVT performance (PERCLOS increase with PVT impairment increase)<br>- Higher correlation with PVT lapses<br>**Eye blinks:**	**RT and PVT lapses:**<br>- Sharp increase during usual hours of sleep<br>- After 24 hours of wakefulness, partial improvement in PVT performance (presumably due to increased circadian drive for alertness)<br>**ECG:**<br>**-** RR-interval PSD similar to PVT lapses profile: increase during usual hours of sleep as subject became sleep-deprived (homeostatic drive to

Study	Condition(s)	EEG Features	ET Features	Other Features
		**Theta band:** Increased activity during usual hours of sleep, but remained elevated after 24 hours of wakefulness<br>**Alpha band**: Lowest during usual hours of sleep<br>**Beta band:** increased monotonically after usual hours of sleep	- Negative correlation with PVT performance (number of blinks decrease with PVT impairment increase)	sleep), decreased after 24 hours of wakefulness (circadian sleep-wake cycle)

TABLE 3.3. CLASSIFIERS AND PERFORMANCE

Study	Labels	Classifier(s)	Performance metrics	Cross-validation	Results	Modalities comparison
(Pan et al., 2024)	Vigilance levels caused by fatigue and mind wandering (based on KSS and MWS):<br>- Low vigilance caused by fatigue & mind wandering: LV_FM = KSS ≥ 5, MWS ≥ 5<br>- Low vigilance caused by fatigue: LV_F = KSS ≥ 5, MWS < 5<br>- Low vigilance caused by mind wandering: LV_M = KSS < 5, MWS ≥ 5<br>- High vigilance: HV = KSS < 5, MWS < 5	W-DCGAN (data augmentation) + CNN (feature fusion) + LSTM (vigilance estimation)	Accuracy, F1-score, precision, recall	5-k	**Average:**<br>- avg accuracy: 88.75%<br>- macro-F1 score: 0.89<br>- Overall accuracy drop of ~2% when training lacks diverse subject groups (individual differences impact generalization)<br>- Without data augmentation, model struggles to differentiate LV_F and LV_M<br>**For each vigilance status class:**<br>- accuracy LV_F: 82%<br>- accuracy LV_M: 85%<br>- accuracy LV_FM: 94%<br>- precision: 84.46 - 92.13%<br>- recall: 82.00 - 94.00%	Multi-modal performs better than using single modal features (improves vigilance estimation accuracy by 7%–15%):<br>**EEG:** macro-f1 0.83, accuracy 83.00% (best of single-modal: EEG features can effectively differentiate the decrease in vigilance caused by fatigue or mind-wandering)<br>**ECG:** macro-f1 0.76, accuracy 75.75% (most effective with LV_M than LV_F, ECG highly related to mental workload – mind wandering occurs when low mental workload)<br>**ET**: macro-f1 0.75, accuracy 75.00% (most effective with LV_F than LV_M, ET

Study	Labels	Classifier(s)	Performance metrics	Cross-validation	Results	Modalities comparison
					- F1-score: 0.87-0.92	highly sensitive indicator of fatigue)<br>**Fused features:** macro-f1 0.89, accuracy 88.75%
(Larue et al., 2015)	3 alertness levels: alert, reduced alertness, low alertness (based on ratio of low and high EEG frequency bands, (alpha + theta)/beta)	GLMMs, HMMs, NNs, SVM, CART	Sensitivity, specificity, AUC	Time-series (Rolling origin)	- GLMMs: overestimated alertness, not predictive of low alertness states (evolution of alertness might be too complex to be modeled linearly)<br>- HMMs: 70% sensitivity, 90% specificity for alertness state; not too accurate for lower alertness states (50% sensitivity, 30% specificity)<br>- SVM: 70% accuracy<br>- CART: 72% accuracy<br>- NN: 91% accuracy for low alertness state (only model capable of predicting low alertness state)	NA
(Farha et al., 2022)	2 vigilance levels: vigilance (alertness) and vigilance decrement (based on previous study behavioral results)	SVM	Accuracy, sensitivity, specificity	10-k	**EEG only:**<br>- Beta band: highest accuracy (92.0 ± 7.3%), sensitivity (91.7 ± 8.0%) and specificity 92.2 ± 7.1%)<br>- Delta band: second best accuracy (88.1 ± 8.5%)<br>**ET:**<br>- pupil size obtained highest accuracy among	**EEG (all regions) +ET** improves classification accuracy compared to single modality:<br>- Delta+ET: highest accuracy 96.8 ± 0.6%, sensitivity 97.3 ± 1.0%, specificity 96.3 ± 0.9%<br>- Theta+ET: accuracy 96.1 ± 1.1%, sensitivity 97.0 ± 1.2%, specificity 95.2 ± 1.3%

Study	Labels	Classifier(s)	Performance metrics	Cross-validation	Results	Modalities comparison
					individual features (71.8 ± 13.0%)<br>- All 6 features combined obtained highest accuracy (76.8 ± 8.4%)	- Alpha+ET: accuracy 96.3 ± 1.1%, sensitivity 97.0 ± 1.1%, specificity 95.7 ± 1.6%<br>- Beta+ET: accuracy 96.8 ± 1.1%, sensitivity 97.2 ± 1.0%, specificity 96.4 ± 1.2%<br>**EEG (brain regions) +ET**:<br>- Right central region (Beta band) + ET: highest accuracy (97.4 ± 1.3%)<br>- Right frontal region (Beta, Alpha bands) + ET: second highest accuracy (96.9 ± 1.1%)
(Chua et al., 2012)	Binary classification (Increase in PVT lapses above thresholds: >25%, >50%, >75%)	Threshold-based supervised learning (no classifier explicitly mentioned)	ROC curves, AUC, sensitivity, specificity	Leave-one-subject-out	**HRV (RR-interval PSD):** AUC = 0.87 (±0.02), Sensitivity = 76%, Specificity = 89% at 25% threshold<br>**PERCLOS:** AUC = 0.89 (±0.02), Sensitivity = 78%, Specificity = 88% at 25% threshold<br>**Frontal EEG (delta power):** AUC = 0.82 (±0.02)<br>**Self-reported Sleepiness (VAS):** AUC = 0.83 (±0.02)	- PERCLOS and HRV outperformed EEG and self-reported sleepiness<br>- PERCLOS was the strongest predictor overall, with HRV slightly worse at the 50% threshold<br>- No fusion of metrics performance reported

4. DROWSINESS & SLEEP

TABLE 4.1. EXPERIMENTATION DETAILS

Study	Participants	Condition(s)	Collected Data	EEG Channels	Type of ET signal
(Zaky et al., 2023)	**N:** 20 (10 male, 10 female)<br>**Age:** 21-45, avg 29.3<br>**Data problems:** Only 10 participants analyzed (exclusion reasons: 4 had no microsleeps, 2 few microsleeps, 2 missing EEG data, 1 poor signal-to-noise-ratio, 1 low duration of microsleep)	Microsleeps during 2D CVT task	**Physiological measures:** EEG, ET, vEOG, fMRI<br>**Cognitive/performance-based measures:** tracking performance	64-channel EEG (10-20 system): reference point between Cz and Pz; ground (near Pz)<br>Sampling rate: 10000 Hz	Remote ET<br>**Eye Gaze:** gaze position<br>**Eyelid Opening:** blinks, eye closure
(Zaky et al., 2021)	**N:** 14<br>**Data problems:** data corruption of 3 participants	Microsleeps during 2D CVT task	**Physiological measures:** EEG, ET<br>**Cognitive/performance-based measures:** tracking performance	64-channel EEG (10-20 system): 1 reference point (close to Cz); ground (close to Fz)<br>Sampling rate: 10000 Hz	Remote ET<br>Sampling rate: 25 Hz<br>**Eye Gaze:** gaze position<br>**Eyelid Opening:** blinks, eye closure
(Poudel et al., 2012)	**N:** 20 (10 male, 10 female)<br>**Age:** 21-45, avg 29.3	Drowsiness during CVT task	**Physiological measures:** EEG, ET, fMRI, vEOG, ECG, oximetry<br>**Cognitive/performance-based measures:** response behavior	64-channel EEG (10-20 system): 1 reference point (close to Cz); ground (close to Fz)<br>Sampling rate: 10000 Hz	Remote ET<br>Sampling rate: 25 Hz<br>**Eye Gaze:** gaze position<br>**Eyelid Opening**: PERCLOS
(Poudel et al., 2010)	**N:** 20 (10 male, 10 female)<br>**Age:** 20-45, avg 29.3	Tonic drowsiness and microsleeps during 2D pursuit-tracking task	**Physiological measures:** EEG, ET, vEOG<br>**Cognitive/performance-based measures:** visuomotor response	64-channel EEG	Remote ET<br>Sampling rate: 25 Hz<br>**Eye Gaze:** gaze position<br>**Eyelid Opening:** eye closure

Study	Participants	Condition(s)	Collected Data	EEG Channels	Type of ET signal
(Arsen'ev et al., 2015)	**N:** 19<br>**Age:** 21-30	Drowsiness (decrease in level of arousal) and microsleeps during visuomotor coordination task	**Physiological measures:** EEG, ET, EOG<br>**Cognitive/performance-based measures:** visuomotor coordination parameters	2 channels: C3, C4 (**central region**); 2 reference points (mastoids)<br>Sampling rate: 200 Hz	Remote ET<br>Sampling rate: 120 Hz<br>**Eye Gaze:** saccades, gaze position
(Zandi et al., 2019)	**N:** 53 (37 male, 16 female)<br>**Age:** 19-61, avg 38.1 ± 11.6	Drowsiness during driving	**Physiological measures:** EEG, ET	14 channels<br>Sampling rate: 128 Hz	Remote ET<br>Sampling rate: 60 Hz<br>**Eye Gaze:** gaze position, fixations, saccades<br>**Eyelid Opening:** blinks, eyelid opening<br>**Pupil Dilation:** pupil diameter

TABLE 4.2. IMPACT OF CONDITION ON FEATURES

Study	Condition(s)	EEG Features	ET Features	Other Features
(Zaky et al., 2023)	Microsleeps	**Power values of sub-bands:**<br>- Increased activity between pre and start of microsleeps in theta and alpha bands (theta increase in frontal, parietal, temporal, and occipital lobes; alpha increase in parietal and occipital lobes)<br>- Increased activity between start and end of microsleeps in delta, beta and gamma bands (delta increases in activity in the frontal, parietal, temporal, and occipital lobes; beta increase in frontal, parietal, and temporal lobes; gamma increases in frontal, parietal and occipital lobes)	- Flat/incoherent gaze tracking (0.5 – 15s) classified as microsleeps<br>- Complete or partial phasic eye closure (except for blinks) classified as microsleeps	**Behavioral data (2D tracking performance and eye video)**: clear behavioral indicators of drowsiness/sleepiness classified as microsleeps

Study	Condition(s)	EEG Features	ET Features	Other Features
		- Decreased activity between end and post of microsleeps in alpha and delta bands (alpha decrease in the left frontal, parietal, temporal, and occipital lobes, as well as the insular cortex; delta decrease in left parietal and occipital lobes)		
(Zaky et al., 2021)	Microsleeps	**Power values of sub-bands:**<br>- Microsleeps represented by increase in delta, theta and alpha activities (frontal lobe), and beta activity (parietal and occipital lobes)<br>- No significant changes in gamma band, and no significant decreases in any band<br>- Delta band: maximum increases in bilateral frontal pole and superior frontal gyrus regions<br>- Theta band: maximum increases in bilateral superior frontal gyrus and middle frontal gyrus regions<br>- Alpha band: maximum increases in bilateral inferior frontal gyrus regions<br>- Beta band: maximum increases in right cuneal and bilateral precuneus cortices	- Flat/incoherent gaze tracking (0.5 – 15s) classified as microsleeps<br>- Complete or partial phasic eye closure (except for blinks) classified as microsleeps	NA
(Poudel et al., 2012)	Drowsiness/ microsleeps	**Power values of sub-bands (avg theta activity):**<br>- Relative power in theta band at Pz electrode used as an EEG estimate of drowsiness<br>- Participants who had EEG electrode impedance <15Ω showed moderate correlation between minute-to-minute changes in performance and theta power at Pz	Microsleeps identified as flat tracking (zero response speed during 0.5 to 15 s) accompanied by behavioral signs of drowsiness and full or partial (>80%) slow-eye-closures<br>**PERCLOS:** 80% eye-closure in a minute used as an ET estimate of drowsiness	**Average tracking error**<br>- Minute-to-minute changes in performance correlated moderately with PERCLOS<br>**fMRI**<br>- Microsleeps associated with decreased neural activity in the arousal-related brain regions including the thalamus, midbrain, and posterior cingulate cortex, but associated with increased activity in the frontoparietal, insular, para-hippocampal, and temporo-occipital cortices increases

Study	Condition(s)	EEG Features	ET Features	Other Features
				- Theta activity in the postcentral EEG correlated positively with BOLD signal in the bilateral thalamus, basal forebrain, prefrontal, posterior cingulate, posterior parietal, and visual cortices<br>- Rapid bilateral decrease in thalamic activity at the onset of microsleeps with the amplitude of the decrease modulated by the duration of the microsleep
(Poudel et al., 2010)	**Microsleeps**	**Power values of sub-bands (alpha and theta)**<br>- Positive correlation of theta activity with tracking error (most strong at Pz); reduced correlation with exclusion of microsleep events (microsleeps contribute to fluctuations in performance and theta activity during extended visuomotor task – deterioration of performance and increase theta activity at Pz)<br>- Inconsistent correlation of alpha activity and tracking error (with and without microsleeps)	Microsleeps identified as flat tracking (0.5-15s) accompanied by slow-eye-closure (>80%)<br>- 70% of eye closures during microsleeps longer than 0.3s	**Visuomotor performance (tracking error and response speed)**<br>- Tracking error had large fluctuations for participants experiencing drowsiness and microsleeps<br>- Constant tracking error for alert participants
(Arsen'ev et al., 2015)	Drowsiness	**Power values of sub-bands**<br>- Calm walking: clear beta rhythm; periodic low-amplitude alpha rhythm; occasional theta and delta<br>- Low arousal state: clear alpha rhythm; suppression of alpha rhythm on the background of low-amplitude theta and delta waves; weak beta rhythm<br>- Microsleeps: strong theta and/or delta rhythm; some sleep spindles	**Gaze-related visuomotor coordination parameters (latent periods of gaze, deviation of gaze from target)**<br>- Low arousal state: defocusing; significant increases in latent periods of saccades and variability of gaze from center of target<br>- Microsleeps: defocusing on gaze and tacking; absent saccadic movements	**EOG:**<br>- Calm walking: blinks were seen<br>- Low arousal state: changes in blinking (for some participants there was a frequency increase, other had longer-lasting blinking) – early sign of increased fatigue and drowsiness<br>- Microsleeps: frequent eyes closing or eyeballs rolling up<br>**Head position and facial expression:**<br>- Low arousal state: change in position and expression

Study	Condition(s)	EEG Features	ET Features	Other Features
			- Highly sensitive to changes in level of arousal (decreases in level of arousal accompanied by increases in latent periods of saccadic eye movements and variability in deviations of gaze from the center of the target)<br>- Positive correlation between latent periods of mouse key pressing in response to appearance of novel stimulus and mean square deviation in distance between gaze and target center 5 sec before the stimulus (slowing of the reaction as the level of arousal decreased occurred on the background of growth in the amplitude of gaze oscillations during target tracking)<br>- Significant increases in latent periods of saccades, onset of cursor movement, and pressing of the mouse button 2–3 min before the moment at which the expert identified the low-arousal state (this allows prediction of low-arousal state before reflection in participant's activity)	- Microsleeps: significant head position change (lean towards shoulders, falling onto chest)<br>**Task performance-related visuomotor coordination parameters (mouse cursor displacement, latent periods of mouse button click in response to stimuli, deviation of mouse cursor from target center)**<br>- Low arousal state: impairment (increased RT, occasional errors – increases in latent periods of cursor movement initiation, mouse key pressing, and cursor deviations from center of target)<br>- Microsleeps: hand stopped or moved on a random trajectory; cease in performance<br>- Highly sensitive to changes in level of arousal (decreases in level of arousal accompanied by increases in mouse cursor movements and pressing the mouse button, as well as variability in deviations of the mouse cursor from the center of the target)
(Zandi et al., 2019)	Drowsiness	**Power values of sub-bands (in 7 channels AF3, AF4, F7, F8, F3, F4, FC5, FC6, T7, T8, P7, P8, O1, O2)**<br>- Positive correlation of beta/alpha and alpha/(delta + theta) ratios and alertness (higher the power ratios, higher the level of alertness)<br>- When the ratios decrease, drowsiness	**Features of general gaze (median, SD, scanpath, velocity ratio, entropy, similarity index), fixations (duration, frequency, percentage, scanpath, velocity, similarity index), saccades (duration, frequency, percentage, scanpath, velocity, similarity index), blinks (duration, frequency, percentage),**	

Study	Condition(s)	EEG Features	ET Features	Other Features
			**pupil diameter (mean, SD), eyelid opening (mean, SD)**<br>High correspondence between the extracted ET features and EEG as a physiological measure of vigilance.<br>- top 10 features: Gaze SD (heading), Gaze median (pitch), Saccade percentage, Saccade scanpath (heading), Saccade scanpath (pitch), Saccade velocity (heading), Blinking percentage, Pupil diameter mean, Pupil diameter SD, Eyelid opening mean	

TABLE 4.3. CLASSIFIERS AND PERFORMANCE

Study	Labels	Classifier(s)	Performance metrics	Cross-validation	Results	Modalities comparison
(Zandi et al., 2019)	Binary classification: alert, drowsy (based on EEG data)	RF, non-linear SVM	sensitivity (for drowsy state), specificity (for alert state), accuracy (for both classes together)	5-k	- Performance improves by increasing epoch length up to 30s<br>- RF outperforms non-linear SVM on all measures of performance and shows higher robustness against epoch length<br><br>Mean ± SD of accuracy (sensitivity–specificity):<br>- RF: 89.84 ± 1.05% (89.35 ± 0.87% to 90.32 ± 1.31%)<br>- Non-linear SVM: 81.24 ± 1.76% (80.42 ± 1.84% to 82.06 ± 1.86%)	NA

5. MENTAL WORKLOAD

TABLE 5.1. EXPERIMENTATION DETAILS

Study	Participants	Condition(s)	Collected Data	EEG Channels	Type of ET signal
(Borys, Tokovarov, et al., 2017)	**N:** 20 (all male)<br>**Age:** avg 22.8 ± 0.83<br>**Data problems:** EEG of 7 participants (data quality)	Cognitive load during arithmetic tasks with different levels of difficulty	**Physiological measures:** EEG, ET	21-channel EEG (10-20 system): C3, Cz, C4, P3, F3, F4, F7, F8, Fz, O1 (**central, parietal, frontal, occipital regions**); 2 reference points (A1, A2); ground electrode (frontal lobe center)<br>Sampling rate: 500 Hz	Head-mounted ET<br>Sampling rate: 60 Hz<br>**Eye Gaze:** fixations, saccades<br>**Eyelid Opening:** blinks<br>**Pupil Dilation:** pupil diameter
(Borys, Plechawska-Wójcik, et al., 2017)	**N:** 20 (all male)<br>**Age:** avg 22.8 ± 0.83<br>**Data problems:** EEG of 7 participants (data quality)	Mental workload during arithmetic tasks	**Physiological measures:** EEG, ET	21-channel EEG (10-20 system)<br>Sampling rate: 500 Hz	Remote ET<br>Sampling rate: 30 Hz<br>**Eye Gaze:** fixations, saccades<br>**Eyelid Opening:** blinks<br>**Pupil Dilation:** pupil diameter
(Kujur et al., 2022)	**N:**13 (7 male, 6 female)<br>**Age:** avg 23.15 ± 1.3	Mental workload during numerical estimation tasks, with induced distraction	**Physiological measures:** EEG, ET	14-channel EEG (10-20 system)<br>Sampling rate: 128 Hz	Remote ET<br>Sampling rate: 120 Hz<br>**Pupil Dilation:** pupil dilation
(Guo et al., 2024)	**N:** 20 (all male)<br>**Age:** avg 22.46<br>**Other:** Special vehicle crews (over 100 hours of operational training)	Mental workload during a multi-phase operational task (search, strike, observe, report)	**Physiological measures:** EEG, ET<br>**Cognitive/performance-based measures:** performance data<br>**Subjective measures:** NASA-TLX	32 channels (10-20 system)<br>Sampling rate: 500 Hz	Remote ET<br>Sampling rate: 60 Hz<br>**Eyelid Opening:** eyelid opening<br>**Pupil Dilation:** pupil diameter

Study	Participants	Condition(s)	Collected Data	EEG Channels	Type of ET signal
(Singh et al., 2021)	**N:** 14 (8 male, 6 female)<br>**Age:** avg 24.4 ± 1.95<br>**Data problems:** ET of 1 participant (technical issues)	Mental workload during MUM-T for Pilot-UAV Teaming Applications	**Physiological measures:** EEG, ET, EOG, ECG<br>**Cognitive/performance-based measures:** behavioral (tasks performance scores)<br>**Subjective measures:** ISA	32-channel (10-20 system)<br>Sampling rate: 2048 Hz	Head-mounted ET<br>Sampling rate: 100 Hz (raw data), 15 Hz (image data)<br>**Eye Gaze:** fixations<br>**Eyelid Opening:** blinks<br>**Pupil Dilation:** pupil dilation
(Diaz-Piedra et al., 2019)	**N**: 15 (all male)<br>**Age:** avg 39 ± 5.6<br>**Data problems:** 1 participant excluded, and ET of another participant (recording system failure)<br>**Other:** professional military fighter pilots	Mental workload during in-flight emergencies (task load variations as a function of flight complexity)	**Physiological measures:** EEG, ET<br>**Cognitive/performance-based measures:** performance (number of errors)<br>**Subjective measures:** SSS, NASA-TLX	5 channels (10-20 system): F3, F4, C3, C4, Cz (**frontal and central regions**); 2 reference points (mastoids A1, A2); ground reference (FPz); Cz (internal reference)<br>Sampling rate: 256 Hz	Head-mounted ET<br>Sampling rate: 30 Hz<br>**Eye Gaze:** gaze entropy
(Matthews et al., 2015)	**N:** 150 (85 male, 65 female)<br>**Age:** avg 19.57 ± 3.46<br>**Data problems:** up to 6 participants (depending on the sensor)	Mental workload during 4 military monitoring tasks (simulation of unmanned ground vehicle operation)	**Physiological measures:** EEG, ET, ECG, TDS, fNIR<br>**Subjective measures:** NASA-TLX	9 channels: Fz, F3, F4, Cz, C3, C4, Pz, P3, and P4 (**frontal, central, parietal regions**); reference points (mastoids)<br>Sampling rate: 256 Hz	Remote ET<br>Sampling rate: 60 Hz<br>**Eye Gaze:** fixations<br>**Pupil Dilation:** pupillometry-based ICA
(Di Flumeri, Borghini, et al., 2019)	**N:** 8 (all male)<br>**Age:** avg 24.9 ± 1.7	Mental workload during driving	**Physiological measures:** EEG, ET	12 channels (10-20 system): FPz, AF3, AF4, F3, Fz, F4, P3, P7, Pz, P4, P8, and POz (**frontal, anterior frontal, parietal, parietal-occipital regions**); 2 reference	Head-mounted ET<br>Sampling rate: 30 Hz<br>**Eye Gaze:** fixations

Study	Participants	Condition(s)	Collected Data	EEG Channels	Type of ET signal
				points (earlobes); ground (Cz)<br>Sampling rate: 256 Hz	
(Di Flumeri et al., 2018)	**N:** 20 (all male)<br>**Age:** avg 24.9 ± 1.8<br>**Data problems:** EEG of 4 participants, ET of 2 (out of 10)	Mental workload during driving (different traffic conditions and road types)	**Physiological measures:** EEG, ET<br>**Cognitive/performance-based measures:** car parameters, videos around the car<br>**Subjective measures:** NASA-TLX	12-channel EEG (10-20 system): FPz, AF3, AF4, F3, Fz, F4, P3, P7, Pz, P4, P8, and POz (**frontal, anterior frontal, parietal, parietal-occipital regions**); 2 reference points (earlobes); ground (Cz)<br>Sampling rate: 256 Hz	Head-mounted ET<br>Sampling rate: 30 Hz<br>**Eye Gaze:** fixations
(Yang et al., 2020)	**N:** 32 (16 male, 16 female)<br>**Age:** 22-30, avg 24.7 ± 1.9	Mental workload and behavior during driving	**Physiological measures:** EEG, ET<br>**Cognitive/performance-based measures:** driving behavior	32-channel EEG: Fz, F3, F4, F7, F8, FP1, FP2 (for analysis, **pre-frontal cortex area**)<br>Sampling rate: 500 Hz	Head-mounted ET<br>Sampling rate: 60 Hz<br>**Eye Gaze**: fixations, saccades
(Angkan et al., 2024)	**N:** 23 (17 female, 6 male)<br>**Age:** avg 26.9	Cognitive load during driving	**Physiological measures:** EEG, ET, EDA, ECG<br>**Subjective measures:** cognitive load self-assessment	4-channel EEG headband (10-20): AF7, AF8, TP9, TP10 (**frontal and temporal regions**); reference electrode (FPz)<br>Sampling rate: 256Hz	Head-mounted ET<br>Sampling rate: 50 Hz<br>**Eye Gaze:** saccades, fixations, gaze (velocity, acceleration, direction)<br>**Eyelid Opening:** blinks<br>**Pupil Dilation:** pupil diameter
(Eniyandunmo et al., 2024)	**N:** 52 (45 male, 7 female)<br>**Age:** 19-37, avg 26.12 ± 4.53	Mental workload during driving (five-task driving scenario)	**Physiological measures:** EEG, ET, EDA, ECG, facial EMG	14 electrodes: AF3, AF4, F3, F4, F7, F8, FC5, FC6, T7, T8, P7, P8, O1, and O2 (**anterior**	Head-mounted ET<br>Sampling rate: 256 Hz

Study	Participants	Condition(s)	Collected Data	EEG Channels	Type of ET signal
	**Data problems:** 11 participants		**Subjective measures:** modified Bedford scale (subjective mental workload rating)	**frontal, frontal, frontal-central, temporal, parietal, occipital regions**)<br>Sampling rate: 128 Hz	Pupil Dilation: pupil diameter
(Shafiei et al., 2024)	**N:** 26 (18 male, 8 female)<br>**Age:** avg 36 ± 12	Mental workload during surgical tasks with different difficulty levels	**Physiological measures:** EEG, ET	124-channel EEG: 116 channels used (F8, POz, AF4, AF8, F6, FC3, M1, and M2 excluded due to poor signal quality); 1 reference point (Cz)<br>Sampling rate: 500 Hz	Head-mounted ET<br>Sampling rate: 50 Hz<br>**Eye Gaze:** fixations, saccades, gaze direction, pupil trajectory length<br>**Pupil Dilation**: pupil diameter
(Barragan et al., 2022)	**N:** 8 (Dataset 1, EEG only), 10 (Dataset 2, EEG + ET)	Cognitive workload during robotic-assisted surgical tasks	**Physiological measures:** EEG, ET	32-channel EEG: 1 reference point (right earlobe); ground (AFz)<br>Sampling rate: 250 Hz	Head-mounted ET<br>Sampling rate: 60 Hz<br>**Eye Gaze:** fixations, scan path length, nearest neighbor index of fixations<br>**Pupil Dilation:** pupil diameter
(Orlandi & Brooks, 2018)	**N:** 10 (all male)<br>**Other:** marine pilots	Mental workload during different ship-handling conditions while berthing ships in a simulator	**Physiological measures:** EEG, ET, ECG<br>**Subjective measures:** NASA-TLX, Likert scale	14-channels EEG: AF3, F7, F3, FC5, T7, P7, O1, O2, P8, T8, FC6, F4, F8, AF4 (**anterior frontal, frontal, temporal, parietal, occipital, frontal-central regions**)<br>Sampling rate: 128 Hz	Head-mounted ET<br>Sampling rate: 30 Hz<br>**Pupil Dilation:** pupil diameter
(Iqbal et al., 2024)	**N:** 8	Mental workload of control room operators	**Physiological measures:** EEG, ET	Single channel (**prefrontal region**)<br>Sampling rate: 512 Hz	Sampling rate: 120 Hz

Study	Participants	Condition(s)	Collected Data	EEG Channels	Type of ET signal
					**Eye Gaze:** fixations<br>**Pupil Dilation:** pupil diameter
(Jimenez-Molina et al., 2018)	**N:** 61 (42 male, 19 female)<br>**Age:** 19-35, avg 23.8 ± 3.2<br>**Data problems:** 14 participants	Mental workload during web browsing task	**Physiological measures:** EEG, ET, EDA, PPG, temperature<br>**Cognitive/performance-based measures:** IAA (parameter to assign cognitive workload labels, IAA decreases with increase of expertise, highlights decrease in cognitive workload)	14 EEG channels<br>Sampling rate: 128 Hz	Remote ET<br>Sampling rate: 100 Hz<br>**Pupil Dilation:** pupil diameter
(Lobo et al., 2016)	**N:** 21	Cognitive workload during dual task paradigm: primary task (visual search task), secondary interfering task (syntactic transformation task).	**Physiological measures:** EEG, ET	8-channel EEG: **prefrontal, frontal and parietal lobes**	Remote ET<br>**Eyelid Opening:** eye closure (%)<br>**Pupil Dilatation:** pupil diameter
(Mark et al., 2024)	**N:** 23 (16 female, 7 male)<br>**Age:** 18-48, avg 23	Mental workload during six cognitive tasks (working memory, vigilance, risk assessment, shifting attention, situation awareness, and inhibitory control)	**Physiological measures:** EEG, ET, EOG, ECG, PPG, fNIRS	72-channel EEG: 32 channels used<br>Sampling rate: 500 Hz	Remote ET<br>Sampling rate: 60 Hz<br>**Eye Gaze:** fixations, saccades<br>**Pupil Dilatation:** pupil diameter
(Aksu et al., 2024)	**N:** 15 (8 male, 7 female)<br>**Age:** 19-25, avg 21.6	Mental workload during n-back tasks	**Physiological measures:** EEG, ET<br>**Subjective measures:** NASA-TLX	14-channel EEG (10-20 system)<br>Sampling rate: 128 Hz	Remote ET<br>Sampling rate: 60 Hz<br>**Eye Gaze:** fixations, saccades<br>**Eyelid Opening:** blinks

Study	Participants	Condition(s)	Collected Data	EEG Channels	Type of ET signal
					**Pupil Dilation:** pupil diameter
(Planke et al., 2021)	**(Session 1):**<br>**N:** 17 (10 female, 7 male)<br>**Age:** avg 29 ± 8.2<br><br>**(Session 2):**<br>**N:** 12 (7 male, 5 female)<br>**Age:** Avg 31 ± 9.3	Mental workload during MATB scenario	**Physiological measures:** EEG, ET, ECG<br>**Cognitive/performance-based measures:** number of mouse clicks	16-channel EEG (10-20 system): F4, AFz, F3, FCz, C3, C4, CPz, T7, T8, P3, POz, P4, P7, P8, O1 and O2 (**frontal, anterior frontal, temporal, central, central-parietal, occipital regions**)	Remote ET<br>Sampling rate: 60 Hz<br>**Eye Gaze:** entropy, dwell time<br>**Eyelid Opening:** blinks<br>**Pupil Dilation:** pupil diameter
(John et al., 2022)	**N:** 24 (17 male, 7 female)<br>**Age:** avg 25 ± 5.17	Mental workload during tacking and collision prediction tasks with 3 levels of difficulty	**Physiological measures:** EEG, ET, ECG<br>**Cognitive/performance-based measures:** tracking accuracy (tracking task); time before collision and collision miss proportion rate (collision prediction task)	64-channel EEG	Head-mounted ET<br>Sampling rate: 200 Hz (eyes), 30 Hz (field of view)<br>**Pupil Dilation:** pupil size<br>**Eyelid Opening:** blinks

TABLE 5.2. IMPACT OF CONDITION ON FEATURES

Study	Condition(s)	EEG Features	ET Features	Other Features
(Borys, Tokovarov, et al., 2017)	Cognitive load	**Power values of sub-bands (Cz, F3, F4, P3, P4)**<br>- Beta 2 waveform similar trend to increasing errors and RT<br>- Alpha waveform inversely correlated with number of errors<br>- No significant relationship between EEG features and cognitive workload (difficulty degree)	**Fixations (number, mean duration, SD of duration and max), saccades (number, mean duration, SD of duration, mean and max amplitude), blinks (number, mean duration, total duration known also as closure duration, duration skewness), pupil response (mean, maximum, SD of pupil diameter)**<br>- Mean moderate positive correlation of mean and max amplitude of saccade and cognitive workload	**Cognitive measure (Number of errors and RT):**<br>- Increased number of errors and RT (increasing difficulty of tasks) meant increasing cognitive workload and mental effort – very strong correlation of interval difficulty degree and number of errors, moderate correlation with RT<br>- Correlation between cognitive measure and EEG and ET

Study	Condition(s)	EEG Features	ET Features	Other Features
			- Weak but significant correlation of mean, SD, max fixation duration and cognitive load (inverse relationship)<br>- Max blink duration significant correlation with cognitive load<br>- Eye movement and pupil dilation measures: good indicators of cognitive workload	features: in each interval, different features were significant (no distinctive best indicator)
(Borys, Plechawska-Wójcik, et al., 2017)	Mental workload	**Power values of sub-bands (Cz, F3, F4, P3, P4)**<br>Dataset 2<br>- Only correlation with Cz alpha feature found significant, but weak	**Fixations (number, mean, median, SD, max of duration), saccades (number, mean, median, SD, maximum of duration), saccade amplitude (mean, median, maximum), saccade acceleration (mean, median, max), pupil diameter (mean, median, SD, max, skewness, kurtosis), blinks (number, mean, median, SD, maximum, total of duration)**<br>Dataset 1<br>- Strongest correlation for features based on blinks duration<br>- Many correlations between ET features (eye movements and features are connected)<br>Dataset 2<br>- Moderate correlation for 2 features based on fixation duration and for 2 based on blink duration	NA
(Kujur et al., 2022)	Mental workload	**Power values of sub-bands, motivation index (FAA)**<br>- Increased beta power became as cognitive task load increased (most active power in frontal regions)<br>- Frontal theta increased as cognitive task load increased	**Pupil dilation**<br>- Right pupil dilation increase with cognitive load increase<br>- Pupil (right), pupil (left) amongst the top ten important features	NA

Study	Condition(s)	EEG Features	ET Features	Other Features
		- Average Frontal Low Beta, Average PSD Frontal theta, Motivation index FAA, Average PSD temporal low beta, Average Occipital low beta, Average Occipital theta, Average temporal low beta, Average temporal alpha amongst the top ten important features		
(Guo et al., 2024)	Mental workload (based on performance)	**Power values of sub-bands (Fp1, Fp2, F7, F8, F3, F4):**<br>- No significant increases in delta power (eye movement artifacts did not substantially affect)<br>- Theta power (at Fp1): differences between observe and search stages and between the search and report stages; elevated during search stage (higher mental workload task)<br>- Beta power (at Fp1 and F3): significantly higher during search stage compared to observe, strike, and report stages<br>- Differential patterns only observed at Fp1 and F3 (task-specific neural activity – prefrontal area)<br>- Increased cognitive load and attentional demands during search stage (reflected in elevated theta and beta band activity): searching requires more cognitive engagement than the other stages (hierarchy of mental workload: Search > Strike > Observe/Report (low workload))	**Pupil diameter and eyelid opening:**<br>- Higher during search stage (higher mental workload task)<br>- No differences between strike, observe and report stages	NA
(Singh et al., 2021)	Mental workload	**Power values of sub-bands and engagement index:**	**Blink latency, number of fixations, fixation duration, and pupil dilation**	**ECG (HR and HRV):** significant increase in HR and

Study	Condition(s)	EEG Features	ET Features	Other Features
		- 20 electrodes with highest magnitude difference between high and low mental workload: Fp1, AF3, F7, F3, FC5, T7, CP5, P7, PO3, O1, Oz, O2, PO4, P8, T8, FC6, F4, F8, AF4, and Fp2<br>- General increase in power with an increase in workload, modulated by interactions with electrode site and band<br>- Beta and gamma powers significantly increase with increase in workload in frontocentral, temporal and occipital sites (Fp2, FC5, FC6, T7, T8, CP5, P8, O1, Oz, and O2)<br>- Decrease in alpha power with increase in workload at parietal sites (did not reach significance)<br>- Increase in engagement index with an increase in workload (significantly at FC5, FC6, T7, T8, CP5, and P8)	- Fixations number significantly increased with an increase in workload<br>- Average fixation duration significantly decrease with an increase in workload (the more demanding the scenario was on attentional resources that had to be allocated to several tasks)<br>- Pupil dilation significantly increased with an increase in workload<br>- Blink latency was not significantly impacted by load<br>- Significant interaction effect on the total fixation duration on specific AOIs: more time spent on AOIs ("middle screen of the plane flying simulator" and "U-track sub-part for analyzing the movement of plane to avoid red zones") during high workload condition	significant decrease in HRV with an increase in workload<br>**Behavioral data:** performance (pilot flying and ATC commands scores) significantly decrease and RT increase with an increase in workload<br>**Subjective data (ISA questionnaire):** reported workload significantly increased with an increase in workload
(Diaz-Piedra et al., 2019)	Cognitive load	**Power values of sub-bands (F3, F4, C3, C4)**<br>- Averaged power values for frontal and central regions: higher frontal and central theta power during emergency flights than recognition flights (relationship between EEG theta power and cognitive effort)<br>- In both emergency or recognition flights, there were no differences between medium and high complexity flights in theta EEG power	**Gaze entropy (Shannon's)**<br>- Lower gaze entropy during emergency flights than recognition flights (less random exploration pattern during more complex flights, pilots used a more systematic visual scanning pattern)<br>- In aviation setting there are highly standardized procedures to solve emergencies: pilots might use nondeterministic visual patterns during error-free states (recognition flight) and change scanning behavior when detecting emergencies	**Performance (measured by flight instructor)**<br>- Performance lowered as flight complexity increased (significant linear trend)<br>- Performance during recognition flights significantly higher than to emergency flights (increased number of errors for emergency flights)<br>**Subjective ratings of task load (NASA-TLX)**<br>- Subjective ratings differed depending on flight complexity (significant linear trend)

Study	Condition(s)	EEG Features	ET Features	Other Features
				- Higher levels of perceived task load after performing emergency flights compared to recognition flights<br>- No differences between medium and high complexity flights in reported scores
(Matthews et al., 2015)	Mental workload	**Power values of sub-bands (frontal sites F3, F4, Fz) and Task Load Index:**<br>- **EEG theta:** discriminated single-task change detection and threat detection (lower workload), but not single-task from dual-tasks<br>- **EEG beta:** highest workload for single-task threat detection<br>- **EEG alpha:** insensitivity (maybe due to short duration of tasks, metric sensitive to task demands during sustained attention)<br>- **Task Load index:** largest effect size among EEG measures. Discriminated dual-task from single-task, but not the two single tasks	**Fixation duration:** largest effect size. Discriminated dual-task from single-task, but not the two single tasks (change detection vs. threat detection)<br>**ICA:** discriminated single-task change detection and threat detection (lower workload), but not single-task from dual-tasks	**ECG (mean IBI, HRV):** HRV discriminated single-task change detection and threat detection (lower workload), but not single-task from dual-tasks<br>**TDS (CBFV during performance):** insensitivity (maybe due to short duration of tasks, metric sensitive to task demands during sustained attention)<br>**fNIRS (Oxygen saturation during performance):** (to some extent) discriminated single-task change detection and threat detection (lower workload), but not single-task from dual-tasks<br>**Subjective workload (NASA-TLX):** highest in the two dual-task conditions and lower for single-task threat detection than for change detection: sensitive to both task type and dual tasking<br>**Performance data:** confirms that threat detection was the more difficult of the two tasks and dual tasking was associated with performance deficits on both tasks

Study	Condition(s)	EEG Features	ET Features	Other Features
(Di Flumeri, Borghini, et al., 2019)	Mental workload	**Power values of sub-bands, IAF: theta rhythms [IAF − 6 ÷ IAF− 2] over the frontal sites and the alpha rhythms [IAF − 2 ÷ IAF+ 2] over the parietal sites**<br>- Theta and alpha rhythms used to compute the WL index (based on as-SWLDA classifier)<br>- WL indexes increased with task complexity (significantly higher during hard condition compared to easy condition)	**Gaze distribution (% fixations)**<br>- Decreased fixations on external environment with task complexity (higher % on easy condition compared to hard condition)	NA
(Di Flumeri et al., 2018)	Mental workload	**Power values of sub-bands (frontal, AF3, AF4, F3, Fz, F4; and parietal sites, P3, P7, Pz, P4, P8, POz):**<br>- Ratio between theta power at frontal sites (ThetaF) and alpha power at parietal sites (AlphaP): ThetaF/AlphaP increases if mental workload increases<br>- ThetaF/AlphaP significantly increased with mental workload (hard segment)<br>- EEG-based index of mental workload (computed by as-SWLDA algorithm): selected features included Lower Theta over F4 and Upper Alpha over POz<br>- Higher sensitivity with respect to ET measures in discriminating impact of road complexity and traffic intensity on mental workload	**Gaze distribution (% fixations):**<br>- Fixations on external environment inversely correlated with mental workload (less fixations over the external environment with higher mental workload, hard segment – driver more focused on infrastructure and vehicles)	**NASA-TLX:**<br>- No significant differences in terms of subjective workload between normal and rush hour conditions
(Yang et al., 2020)	Mental workload	**Power values of sub-bands (prefrontal cortex: Fz, F3, F4, F7, F8, FP1, FP2)**	**Number of saccades:**<br>- Increases with the increase in place names on signs (more saccades needed	**Driving behavior (speed and acceleration):**<br>- Speed decreased significantly after seeing the signs

Study	Condition(s)	EEG Features	ET Features	Other Features
		- Mean absolute alpha power: inhibition of activity in prefrontal cortex with increased difficulty of task work (lower alpha band power of pre-frontal area = higher mental workload)<br>- Mean absolute alpha power decreases with the increase in place names on signs (mental workload increased)<br>- Only when number of place names was 7 or less, the effect of number of boards was significant (lower mean absolute alpha power for multi-board, higher mental workload)<br>- No difference between single and multi-board signs when number of place names was higher than 7	to locate target information, increased mental workload)<br>- Significantly higher in multi-board scenes than single-board scenes (regardless of number of place names)<br><br>**Fixation duration on the interest zone:**<br>- Increase with increase in place names on signs (more time spent on signs, extracting information)<br>- Multi-board required higher fixation duration when number of place names was 7 or less<br>- Single-board required higher fixation duration when number of places was higher than 7<br><br>**Fixations heatmaps:**<br>- Fixations points concentrated on left side of the board when one or two boards<br>- Fixation points concentrated on middle of the board when three boards	- Higher decrease of speed with increase in place names on signs<br>- Significant decrease of acceleration with increase in place names on signs<br>- Multi-board tends to produce decrease of acceleration (requires more time and higher mental workload to process information)<br>- Only when number of place names was 7 or less, the effect of number of boards was significant (lower acceleration for multi-board, higher mental workload)<br>- No difference in performance between single and multi-board signs when number of place names was higher than 7
(Angkan et al., 2024)	Cognitive load	**40 features from both time and frequency domains from each channel:**<br>- PSD (absolute, mean, maximum, minimum, median power)<br>- Spectral entropy<br>- Hjorth mobility and complexity<br>- Lempel-Ziv complexity<br>- Higuchi fractal dimension<br>- Raw signal (mean, minimum, maximum, mean, variance, and standard deviation)	**32 features related to:**<br>- pupil diameter (max, min, mean)<br>- blinks (count; max and mean duration)<br>- fixations (count; max, min, mean duration)<br>- saccades (count; max, min, mean duration; max, min, mean amplitude)<br>- gaze (max, min, mean peak velocity; max, min, mean peak acceleration; max, min, mean peak	**ECG: 53 features**<br>- Time-domain features: RMSSD, N-N intervals (mean, SD, SDSD, CV, CVSD, median, median absolute deviation, median CV, IQR, % of intervals differing more than 50 ms and 20ms, triangular interpolation), HRV triangular index<br>- Non-linear features (poincaré plot-based)

Study	Condition(s)	EEG Features	ET Features	Other Features
			deceleration; max, min, mean direction)	- Geometrical and other non-linear features<br>- Entropy features<br><br>**EDA: 30 features** (mean, median, standard deviation, skewness, kurtosis, entropy, interquartile range, AUC, squared AUC, median absolute deviation for raw data as well as phasic and tonic response)
(Eniyandunmo et al., 2024)	Mental workload	**Power values of sub-bands (AF4 and FC6 channels)**<br>Theta band in channels AF4 and FC6 selected:<br>- Previous study suggests that theta power increases during high cognitive task loads<br>- AF4 and FC6 channels had greatest difference in avg across easiest and hardest tasks	**Pupil diameter**<br>- Increase with increase in complexity (upward trend)	**ECG:** HR, NN, RMSSD-NN<br>**EDA:** SCL<br>- Increase with increase in complexity (upward trend)<br>**Facial EMG:** maximum amplitude<br>- Decrease with increase in complexity (downward trend)
(Shafiei et al., 2024)	Mental workload	**Features related to strength, search information, temporal network flexibility, integration and recruitment (frontal, parietal, occipital and temporal lobes)**<br>Key EEG features contributing to model performance per task:<br>- Average search information frontal lobe (matchboard task)<br>- Average recruitment occipital lobe (ring walk)<br>- Average recruitment temporal lobe (ring walk)<br>- Average strength temporal lobe (pattern cut, suturing)	**Features related to fixations (%), saccades (%), gaze direction (horizontal change rate, vertical change rate; dominant, non-dominant eye), pupil trajectory length (dominant, non-dominant eye), pupil diameter (avg and avg entropy; dominant, non-dominant eye)**<br>Key features contributing to model performance per task:<br>- Average pupil diameter dominant eye (matchboard and pattern cut tasks): pupillary response as measure of cognitive load (positive correlation)	**Performance:** Higher predicted workload correlated with decrease in performance across all tasks (increased workload adversely affects performance, negative correlation)

Study	Condition(s)	EEG Features	ET Features	Other Features
		- Average temporal network flexibility parietal lobe (pattern cut)<br>- Average integration between parietal lobe and other lobes (pattern cut)<br>- Average integration between occipital lobe and other lobes (suturing)	- Average pupil diameter non-dominant eye (matchboard, ring walk, suturing): pupillary response as measure of cognitive load (positive correlation)<br>- Pupil trajectory length non-dominant eye (matboard, ring walk, pattern cut)<br>- Saccades proportion (suturing)<br>- Horizontal gaze direction change dominant eye (suturing)	
(Barragan et al., 2022)	Cognitive workload	**Power values of sub-bands: delta, theta, alpha, gamma**<br>Differences due to cognitive load in frontal and temporal lobes; high cognitive load characterized by increased in theta and alpha activity:<br>- Increased theta activity in fronto-temporal channels (FP1, AF3, AF4, F7, F3, F4, F8, FC5, FC2, FC6, T7, C3, C4, T8, CP5) during high workload<br>- Increased alpha activity in fronto-temporal channels (F7, F8, FC5, FC6, T7, C4, T8, CP5, CP6) during high workload<br>- Suppressed delta activity in fronto-temporal channels (F7, FC5, T8) during high workload condition compared to low workload (participants could be highly dependent of visual feedback)<br>- Increased beta activity (F8, FC6, T8, Oz) and suppressed beta activity (Fz, F4, FC2, C3, Cz, CP5, CP2) during high workload	**Average pupil diameter, fixations (number and avg duration), scan path length, nearest neighbor index (nearest neighbor distance of fixations/avg distance of a randomly distributed set of fixations)**	NA

Study	Condition(s)	EEG Features	ET Features	Other Features
(Orlandi & Brooks, 2018)	Mental workload	**Power values of sub-bands: delta, theta, alpha, beta 1 (13-20), beta 2 (20-36))**<br>- Beta 1 and Beta 2 bands: higher response for difficult case (extended from the whole duration up to baseline-post)<br>- Beta 2: less score in homeport for easy case compared to difficult; higher score in foreign port than in homeport for easy case	**Pupil dilation**<br>- Significant effect on the factor phase: higher values during "approach" and "swing" phases than "closing" (higher level of response elicited by expectations related to the exercise just started)<br>- Significant differences between easy and difficult cases during "swing" phase<br>- Higher mean in "approach" phase for easy case<br>- Lower mean in "closing" phase for difficult case<br>- Highest scores in the difficult cases	**ECG: HR, HRV, IBI**<br>- Increment during difficult case<br>- Consistent HR pattern during the 5 phases (increasing value, with a decrement during last "baseline post" phase)<br>- Higher HR in foreign port compared to homeport for easy case<br>- Higher HR scores for difficult case in both ports<br>**NASA-TLX:**<br>- Mainly experienced mental workload and related to achievement of a desired result<br>- Less satisfaction in performance level with more difficult case (more effort, higher level of frustration)<br>**Likert-scale:**<br>- Consistent with NASA-TLX results: higher values for difficult cases than easy cases<br>- Slight increase of workload for foreign port compared to homeport<br>- Correlation with HR and pupil dilation (increased difficulty level induced increment of response level)
(Iqbal et al., 2024)	Mental workload	**Power values of sub-bands: delta (1–3 Hz), theta (4–7), alpha (8–13), beta 1 (14–20), beta 2 (21–30)**	**Pupil-based features**: PSD of pupil diameter signal in ranges (0–0.5 Hz, 0.5–1, 1–1.5 Hz, 1.5–2), Average pupil diameter<br>**Gaze-based features:** Longest Fixation duration/shortest Fixation	NA

Study	Condition(s)	EEG Features	ET Features	Other Features
			duration, Normalized Fixation Number (No. of fixations/ time), Normalized Fixation Duration (Total fixation duration/time), Standard deviation of fixation duration, Mean of fixation duration	
(Jimenez-Molina et al., 2018)	Mental workload	**Power and phase of the analytical signal obtained with the Transf. of Hilber: theta and alpha bands**<br>- Theta increases with mental workload (theta band related to mental activity)<br>- Alpha decreases with mental workload (alpha band related to relaxation)	**2 features from pupil dilation: mean and variance**<br>- Decrease in mental workload in the transition time windows between the analysis time window of one element and another<br>- Mean pupil diameter in transition windows smaller than in active windows (mental workload smaller when attention is switching than when it is focused)	**EDA: 6 features (**accumulated data, avg as a function of time and spectral power; phasic component: number of peaks, maximum modulus, and avg of the phasic component of the window)<br>**Body temperature: 2 features** (mean, median)<br>**ECG: 3 features** (mean, median, variance of mean absolute deviation)<br>**PPG: 3 features** (mean, standard deviation, RMS of HR)
(Lobo et al., 2016)	Mental workload	**Power values of sub-bands: alpha and theta powers (channels 1 to 8)**	**4 features: % eye closure (right and left), pupil diameter (right and left)**	**RT:**<br>- Low workload: minimum delay on execution of primary task, for transformation from passive to active<br>- Medium workload: medium delay effects on the execution of the primary task, for transformation from passive to active and for control phrases<br>- High workload: maximum delay effect on the execution of the primary task, for transformation of ambiguous phrases
(Mark et al., 2024)	Mental workload	**Power values of sub-bands: delta, theta, alpha, beta, gamma; power**	**Features related to pupil (diameter), saccades (velocity, length), fixations**	**fNIRS-based features:**

Study	Condition(s)	EEG Features	ET Features	Other Features
		**band ratios beta/(alpha + theta), theta/alpha, theta/beta, and (theta + alpha)/(beta + alpha)**<br>**Theta band:** broad significant responses during Working memory, Shifting attention and Situation awareness tasks:<br>**Alpha band:**<br>- Predominant response during Condition for working memory task (central parietal and right parietal occipital lobes) and Shifting attention and Situation awareness tasks (right central and central parietal regions)<br>- Broadly responsive to the main effect of Session and Condition during Vigilance task<br>- Strong response to Condition during Inhibitory control task (bilateral central alpha band from the frontal to occipital regions)<br>- Most sensitive to difficulty changes<br>**Delta band:** broadly responsive to the main effect of Condition during Vigilance task<br>**Theta/alpha ratio:**<br>- Widespread changes in theta/alpha ratio during Shifting attention task<br>- Localized changes (right hemisphere) during Situational awareness task<br>**Engagement ratio:** broad sensitivity to Session during Risk assessment and Inhibitory control tasks	**(number, avg duration, rate) and the fixation to saccade ratio:**<br>- Consistent response for saccade velocity as well as an increase in fixation count (known workload correlates)<br>- Pupil diameter provided the most information about workload over time (nearly every task showed significance for Session over time with a smaller diameter indicating increased skill)	- Strongest sensitivity to Session for all tasks with a preference for the bilateral PFC for Working Memory and Vigilance tasks, and specifically the right-lateral PFC for Shifting Attention and Inhibitory Control tasks<br>- Risk Assessment and Situation Awareness tasks instead engaged the left<br>**ECG-based features:**<br>- Strongest sensitivity to Session, few measures with sensitivity to Condition and interaction<br>- HRV measures and low frequency components: linear relationship with Session (decrease over time as expertise increased)<br>- HRV most consistent workload correlate<br>**PPG-based features:**<br>- Strongest sensitivity to Session, few measures with sensitivity to Condition and interaction<br>- Pulse width: highest sensitivity to Session (inverse linear relationship with time)<br>- Pulse peak: inverted-U shape curve in hard condition of Shifting attention task<br>**EOG-based features:**<br>- Mean saccade duration and amplitude increased as workload decreased over time (inverse linear relationship with

Study	Condition(s)	EEG Features	ET Features	Other Features
				workload); different timing of increasing in Inhibitory control task (larger increase early on in the hard condition and later for easy)<br>- Peak saccade velocity: inverse-U shape over the three sessions in several tasks (align with shape of moving from one learning plateau to the next)<br>**Behavioral measures:**<br>- Significant changes across all tasks, session and condition<br>- Task performance improved across sessions and decreased during harder conditions<br>- RT: highest sensitivity (increase across sessions and tasks)
(Aksu et al., 2024)	Mental workload	**Power values of sub-bands: theta, alpha, low beta, high beta, gamma; theta/alpha power ratio:**<br>**Variables associated with theta power (AF3, AF4, F7, F8, and FC5 channels):** strongest correlation with task difficulty level (as the task difficulty increased, theta power in the prefrontal, frontal, and frontal central regions also increased)<br>**Variables related to theta/alpha power ratios:** higher correlations in prefrontal, frontal and frontal central regions<br>**Alpha power (O1, O2, T7, P7 channels):** negative correlation with task difficulty level (as the task difficulty increased, the alpha power in	**Features related to:**<br>**- Fixations: number; durations (sum, mean, SD, maximum, minimum);**<br>**- Saccades: number; durations (sum, mean, SD, maximum, minimum); saccadic amplitude (mean, maximum, standard deviation)**<br>**- Pupil diameter: left eye and right eye (mean, maximum, and standard deviation); avg of both eyes**<br>**- Blinks: number; durations (sum, mean)**<br>All variables, except for fixation duration, had a positive correlation with mental workload	**NASA-TLX:**<br>- Significant correlation with 75 EEG variables, strongest with theta power (AF4 and F8) and theta/alpha (AF3, AF4, and F8): as the perceived mental workload intensified, there was an increase in prefrontal and frontal theta power. Conversely, as the perceived workload increases, there is a decrease in low beta power in the prefrontal, frontal, parietal, and occipital brain regions<br>- The number and total duration of blinks, the mean, maximum value, and standard deviation of saccade amplitude, and the number and

Study	Condition(s)	EEG Features	ET Features	Other Features
		the temporal, parietal, and occipital regions decreased)<br>**Alpha power (F7 and F8 channels):** positive correlation with task difficulty level (as the task difficulty increased, the alpha power in the frontal regions increased)<br>**Low beta power:** decrease across various brain regions as the task difficulty increases		total duration of saccades had the highest correlation with the NASA-TLX score<br>**Kruskal–Wallis test:** demonstrated significant differences in all eye tracking variables and in 49 EEG variables based on task difficulty levels<br>- The number and total duration of blinks, the number and total duration of saccades, and the mean of the left and right pupil diameter had the highest correlation with the task difficulty level<br>- 34 out of the 49 EEG variables exhibited a smooth increase or decrease in accordance with the change in task difficulty: in more challenging task conditions, the prefrontal, frontal, and central regions (AF3, AF4, F7, F8, FC5, and FC6) displayed higher theta power, while the temporal, parietal, and occipital regions (O1, O2, P7, P8, T7, and T8) exhibited lower alpha power. Additionally, beta power was lower in almost all brain regions
(Planke et al., 2021)	Mental workload	**Power values of sub-bands: theta, alpha and beta**<br>- EEG MWL model: effective in inferring MWL	**SPE, Blinks per minute, Pupil Diameter and Proportional Dwell Time**<br>**Pupil diameter:** positive correlation with task level (mental workload level)<br>**Blinks per minute:** negative correlation with mental workload level	**ECG:** HR<br>- Poor correlation with task level (not sensitive to changes in task load)<br>**CIs (from the mouse button clicks): number of mouse button clicks**

Study	Condition(s)	EEG Features	ET Features	Other Features
			**Proportional dwell time:** correlation with mental workload level<br>**SPE:** positive correlation with mental workload level	**-** Positive strong correlation with task level (as expected: task scenario highly dependent on more CIs as task levels increased)
(John et al., 2022)	Mental workload	**Frontal, parietal and occipital clusters of ICs; IC- ERSPs changes for each cluster; Power values of sub-bands for each IC:** EEG metrics sensitive to task load variations and task type<br><br>TRACKING TASK: increased frontal theta power and decreased occipital alpha power with increasing workload levels (increased working memory load experienced with increasing workload levels)<br>- Frontal cluster: significant increase in theta power from the low to the high level (and in between levels)<br>- Parietal cluster: no significant spectral power variations observed<br>- Occipital cluster: significant decrease in alpha power from the low to the high level (and in between levels)<br><br>COLLISION PREDICTION TASK: increased occipital delta power (increased allocation of attentional resources with increasing workload levels); increased frontal, parietal and occipital theta power (increase in memory load with increasing workload levels); decreased parietal alpha power	**ET metrics:** sensitive to task load variations, but not to task type<br>**Pupil size data:** pupil size increased significantly with the increasing workload for both tracking and collision prediction tasks (pupil dilates with increasing workload)<br>**Number of blinks:** number of blinks decreased significantly with increasing workload during tracking and collision prediction tasks (blink inhibition in higher workload conditions, blink rate inversely correlated with attentional levels and workload)	**Behavioral and performance measures:** significant drop in the performance with increasing workload levels in both the tracking and collision prediction tasks<br>- Tracking task: tracking accuracy decreased significantly with increasing levels of workload (workload manipulation in tracking prediction tasks successfully elicited significant performance variations)<br>- Collision prediction task: time before collision decreased with increasing workload; collision prediction miss proportion rate increased with increasing levels of workload (workload manipulation in collision prediction tasks successfully elicited significant performance variations)<br>**Heart Rate Variability:** sensitive to task load variations, but not to task type<br>**-** Significant change in the RMSSD for the different workload conditions (in both tracking and collision prediction tasks): negative correlation with mental workload in both tasks

Study	Condition(s)	EEG Features	ET Features	Other Features
		- Frontal cluster: significant increase in theta power from the low to the high level (and in between levels)<br>- Parietal cluster: significant increase in theta power and decrease in alpha power from the low to the high level (and in between levels)<br>- Occipital cluster: significant increase in the delta and theta power from the low to the high level (and in between levels)		

TABLE 5.3. CLASSIFIERS AND PERFORMANCE

Study	Labels	Classifier(s)	Performance metrics	Cross-validation	Results	Modalities comparisons
(Borys, Plechawska-Wójcik, et al., 2017)	Dataset 1: Binary classification (cognitive workload or no-task condition)<br><br>Dataset 2: multiclass (high cognitive workload, low cognitive workload, no-task)	Decision trees; discriminant analysis (linear and quadratic) LR; SVMs; kNN; ensemble classifiers	Accuracy, AUC, PPV	5-k	**Dataset 1**<br>- SVM + 6 ET features: best accuracy (90%)<br>- kNN + 12 ET features: similar accuracy (90%); higher positive prediction rate for cognitive workload than no-task<br>**Dataset 2**<br>- kNN + 17 ET features: maximum accuracy (73%); high positive prediction rate for no-task, poor distinction between workload conditions<br>- Bagged Trees + 13 features: better for classifying all states	None of the EEG features included as input in classification models with best accuracy<br>**Dataset 1**<br>- EEG+ET (Bagged trees): 85.2% accuracy<br>- Best 3 EEG features (Quadratic Discriminant with diagonal covariance regularization): 73.9% accuracy<br>- Best 12 ET features (kNN, SVMs): 90.4%<br>**Dataset 2**<br>- EEG+ET (Bagged trees): 63.5% accuracy

Study	Labels	Classifier(s)	Performance metrics	Cross-validation	Results	Modalities comparisons
						- Best 5 EEG features (kNN): 50.4% accuracy<br>- Best 17 ET features (kNN): 73%
(Kujur et al., 2022)	Cognitive load level (easy, hard)	XGBoost, AutoML	Precision, recall, f1, ROC_AUC, accuracy	XGBoost: 5-k (80% train, 20% test)<br>AutoML: single train-test split (75% train, 25% test)	- XGBoost (fold 1, 56 features): best result (accuracy 66.3%)<br>- AutoML (56 features): similar result (65%)<br>- XGBoost (top 10 features): more accurate than AutoML	EEG+ET: improved mental workload estimation (EEG+ET combination more informative in distinguishing levels of mental workload)
(Guo et al., 2024)	Mental workload level: low (observation/ report), strike (medium), stage (high);<br><br>**2 labelling methods:** NASA-TLX scale and performance-based (operation time selected as performance indicator; short operation time = high performance)	LDA	Accuracy	NA	**NASA-TLX labeling:**<br>- Accuracy: 68.57%<br>- Fluctuations in search stage workload may cause labeling noise<br>**Performance-based labeling:**<br>- Accuracy: 84.29% (improvement of 15.72% over NASA-TLX labelling)<br>- Captures fluctuations, improving classification accuracy	NA
(Singh et al., 2021)	Multimodal (high mental workload, low mental workload, resting)	kNN, DT, AB, GNB, LDA, QDA, SVM	Accuracy	**Traditional design**<br><u>Intra-subject</u><br>- 5-k for grid search with 70% data<br>- 10-k final model	<u>Intra-subject</u><br>- Traditional validation design: 74.8 % (highest accuracy)<br>- Ecological validation design: 59.6% (significant drop in accuracy)	**Combinations: ET, EEG, ECG, EEG+ET, EEG+ECG, ECG+ET, EEG+ECG+ET**<br><u>Intra-subject</u><br>- ECG-only features gave significantly

Study	Labels	Classifier(s)	Performance metrics	Cross-validation	Results	Modalities comparisons
				Inter-subject<br>- Leave-2-out<br>- 5-k parameter tuning<br>**Ecological design**<br>Intra-subject<br>- 5-k for parameter tuning (2 groups)<br>Inter-subject<br>- Leave-2-out<br>- 5-k for parameter tuning	- Best performance reached with traditional validation design using the AB, LDA, and SVM ones; LDA and SVM maximum performance<br>- No difference between classifiers was significant with the ecological validation design<br>Inter-subject:<br>- Ecological validation design: 59.8% (highest accuracy)<br>- Traditional design: 57.4%<br>- No significant impact of validation method nor the features used. Only a main effect of the classifier used<br>- LDA achieved significantly higher accuracy than DT	higher estimation accuracy (75%) and ET-only the worst (independent of classifier and validation design)<br>- EEG+ECG also reached high classification accuracy (traditional design; AB, LDA, and SVM)<br>- EEG+ECG+ET also good results (traditional design; LDA)<br>Inter-subject:<br>- ECG-only best performance (traditional design; ecological design)
(Angkan et al., 2024)	Cognitive load (based on PAAS subjective ratings):<br>**Binary classification:** "low" (ratings 1–4) and "high" (ratings 5–9)<br>**Ternary classification**: "low" (1–3), "medium" (4–6) and "high" (7–9)	AB, DT, NB, kNN, LDA. RF, SVM, XGBoost, MLP, 2 CNNs (VGG-style and ResNet style)	Accuracy, F1 scores	10-k and LOSO	**10-k binary:**<br>- Best accuracy: 83.67% (XGBoost classifier with all 4 modalities)<br>- Second-best: 83.02% (XGBoost with EEG+ECG+ET)<br>- XGBoost was the best-performing model, followed by RF<br>**LOSO binary:**<br>- Best accuracy: 76.17% (VGG-style with EEG+ECG+EDA)<br>- Second-best: 76.04% (EEG+ECG+ET)<br>**10-k ternary:**	Using all 4 modalities (EEG+ECG+EDA+ET) outperforms the rest:<br>**10-k binary:**<br>ALL > EEG+ECG+ET > EEG+EDA+ET > EEG+ECG+EDA > EEG+ECG > EEG +ET > EEG+EDA > EEG<br>**LOSO binary:** ALL > EEG+EDA+ET > EEG+ECG+EDA > EEG+ECG+ET > EEG+ET > EEG+EDA > EEG+ECG > EEG<br>**10-k ternary:** ALL > EEG+EDA+ET >

Study	Labels	Classifier(s)	Performance metrics	Cross-validation	Results	Modalities comparisons
					- Best accuracy: 74.08% (XGBoost with all 4 modalities)<br>- Second-best: 73.60% (XGBoost with EEG+EDA+ET)<br>- Among deep learning models, VGG-style networks achieved the best accuracy (67.12%) using EEG+EDA+ET<br>**LOSO ternary:**<br>- Best accuracy: 64.53% (ResNet-style with EEG+ECG+ET)<br>- Second-best: 63.56% (VGG-style using all 4 modalities)	EEG+ECG+ET > EEG+ECG+EDA > EEG+ET > EEG+ECG > EEG+EDA > EEG<br>**LOSO ternary:** ALL > EEG+ECG+ET > EEG+EDA+ET > EEG+ET > EEG+ECG+EDA > EEG+ECG > EEG+EDA > EEG
(Eniyandunmo et al., 2024)	Mental workload levels (based on subjective ratings):<br>- Rest: Normalized range (0, 0.1)<br>- Low: (0.1, 0.4)<br>- Adequate: (0.4, 0.7)<br>- High: (0.7, 1)	Function-on-Function Linear Regression Model	Accuracy (mean, SD), F1 score (mean, SD)	5-k	**Raw Physiological Data: model:** outperformed model relying on extracted features<br>- ECG+EDA+EEG: accuracy 90%, SD 0.07 (better performance)<br>- All physiological signals combined: accuracy 90%<br>- 6 additional signal combinations demonstrated accuracy 90% and 0.08 SD<br>- ECG+ET+EEG: accuracy 88%, lowest SD 0.05<br>- EDA-only: accuracy 87%, SD 0.06<br>**Extracted features model:**	**Raw physiological data:**<br>- 90% accuracy and 0.07-0.08 SD in 9 cases (all 5 signals; ECG+EDA+EMG+ET; ECG+EDA+ET+EEG; ECG+EMG+ET+EEG; ECG+EDA+ET; ECG+EDA+EEG; ECG+ET+EEG; EDA+EMG+ET; EDA+ET+EEG)<br>- Optimal combination: ECG+EEG+(EDA or ET)<br>**Extracted features:**

Study	Labels	Classifier(s)	Performance metrics	Cross-validation	Results	Modalities comparisons
					- HR + SDNN + SCL + facial EMG: accuracy 73%, SD 0.01 (highest performance)<br>- SCL-only: accuracy 73%, SD 0.05	- 73% accuracy in 9 cases (SCL, SCL+NN; SCL+SDNN; SCL + mean pupil diameter; SCL + facial EMG; HR+SDNN+SCL; NN+SCL + mean pupil diameter; SCL + facial EMG + mean pupil diameter; HR+SDNN+SCL+ facial EMG)<br>- HR+SDNN+SCL+ facial EMG: lowest SD (0.01)<br>- SCL feature contained in all best performing combinations (skin conductance greatly improves performance of mental workload models)
(Shafiei et al., 2024)	Cognitive workload score (based on Surg-TLX questionnaire)	XGBoost	MAE, RMSE, R2	5-k	Reasonably low MAE and RMSE values, and high R2 values	EEG+ET integration significantly increased performance for most surgical tasks:<br>- Matchboard, Ring Walk and Suturing tasks: EEG+ET model performs better (task requires cognitive and visuospatial skills, both playing a significant role)<br>- Pattern Cut task: combined model does

Study	Labels	Classifier(s)	Performance metrics	Cross-validation	Results	Modalities comparisons
						not significantly outperform single models (one of the data types might already provide sufficient information for mental workload assessment in this task)<br><br>R2 and MAE values across four tasks:<br>**- EEG + ET**: 0.81 – 0.83, 4.5-11.11<br>- EEG: 0.75 – 0.86, 6.55-14.06<br>- ET: 0.64-0.75, 6.54-13.54
(Barragan et al., 2022)	Low cognitive load and high cognitive load	**Dataset 1 (EEG-only):** recurrent LSTM, convolutional LSTM, feedforward NN<br>**Dataset 2 (EEG+ET):** NN, kNN, RF, SVM	Accuracy	Grid-search	**Dataset 1**<br>- Best accuracy: recurrent LSTM (78% at 170s sequence length)<br>- Positive correlation between sequence length and accuracy (increasing from 10 to 100s improved from 68% to 78%)<br>**Dataset 2**<br>- Best accuracy: NN (80.25% at 125s)<br>- Accuracy not as correlated with sequence length (increasing from 25 to 75s improved 2%)	- EEG+ET improves robustness of prediction<br>- Including ET allows for 25s reduction of input data without compromising accuracy
(Iqbal et al., 2024)	Mental workload levels (low, medium, high),	DT-based model	Accuracy, confusion matrix	10-k	- Low workload: best accuracy with pupil-based features (64.4%)	Fusion led to increase of accuracy of up to 22% (different features

Study	Labels	Classifier(s)	Performance metrics	Cross-validation	Results	Modalities comparisons
	based on IAA and performance				- Medium workload: pupil and EEG-based better (54.0%) than gaze-based (53.0%)<br>- High workload: best accuracy with gaze-based (52.6%)<br>- Binary classification (low and high workload) increased accuracy (82.15%) and correctly classifies 70.5% of low workload and 87.7% of high workload	capture different aspects of cognitive workload):<br>- Model using pupil, gaze, and EEG features: 66.8% accuracy
(Jimenez-Molina et al., 2018)	Mental workload levels based on pupil diameter (4 classes)	Multinomial LR, multi-class SVM, MLP	Accuracy, recall, precision, kappa statistic	10-k	**Multinomial LR**<br>- Worst performer: 51.42% accuracy, Kappa value of 5.92%<br>- Fails to effectively classify multiple classes<br>**Multi-class SVM**<br>- Moderate performance: 66.48% accuracy (all features) and 70.03% accuracy (RF-RFE-selected features)<br>- Class imbalance has a minor impact<br>**MLP**<br>- Best performer: 93.7% accuracy (all features)<br>- Recall: 95.28%, Precision: 92.06%, Kappa: 91.24%	Comparison of sensors with MLP as a supervised learning model:<br>- EEG best standalone sensor: accuracy of 70.91% (possibly due to high feature richness)<br>- The other sensors separately have a very low level of accuracy<br>- Optimal Sensor Combination: EEG+EDA+PPG(HR), 86.27% accuracy<br>- Discarded Sensors: Temperature and ECG (poor performance)
(Lobo et al., 2016)	Mental workload level based on RT (low, medium, high workload)	RF and kNN	Macro avgd precision, recall and F-score	Stratified	Best results with kNN when k=3. Different approaches tested:	NA

Study	Labels	Classifier(s)	Performance metrics	Cross-validation	Results	Modalities comparisons
					- Individual classification performance: F-score ratios 0.99 (overfitting)<br>- Single models applied to the rest of users: best F-score 0.312 (not enough)<br>- Each user used once as a test set while the remaining sampled formed the training set: global avg F-score 0.332 (better than 2nd but still unsatisfactory)	
(Aksu et al., 2024)	Mental workload levels (different models tested: 4 class, 3 class, 2 class)	kNN, RF, NN, SVM, GBM, XGBoost, Light GBM	Accuracy, Kappa, MCC, AUC	5-k (for dimension reduction using Light GBM)	- Light GBM: highest performance (71.96% accuracy)<br>- Also good results for XGBoost (71.96%), SVM (62.15%) and GBM (61.93%)<br>- Models made the most misclassifications for 1-back task, and between classes 0-1 and 2-3<br><br>**Additional steps with Light GBM model:**<br>(1) Class-reduction (early stopping): reducing the number of classes led to better classification results<br>- Three-class mode (0-back, 2-back, and 3-back tasks low–medium–high mental workload): 80.49% accuracy<br>- Two-class model (0-back and 3-back tasks): 89.63% accuracy	Models using Light GBM:<br>- EEG+ET: better results than single modality<br>- ET-only model better performance (65.67% accuracy) than EEG-only (56.15% accuracy)<br>- Key variables: right and left pupil diameter; variables associated with blink, saccade, and saccadic amplitude; prefrontal and frontal theta and theta/alpha; occipital alpha; and temporal, parietal, and occipital gamma power

Study	Labels	Classifier(s)	Performance metrics	Cross-validation	Results	Modalities comparisons
					(2) Dimension reduction: 34 features for the four-class model (20 EEG, 14 ET); 91 features for the 3-class model; 110 for the 2-class model<br>(3) After dimension reduction, hyper-parameter adjustment, and class-reduction: 76.59% avg accuracy performance in the 34-variable model (4-class); maximum accuracy of 81.11%. at fold 4	
(Planke et al., 2021)	Mental workload levels (4 levels: very low, low, medium and high)	ANFIS (with 4 fuzzy sets)	MAE	**Session 1:**<br>5-k, 50% hold-out validation<br>**Session 2:**<br>online (real-time testing)	**Session 1** (calibrating and validating the ANFIS model in offline processing)<br>- Average MAE: 0.47 ± 0.09 (first half), 0.81 ± 0.38 (second half, unseen data), 0.60 ± 0.10 (all data)<br>**Session 2** (online validation):<br>- Cross-session of models 1-5: MAE of around 0.7<br>- Models 6-7: 0.61-0.69	**Session 1:**<br>- Best performing models: those containing 2 or more features that strongly correlated with task level (EEG, SPE, CI)<br>- Selected ANFIS models as optimal candidates: low error rate (0.28-0.51)<br>- Best result: subject-specific feature combination with CI (0.28 ± 0.05)<br>- All 7 features: 0.36±0.1 (comparable result)<br>**Session 2:**<br>- Cross-session of models 1-5 (not including EEG): best model SPE+CI

Study	Labels	Classifier(s)	Performance metrics	Cross-validation	Results	Modalities comparisons
(John et al., 2022)	**Case 1:** tracking task performance<br>**Case 2:** collision task performance	Multiple linear regression	Variance	NA	**Case 1:**<br>- Predictors: EEG metric (frontal theta and occipital alpha) + ET metric (pupil size and blink rate) + HRV<br>- Model explained 54.3% of variance, significant predictor of tracking performance<br>**Case 2:**<br>- Predictors: EEG metric (frontal theta, parietal theta and alpha, occipital delta and theta) + ET metric (pupil size and blink rate) + HRV<br>- Model explained 61.7% of variance, significant predictor of collision performance	**Case 1:**<br>- EEG and ET contributed significantly to the model, HRV did not<br>- ET higher contributing predictor than EEG metric (significant visual load in tracking task)<br>**Case 2:**<br>- EEG and ET contributed significantly to the model, HRV did not<br>- EEG higher contributor predictor than ET (significant internal concentration to anticipate collision)

ACRONYMS

Acronym	Abbreviations
**1D-CNN**	One-dimensional Convolutional Neural Network
**2D-CNN**	Two-dimensional Convolutional Neural Networks
**AAT**	Alpha Attenuation Test
**AB**	AdaBoost
**ANFIS**	Adaptive Neuro Fuzzy Inference System
**ANN**	Artificial Neural Network
**AOIs**	Areas of Interest
**A-P300**	Auditory P300 test
**ART**	Auditory Response Test
**as-SWLDA**	Automatic stop-StepWise Linear Discriminant Analysis
**ATCo(s)**	Air Traffic Controller(s)
**AUC**	Area Under the Curve
**avg**	Average
**BOLD**	Blood-Oxygen-Level-Dependent
**CART**	Classification And Regression Tree
**CC**	Correlation Coefficient
**CI**	Control Input
**CNN**	Convolutional Neural Network
**CV**	Coefficient of Variation
**CVSD**	Coefficient of Variation of Successive Differences
**CVT**	Continuous Visuomotor Tracking
**DE**	Differential Entropy
**DSSQ**	Dundee Stress State Questionnaire
**DT**	Decision Tree
**ECG**	Electrocardiogram
**EDA**	Electrodermal Activity
**ED**	External Distraction
**EEG**	Electroencephalogram
**EMG**	Electromyogram
**EOG**	Electrooculogram
**ERD**	Event-Related Desynchronization
**ERSP**	Event-Related Spectral Power
**ET**	Eye-tracking
**FAA**	Frontal Alpha Asymmetry
**fNIRS**	Functional Near-Infrared Spectroscopy
**GBM**	Gradient Boosting Machine
**GELM**	Discriminative Graph Regularized Extreme Learning Machine
**GLMM**	Generalized Linear Mixed Model
**GNB**	Gaussian Naive Bayes
**hEOG**	Horizontal Electrooculogram
**HF**	High Frequency
**HMI**	Human-Machine Interface
**HMM**	Hidden Markov Model

**HR**	Heart Rate
**HRV**	Heart Rate Variability
**IAA**	Integral Absolute Abnormality
**IAF**	Individual Alpha Frequency
**IBI**	Inter-beat Interval
**IC**	Independent Component
**ICA**	Index of Cognitive Activity
**ID**	Internal Distraction
**IQR**	Interquartile Range
**ISA**	Instantaneous Self-Assessment questionnaire
**KDT**	Karolinska Drowsiness Test
**kNN**	k-Nearest Neighbors
**KSS**	Karolinska Sleepiness Scale
**KSS-CN**	Chinese Karolinska Sleepiness Scale
**LC**	Letter Counting
**LDA**	Linear Discriminant Analysis
**LF**	Low Frequency
**LOSO**	Leave One Subject Out
**LPSD**	Logarithm Power Spectrum Density
**LR**	Logistic Regression
**LSTM**	Long Short-Term Memory network
**MAE**	Mean Absolute Error
**MATB**	Multi-Attribute Task Battery
**MCC**	Matthews Correlation Coefficient
**MCT**	Mackworth Clock Test
**MLP**	Multi-Layer Perceptron
**MUM-T**	Manned-Unmanned Teaming
**MWS**	Mind-Wandering Scale
**n**	Number
**NA**	Not Available
**NASA-TLX**	NASA Task Load Index
**NB**	Naive Bayes
**NN**	N-N interval
**PERCLOS**	Percentage of Eyes Closed
**PFC**	Pre-frontal cortex
**PLI**	Phase Lag Index
**POMS**	Profile of Mood States
**PPG**	Photoplethysmography
**PPV**	Prediction Precision Value
**PSD**	Power Spectral Density
**PSV**	Peak Saccadic Velocity
**PVT**	Psychomotor Vigilance Task
**QDA**	Quadratic Discriminant Analysis
**R2**	R-squared
**ResNet**	Residual Neural Network
**RF**	Random Forest

**RF-RFE**	Random Forest and Recursive Feature Elimination
**RMS**	Root Mean Square
**RMSE**	Root Mean Square Error
**RMSSD**	Root Mean Square of Successive Differences
**RMSSD-NN**	Root Mean Square of Successive Differences of N-N interval
**ROC**	Receiver-Operating Characteristic
**RR**	R-R interval
**RSME**	Rating Scale Mental Effort
**RT**	Reaction Time
**SCL**	Skin Conductance Level
**SCWT**	Stroop Color Word Task
**SDNN**	Standard Deviation of N-N interval
**SDSD**	Standard Deviation of Successive Differences
**SPE**	Scan Pattern Entropy
**SRT**	Stroop Task
**SSS**	Stanford Sleepiness Scale
**Surg-TLX**	Surgery Task Load Index
**SVM**	Support Vector Machine
**TDS**	Transcranial Doppler Sonography
**TTFF**	Time-to-First-Fixation
**UAV**	Unmanned Aerial Vehicle
**USAF**	U.S. Air Force
**VAS**	Visual Analogue Scale
**vEOG**	Vertical Electrooculogram
**VGG**	Visual Geometry Group
**V-P300**	Visual P300 test
**VR**	Virtual Reality
**VRT**	Visual Response Test
**VUR**	Voiced-Unvoiced Ratio
**W-DCGAN**	Wasserstein Deep Convolutional Generative Adversarial Network
**WL**	Workload
**XGBoost**	eXtreme Gradient Boosting

REFERENCES

Aksu, Ş. H., Çakıt, E., & Dağdeviren, M. (2024). Mental Workload Assessment Using Machine Learning Techniques Based on EEG and Eye Tracking Data. *Applied Sciences*, *14*(6), Article 6. https://doi.org/10.3390/app14062282

Angkan, P., Behinaein, B., Mahmud, Z., Bhatti, A., Rodenburg, D., Hungler, P., & Etemad, A. (2024). Multimodal Brain–Computer Interface for In-Vehicle Driver Cognitive Load Measurement: Dataset and Baselines. *IEEE Transactions on Intelligent Transportation Systems*, *25*(6), 5949-5964. IEEE Transactions on Intelligent Transportation Systems. https://doi.org/10.1109/TITS.2023.3345846

Arsen'ev, G. N., Tkachenko, O. N., Ukraintseva, Yu. V., & Dorokhov, V. B. (2015). Prediction of the Moments at which Critical Decreases in Levels of Arousal Occur Using Visuomotor Coordination Parameters. *Neuroscience and Behavioral Physiology*, *45*(6), 715-723. https://doi.org/10.1007/s11055-015-0134-4

Asish, S. M., Kulshreshth, A. K., Borst, C. W., & Sutradhar, S. (2024). Classification of Internal and External Distractions in an Educational VR Environment Using Multimodal Features. *IEEE Transactions on Visualization and Computer Graphics*, *30*(11), 7332-7342. IEEE Transactions on Visualization and Computer Graphics. https://doi.org/10.1109/TVCG.2024.3456207

Barragan, J. A., Yang, J., Yu, D., & Wachs, J. P. (2022). A neurotechnological aid for semi-autonomous suction in robotic-assisted surgery. *Scientific Reports*, *12*, 4504. https://doi.org/10.1038/s41598-022-08063-w

Bodala, I. P., Li, J., Thakor, N. V., & Al-Nashash, H. (2016). EEG and Eye Tracking Demonstrate Vigilance Enhancement with Challenge Integration. *Frontiers in Human Neuroscience*, *10*. https://doi.org/10.3389/fnhum.2016.00273

Borys, M., Plechawska-Wójcik, M., Wawrzyk, M., & Wesołowska, K. (2017). Classifying Cognitive Workload Using Eye Activity and EEG Features in Arithmetic Tasks. En R. Damaševičius & V. Mikašytė (Eds.), *Information and Software Technologies* (pp. 90-105). Springer International Publishing. https://doi.org/10.1007/978-3-319-67642-5_8

Borys, M., Tokovarov, M., Wawrzyk, M., Wesołowska, K., Plechawska-Wójcik, M., Dmytruk, R., & Kaczorowska, M. (2017). An analysis of eye-tracking and electroencephalography data for cognitive load measurement during arithmetic tasks. *2017 10th International Symposium on Advanced Topics in Electrical Engineering (ATEE)*, 287-292. https://doi.org/10.1109/ATEE.2017.7905130

Chua, E. C.-P., Tan, W.-Q., Yeo, S.-C., Lau, P., Lee, I., Mien, I. H., Puvanendran, K., & Gooley, J. J. (2012). Heart Rate Variability Can Be Used to Estimate Sleepiness-related Decrements in Psychomotor Vigilance during Total Sleep Deprivation. *Sleep*, *35*(3), 325. https://doi.org/10.5665/sleep.1688

Di Flumeri, G., Borghini, G., Aricò, P., Sciaraffa, N., Lanzi, P., Pozzi, S., Vignali, V., Lantieri, C., Bichicchi, A., Simone, A., & Babiloni, F. (2018). EEG-Based Mental Workload Neurometric to Evaluate the Impact of Different Traffic and Road Conditions in Real Driving Settings. *Frontiers in Human Neuroscience*, *12*, 509. https://doi.org/10.3389/fnhum.2018.00509

Di Flumeri, G., Borghini, G., Aricò, P., Sciaraffa, N., Lanzi, P., Pozzi, S., Vignali, V., Lantieri, C., Bichicchi, A., Simone, A., & Babiloni, F. (2019). EEG-Based Mental Workload Assessment During Real Driving: A Taxonomic Tool for Neuroergonomics in Highly Automated Environments. En H. Ayaz & F. Dehais (Eds.), *Neuroergonomics* (pp. 121-126). Academic Press. https://doi.org/10.1016/B978-0-12-811926-6.00020-8

Di Flumeri, G., De Crescenzio, F., Berberian, B., Ohneiser, O., Kramer, J., Aricò, P., Borghini, G., Babiloni, F., Bagassi, S., & Piastra, S. (2019). Brain–Computer Interface-Based Adaptive Automation to Prevent Out-Of-The-Loop Phenomenon in Air Traffic Controllers Dealing With Highly Automated Systems. *Frontiers in Human Neuroscience*, *13*. https://doi.org/10.3389/fnhum.2019.00296

Diaz-Piedra, C., Rieiro, H., Cherino, A., Fuentes, L. J., Catena, A., & Di Stasi, L. L. (2019). The effects of flight complexity on gaze entropy: An experimental study with fighter pilots. *Applied Ergonomics*, *77*, 92-99. https://doi.org/10.1016/j.apergo.2019.01.012

Eniyandunmo, D., Shin, M., Lee, C., Anwar, A., Kim, E., Kim, K., Kim, Y. H., & Lee, C. (2024). Utilising raw psycho-physiological data and functional data analysis for estimating mental workload in human drivers. *Ergonomics*, *0*(0), 1-17. https://doi.org/10.1080/00140139.2024.2379949

Esposito, A., Braccili, E., Sgrò, F., Chiarantano, E., D'Ippolito, M., Pisotta, I., Bigioni, A., Guerrieri, A., Mattia, D., & Cincotti, F. (2022). Attention, Boredom and Mind Wandering during a Vigilance Task: EEG and

Ocular Markers. *2022 IEEE International Conference on Metrology for Extended Reality, Artificial Intelligence and Neural Engineering (MetroXRAINE)*, 477-482. https://doi.org/10.1109/MetroXRAINE54828.2022.9967678

Farha, N. A., Al-Shargie, F., Tariq, U., & Al-Nashash, H. (2022). Brain Region-Based Vigilance Assessment Using Electroencephalography and Eye Tracking Data Fusion. *IEEE Access*, *10*, 112199-112210. IEEE Access. https://doi.org/10.1109/ACCESS.2022.3216407

Gündoğdu, S., Çolak, Ö. H., Doğan, E. A., Gülbetekin, E., & Polat, Ö. (2021). Assessment of mental fatigue and stress on electronic sport players with data fusion. *Medical & Biological Engineering & Computing*, *59*(9), 1691-1707. https://doi.org/10.1007/s11517-021-02389-9

Gündoğdu, S., Doğan, E. A., Gülbetekin, E., Halil, Ç. Ö., & Polat, Ö. (2019). Evaluation of the EEG Signals and Eye Tracker Data for Working Different N-Back Modes. *Traitement Du Signal (TS)*, *36*(6), 493-500. https://doi.org/10.18280/ts.360603

Guo, M., Duan, P., Jin, X., Huang, Q., & Wei, Y. (2024). A Performance-based Mental Workload Identification Method for Special Vehicle Crews. *Physiology & Behavior*, 114706. https://doi.org/10.1016/j.physbeh.2024.114706

He, Q., Li, W., Fan, X., & Fei, Z. (2016). Evaluation of driver fatigue with multi-indicators based on artificial neural network. *IET Intelligent Transport Systems*, *10*(8), 555-561. https://doi.org/10.1049/iet-its.2015.0021

Hopstaken, J. F., van der Linden, D., Bakker, A. B., Kompier, M. A. J., & Leung, Y. K. (2016). Shifts in attention during mental fatigue: Evidence from subjective, behavioral, physiological, and eye-tracking data. *Journal of Experimental Psychology: Human Perception and Performance*, *42*(6), 878-889. https://doi.org/10.1037/xhp0000189

Huo, X.-Q., Zheng, W.-L., & Lu, B.-L. (2016). Driving fatigue detection with fusion of EEG and forehead EOG. *2016 International Joint Conference on Neural Networks (IJCNN)*, 897-904. https://doi.org/10.1109/IJCNN.2016.7727294

Iqbal, M. U., Srinivasan, B., & Srinivasan, R. (2024). Multi-class classification of control room operators' cognitive workload using the fusion of eye-tracking and electroencephalography. *Computers & Chemical Engineering*, *181*, 108526. https://doi.org/10.1016/j.compchemeng.2023.108526

Jimenez-Molina, A., Retamal, C., & Lira, H. (2018). Using Psychophysiological Sensors to Assess Mental Workload During Web Browsing. *Sensors*, *18*(2), Article 2. https://doi.org/10.3390/s18020458

John, A. R., Singh, A. K., Do, T.-T. N., Eidels, A., Nalivaiko, E., Gavgani, A. M., Brown, S., Bennett, M., Lal, S., Simpson, A. M., Gustin, S. M., Double, K., Walker, F. R., Kleitman, S., Morley, J., & Lin, C.-T. (2022). Unraveling the Physiological Correlates of Mental Workload Variations in Tracking and Collision Prediction Tasks. *IEEE Transactions on Neural Systems and Rehabilitation Engineering*, *30*, 770-781. IEEE Transactions on Neural Systems and Rehabilitation Engineering. https://doi.org/10.1109/TNSRE.2022.3157446

Kujur, A., Bhattacharya, A., Sharma, G., & Kumar, J. (2022). Prediction of Workload under Distraction using Supervised Learning Algorithms. *2022 3rd International Conference on Issues and Challenges in Intelligent Computing Techniques (ICICT)*, 1-5. https://doi.org/10.1109/ICICT55121.2022.10064593

Larue, G. S., Rakotonirainy, A., & Pettitt, A. N. (2015). Predicting Reduced Driver Alertness on Monotonous Highways. *IEEE Pervasive Computing*, *14*(2), 78-85. IEEE Pervasive Computing. https://doi.org/10.1109/MPRV.2015.38

Li, B., Wang, X., Wu, Y., & Zhu, X. (2023). Research on Driver KSS Rating Prediction Model Based on EU 2021/1341 DDAW. *2023 International Conference on Artificial Intelligence and Automation Control (AIAC)*, 195-201. https://doi.org/10.1109/AIAC61660.2023.00056

Lobo, J. L., Ser, J. D., De Simone, F., Presta, R., Collina, S., & Moravek, Z. (2016). Cognitive workload classification using eye-tracking and EEG data. *Proceedings of the International Conference on Human-Computer Interaction in Aerospace*, 1-8. https://doi.org/10.1145/2950112.2964585

Mark, J. A., Curtin, A., Kraft, A. E., Ziegler, M. D., & Ayaz, H. (2024). Mental workload assessment by monitoring brain, heart, and eye with six biomedical modalities during six cognitive tasks. *Frontiers in Neuroergonomics*, *5*, 1345507. https://doi.org/10.3389/fnrgo.2024.1345507

Matthews, G., Reinerman-Jones, L. E., Barber, D. J., & Abich, J. (2015). The Psychometrics of Mental Workload: Multiple Measures Are Sensitive but Divergent. *Human Factors*, *57*(1), 125-143. https://doi.org/10.1177/0018720814539505

Orlandi, L., & Brooks, B. (2018). Measuring mental workload and physiological reactions in marine pilots: Building bridges towards redlines of performance. *Applied Ergonomics*, *69*, 74-92. https://doi.org/10.1016/j.apergo.2018.01.005

Pan, Y., Guo, Z., Zhao, Y., Zhou, M., Yang, L., Zhang, J., & Li, G. (2024). A 2-D Vigilance Estimation Method for High-Speed Rail Drivers With Multimodal Sensors. *IEEE Sensors Journal*, *24*(18), 28982-28994. IEEE Sensors Journal. https://doi.org/10.1109/JSEN.2024.3433566

Planke, L. J., Gardi, A., Sabatini, R., Kistan, T., & Ezer, N. (2021). Online Multimodal Inference of Mental Workload for Cognitive Human Machine Systems. *Computers*, *10*(6), Article 6. https://doi.org/10.3390/computers10060081

Poudel, G. R., Innes, C. R., Bones, P. J., Watts, R., & Jones, R. D. (2012). Losing the struggle to stay awake: Divergent thalamic and cortical activity during microsleeps. *Human Brain Mapping*, *35*(1), 257. https://doi.org/10.1002/hbm.22178

Poudel, G. R., Innes, C. R. H., Bones, P. J., & Jones, R. D. (2010). The relationship between behavioural microsleeps, visuomotor performance and EEG theta. *2010 Annual International Conference of the IEEE Engineering in Medicine and Biology*, 4452-4455. https://doi.org/10.1109/IEMBS.2010.5625956

Previc, F. H., Lopez, N., Ercoline, W. R., Daluz, C. M., Workman, A. J., Evans, R. H., & Dillon, N. A. (2009). The effects of sleep deprivation on flight performance, instrument scanning, and physiological arousal in pilots. *International Journal of Aviation Psychology*, *19*(4), 326-346. https://doi.org/10.1080/10508410903187562

Reßing, C., Oschinsky, F. M., Klesel, M., Niehaves, B., Riedl, R., Suwandjieff, P., Wriessnegger, S. C., & Müller-Putz, G. R. (2022). Investigating Mind-Wandering Episodes While Using Digital Technologies: An Experimental Approach Based on Mixed-Methods. En F. D. Davis, R. Riedl, J. vom Brocke, P.-M. Léger, A. B. Randolph, & G. R. Müller-Putz (Eds.), *Information Systems and Neuroscience* (pp. 301-309). Springer International Publishing. https://doi.org/10.1007/978-3-031-13064-9_30

Sengupta, A., Dasgupta, A., Chaudhuri, A., George, A., Routray, A., & Guha, R. (2017). A Multimodal System for Assessing Alertness Levels Due to Cognitive Loading. *IEEE Transactions on Neural Systems and Rehabilitation Engineering: A Publication of the IEEE Engineering in Medicine and Biology Society*, *25*(7), 1037-1046. https://doi.org/10.1109/TNSRE.2017.2672080

Shafiei, S. B., Shadpour, S., & Mohler, J. L. (2024). An Integrated Electroencephalography and Eye-Tracking Analysis Using eXtreme Gradient Boosting for Mental Workload Evaluation in Surgery. *Human Factors*, 00187208241285513. https://doi.org/10.1177/00187208241285513

Singh, G., Chanel, C. P. C., & Roy, R. N. (2021). Mental Workload Estimation Based on Physiological Features for Pilot-UAV Teaming Applications. *Frontiers in Human Neuroscience*, *15*. https://doi.org/10.3389/fnhum.2021.692878

Yang, Y., Chen, Y., Wu, C., Easa, Said. M., Lin, W., & Zheng, X. (2020). Effect of highway directional signs on driver mental workload and behavior using eye movement and brain wave. *Accident Analysis & Prevention*, *146*, 105705. https://doi.org/10.1016/j.aap.2020.105705

Zaky, M. H., Shoorangiz, R., Poudel, G. R., Yang, L., Innes, C. R. H., & Jones, R. D. (2023). Increased cerebral activity during microsleeps reflects an unconscious drive to re-establish consciousness. *International Journal of Psychophysiology*, *189*, 57-65. https://doi.org/10.1016/j.ijpsycho.2023.05.349

Zaky, M. H., Shoorangiz, R., Poudel, G. R., Yang, L., & Jones, R. D. (2021). Investigating the neural signature of microsleeps using EEG. *2021 43rd Annual International Conference of the IEEE Engineering in Medicine & Biology Society (EMBC)*, 6293-6296. https://doi.org/10.1109/EMBC46164.2021.9630401

Zandi, A. S., Quddus, A., Prest, L., & Comeau, F. J. E. (2019). Non-Intrusive Detection of Drowsy Driving Based on Eye Tracking Data. *Transportation Research Record*, *2673*(6), 247-257. https://doi.org/10.1177/0361198119847985

Zhang, H., Dong, E., Tong, J., Yang, S., & Du, S. (2023). Fatigue Driving Detection of EEG Signals by LSTM Deep Neural Network with LPSD and DE. *2023 IEEE International Conference on Mechatronics and Automation (ICMA)*, 1108-1112. https://doi.org/10.1109/ICMA57826.2023.10216090

Zhu, H.-X. (2021). EEG Functional Connectivity Predicts Continuous Fatigue Levels During Underload Task. *2021 International Conference on Artificial Intelligence and Electromechanical Automation (AIEA)*, 322-327. https://doi.org/10.1109/AIEA53260.2021.00075
