# Supplementary material for: Combining EEG and eye-tracking for cognitive and physiological states monitoring: a systematic review
Source: Front Neuroergon. 2026 Jan 29;6:1736672. doi: 10.3389/fnrgo.2025.1736672 (PMC12895110; doi:10.3389/fnrgo.2025.1736672)
Supplement: Supplementary file 2 [file Table_2.docx]

|  |  | **Stress** | **Fatigue** | **Mind wandering** | **Vigilance** | **Drowsiness and sleep** | **Mental workload** |
| --- | --- | --- | --- | --- | --- | --- | --- |
|  | **n studies (*)** | 2 | 9 | 3 | 7 | 6 | 22 |
| **ET** | Gaze metrics | - | 1 (11) | **2 (67)** | - | **6 (100)** | 3 (14) |
|  | Fixation metrics | - | 1 (11) | 1 (33) | **4 (57)** | 1 (17) | **13 (59)** |
|  | Saccade metrics | - | 1 (11) | - | **4 (57)** | 2 (33) | 7 (32) |
|  | Scan & Pattern-based metrics | **2 (100)** | 3 (33) | - | - | - | 6 (27) |
|  | Eyelid Openness-Closure/PERCLOS | - | **5 (56)** | 1 (33) | **4 (57)** | **5 (83)** | 2 (9) |
|  | Blinks | - | 1 (11) | 1 (33) | **5 (71)** | 3 (50) | 7 (32) |
|  | Pupil diameter/size | - | 2 (22) | **2 (67)** | 2 (29) | 1 (17) | **13 (59)** |
|  | Pupil dilation | - | - | - | - | - | 3 (14) |
|  | Pupillometry-based ICA | - | - | - | - | - | 1 (5) |
| **EEG** | Frequency bands metrics | **2 (100)** | **7 (78)** | **2 (67)** | **7 (100)** | **6 (100)** | **20 (91)** |
|  | Region contributions | - | - | 1 (33) | - | - | - |
|  | Time-based metrics (P3 amplitude) | - | 1 (11) | - | - | - | - |
|  | Entropy metrics | - | 3 (33) | - | - | - | 1 (5) |
|  | Complexity metrics | - | - | - | - | - | 1 (5) |
|  | EEG Network Metrics | - | - | - | - | - | 1 (5) |
|  | EEG index based on as-SWLDA | - | - | - | 1 (14) | - | 2 (9) |
| (*) The number of studies that have extracted each metric is accompanied by a number in brackets indicating the percentage relative to the total number of studies within each condition. Metrics present in at least 50% of studies are highlighted in bold. A color scale has been applied, with lighter shades representing lower percentages and darker shades representing higher percentages. | | | | | | | |
