# Supplementary figures and images for: Combining EEG and eye-tracking for cognitive and physiological states monitoring: a systematic review
Source: Front Neuroergon. 2026 Jan 29;6:1736672. doi: 10.3389/fnrgo.2025.1736672 (PMC12895110; doi:10.3389/fnrgo.2025.1736672)

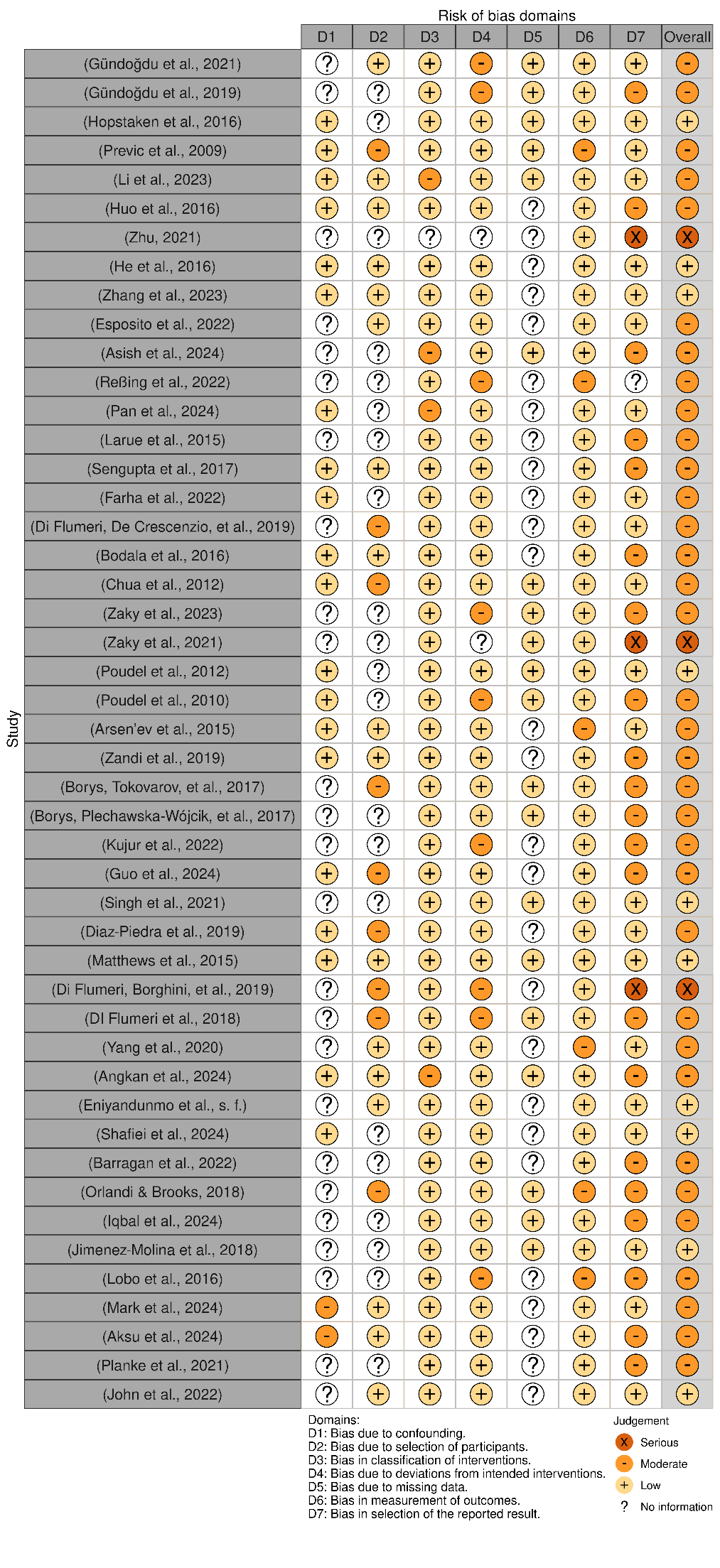

Supplement: Supplementary file 3 [file Table_3.docx]
